# Hotspot movement of compound events on the Europe continent

**DOI:** 10.1038/s41598-023-45067-6

**Published:** 2023-10-23

**Authors:** Smit Chetan Doshi, Gerrit Lohmann, Monica Ionita

**Affiliations:** 1 Alfred Wegener Institute Helmholtz Center for Polar and Marine Research, Bremerhaven, Germany; 2 Physics Department, University of Bremen, 28359 Bremen, Germany; 3 Forest Biometrics Laboratory—Faculty of Forestry, “Ștefan cel Mare” University of Suceava, Universității Street, no. 13, 720229 Suceava, Romania

**Keywords:** Atmospheric science, Atmospheric dynamics, Attribution, Environmental health, Climate-change mitigation, Climate change, Environmental impact, Natural hazards

## Abstract

Climate indices are often used as a climate monitoring tool, allowing us to understand how the frequency, intensity, and duration of extreme weather events are changing over time. Here, based on complex statistical analysis we identify highly correlated significant pairs of compound events at the highest spatial resolution, on a monthly temporal scale across Europe. Continental-scale monthly analysis unleashes information on compound events such as high-risk zones, hotspots, monthly shifts of hotspots and trends, risk exposure to land cover and population, and identification of maximum increasing trends. While there are many studies on single or compound climate extremes there are only a few studies that addresses the relationship between pairs of hazards, the incorporation of bioclimatic indices, the determination of a grid best-fit copula approach, and the outlining relevance of this work of compound event risks with exposures. In this respect, here, using 27-bivariate and 10-trivariate copula models, we show that the different hazard pairs have high combined risks of indices related to radiation, temperature, evapotranspiration, bioclimatic-based indices, such as the universal thermal climate index, wind chill index, and heat index, mainly over the northern and eastern European countries. Furthermore, we show that over the last 7 decades, agricultural and coastal areas are highly exposed to the risks of defined hotspots of compound events. In some of the hotspots of compound events-identified by clusters, there is no monthly shifts of hotspots, leading to higher impacts when compounded. Future work needs to integrate the framework and process to identify other compound pairs.

## Introduction

Extreme weather events, such as heatwaves, droughts, floods and storms (including cyclones) have become more frequent and severe in recent years due to the impact of climate change (IPCC, 2021 and the references therein). Extreme events are of major interest due to their potential to produce significant harm and repercussions on people, infrastructure, and environment^1^. However, we still need a better overview and an in-depth understanding of what happens when two or more of these extreme events occur simultaneously, creating what’s known as a compound extreme event. Compound extreme events can result in devastating consequences for human and natural systems, and understanding their likelihood and impact is crucial for effective risk management and decision-making^2^. Compound events which are two or more events occurring concurrently or consecutively would increase the impacts and cause major extreme consequences that may not necessarily be extreme occurrences independently^3–6^. For example, in central Europe and western Russia during 2003 and 2010, severe drought conditions concurrent with extreme heat waves produce more damage than either excessive heat or extreme dryness would have alone^7^. Other compound event examples includes April 2021 and 2022, with Central Europe experiencing consecutive frost days following warm spring which highly impacted the crop yield^8^. Experience of compound floods due to coincidence of storm surges, waves, tides, precipitation and discharges of river are already identified in many locations of Europe (e.f. over the period 1870–2016, 23 compound flood events were recorded^9^). Extratropical cyclones were identified due to co-occurrences of precipitation and wind across northwestern central Europe during winter season^10^, where events in Great Britain showcased a time difference of 0 to 13 days between peak discharge and extreme wind^11^. Further, the latest IPCC report about “weather and climate extreme events in changing climate” has mentioned that the likelihood of compound events has probably grown in past and will continue to rise with further climate change^12^. It has been suggested that heatwaves and droughts are occurring more often, with hot, dry, and windy events leading to long-lasting fire hazards. Moreover, the number of compound flooding due to extreme rainfall, storm surge, and river discharges also increased^12^. Hazards warnings can be evaluated by climate and weather variables^13^. Numerous societally significant extremes are not well represented by a single climatic variable at a defined location and time, which leads us to focus our study to account for multiple climate variables and links between extremes of similar or different types in time and space^14^. An effort is required to comprehend the dependence structure among multiple climate variables through multivariate techniques^15–20^. Copula-based multivariate techniques have garnered the most attention to study the interplay between various climatic variables due to the process of creating joint distribution for different marginal distribution of random variables^21,22^. Nevertheless, is challenging to apply the strategy to a highly multivariate situation due to the fact that the majority of copula dependence models are bivariate with higher dimensions produced by nested bivariate copulas^23^.

The probabilistic behaviour of extreme events has been investigated in previous research works either through univariate^24–26^ or multivariate^13,22,27^ approach. Previous studies had lacked in one or several aspects such as considering climate extreme indices to determine compound events through multivariate approach by undergoing straightforward application of copula for development of joint distribution, but this might not hold true and may change regionally at different spatial and temporal scales. Defining correlation coefficient only using one technique to indicate the climate indices dependence strengths between one another can diverge too far from reality in evaluation. There are families of copula and selection of copula methods based on the goodness-of-fit, which is commonly considered the best approach in previous published papers^27,28^, but whole study region considering each grid needs to be verified for a proper selection of copula. Recent literature review^29^ had highlighted the missing information gaps of covering all the aspects such as dependence of multiple climatic variables, pre-treatment of data, copula selection and statistical examination of fits of the selected copula. It is essential to define the ties (data with similar rank) or autocorrelation and stationarity between the climate extreme indices but smaller temporal scales such as monthly analysis using daily data overcomes the essential requirement as it is less likely to be observed^29^. Most of the compound events are linked with joint probabilities of precipitation and temperature based climate indices but it must be acknowledged there are indices which are physiologically relevant and needs to be assessed in these combination^30–33^. Comparing multiple climate indices, gains added understanding of the climate system and connection through different sectors. With the available daily weather data and known possible indices—74 monthly spatial climate indices are computed which covers multidisciplinary range of indices with integration of bioclimatic indices that is often neglected. Bioclimatic indices, which indicate how the climate affects human physiology, need also to be considered as they vary both seasonally and regionally in Europe^34^. For the temporally compounding of clusters, an identified gap based on persistence of co-occurrences of compound events requires further study^35^. Persistence refers to climatic events that occur in same location for a consecutive time span^36^. This hotspot identification is important from two reasons: first, the identified hotspot have an increased vulnerability even though not extreme in nature, and second if there are co-occurrences at one specific zone than there will be more limited time to make mitigation decision due to similar or different type of compound event.

Here, we depict the first high resolution European hotspot monthly movement of compound events through bivariate and trivariate copula analysis. Hotspot refers to the high joint probability of the identified compound events. Movement is referred to the path followed by these shifts of hotspots across each month. To understand which could be the new hotspots in future and track its movement, trend analysis with maximum increasing trend was identified along with computing the magnitude of change. Monitoring these hotspots movement can help the stakeholders to allocate the resources effectively and if any strategical planning or management is required than, they could prioritize those areas understanding its impact joint probabilities. For warning systems, hotspot movement acts as a predicted path for the evacuation, mitigation or preparation purposes. If previous mitigated regions underlying causes are unidentified, it can relate to the hotspot compound events. Initially hotspots and later on movement through patterns for various compound events are determined by applying a joint copula distribution and evaluation of the joint probabilities are based on the combination of these 74 climate indices. Climate indices are downscaled based on the dependence strength determined by the correlation coefficient from three different techniques and determining the best-fit copula for each of the grid individually from the families of copula for further analysis. This research analyses gaps of missing information^29^ such as consideration of climate indices, ties, best-fit copula selection, secondly the computation possible in terms of climate indices from available daily data over a longer period (72 years) and thirdly representation of hotspots plus movements of compound event for Europe at a monthly scale. The compound pairs we would discuss check three criteria (i) high correlation value > 0.6 and < − 0.6 for each month (ii) significance value < 0.05 (iii) climate indices computed from different variables (e.g. precipitation, minimum and maximum temperature, radiation, etc.). The graphical representation and analysis would involve best-fit copulas, hazard maps, patterns of hotspots, and risk analysis in conjunction with population density and landuse maps for highly dependent combination of climate indices responsible for compound events, which will be of high importance to many stakeholders across the European region. Based on the interest of the stakeholder these relationships would be useful for estimating the risk of an impact^13^.

## Results and discussion

### Determining compound events

**Figure 1:**
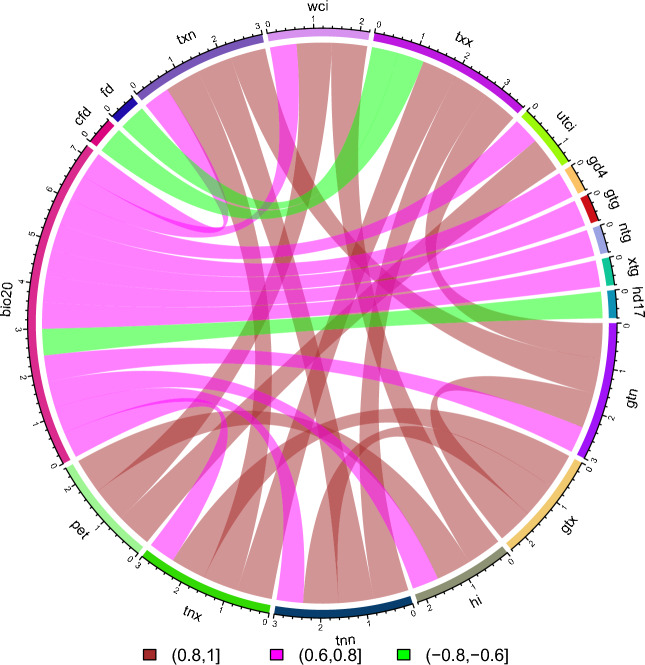
Compound event pairs using correlation coefficient values. Indicating compound event pairs with strong positive (maroon and pink), negative (green) correlation coefficient values, and significance level < 0.05 obtained from 74 climate indices, Pearson, Kendall, and Spearman analysis.

To determine the compound event pairs for bivariate and trivariate analysis it is necessary to first evaluate the correlation and significance values of various combinations of the 74 climate indices (abbreviation elaboration in Table 1) using Pearson, Kendall and Spearman correlation methods. On the basis of climate indices computed, 5402 pairs of combination could be produced, with a wide range of computed values. The correlation and significance values for each method and each month can be visualized in Supplementary Fig. S1. Out of all potential combinations of climate indices and based on the 3 criteria defined above, we look for further analysis of 27 combinations of bivariate analysis and 10 combinations of trivariate analysis. The hazard pairs for each month that have a positive correlation of greater than 0.80 are gtn and gtx, gtn and txn, gtx and tnn, gtx and tnx, hi and wci, tnn and txn, tnn and txx, tnx and txn, tnx and txx, gtn and txx, hi and pet, pet and wci, pet and utci; pairs with correlation greater than 0.60 are bio20 and gd4, bio20 and gtg, bio20 and gtn, bio20 and hi, bio20 and ntg, bio20 and tnn, bio20 and txn, bio20 and utci, bio20 and wci, bio20 and xtg, bio20 and tnx; and the pairs of bio20 and hd17, cfd and txx, fd and txx (see Data and method for the definition of each index) have correlation less than 0.60. All the aforementioned pairs of climate indices have significance level less than 0.05. These pairs act as a base step for further bivariate analysis and for trivariate analysis. The similar pairs are correlated resulting in a combination of hi, pet and wci for correlation greater than 0.80; bio20, txn and gtn, bio20, wci and hi, bio20, txn and tnn, bio20, tnx, and txn, bio20, gtn and txx, bio20, tnn and txx, bio20, tnx and txx for correlation greater than 0.60; and correlation less than 0.60 results in cfd, txx and bio20, fd, txx and bio20. Figure 1 depicts the maximum correlation value of climate indices pairs using three different techniques. There are compound event pairs which are identified that could be opposite in nature such as hi and wci. Statistically it makes a pass but the physical meaning retains to the rare event, which occurs during transition of winter to summer or vice versa perceived by humans. The winter events perceived in Europe were linked to winter cold spells in North America; the wind related indices bivariate pairs might have relevance with the compound extreme pan-Atlantic^37^. Compound pairs of low radiation and high temperature indices can be a major limiting factor for the crop yield^38^. Linkages between heat and human health has raised awareness, thus the pairs of utci, wci, and hi in relation to temperature and radiation that compounds would help to estimate humans feel with varietal climatic conditions^39^. Interdependence of variables of compound events can result from a number of interacting physical processes such as regional sensitivity to global warming, circulation patterns (high or low pressure system)^40^ or natural cycles (El Nino – Southern Oscillation, ENSO)^41^.

**Table 1:** List of climate indices computed for Europe (1950–2021).

ID	Indices name	Definitions	Units
Drought indices			
PHDI	Palmer hydrological drought index	Time series of palmer hydrological drought index	
scPDSI	Self-calibrated palmer drought severity index	Time series of self-calibrated palmer drought severity index	
spi-1	Standardized precipitation index 1	Standardized precipitation index calculated at 1-month time scale	
spi-3	Standardized precipitation index 3	Standardized precipitation index calculated at 3-month time scale	
spi-6	Standardized precipitation index 6	Standardized precipitation index calculated at 6-month time scale	
spi-9	Standardized precipitation index 9	Standardized precipitation index calculated at 9-month time scale	
spi-12	Standardized precipitation index 12	Standardized precipitation index calculated at 12-month time scale	
spi-24	Standardized precipitation index 24	Standardized precipitation index calculated at 24-month time scale	
spei-1	Standardized precipitation-evapotranspiration index 1	Standardized precipitation-evapotranspiration index calculated at 1-month time scale	
spei-3	Standardized precipitation-evapotranspiration index 3	Standardized precipitation-evapotranspiration index calculated at 3-month time scale	
spei-6	Standardized precipitation-evapotranspiration index 6	Standardized precipitation-evapotranspiration index calculated at 6-month time scale	
spei-9	Standardized precipitation-evapotranspiration index 9	Standardized precipitation-evapotranspiration index calculated at 9-month time scale	
spei-12	Standardized precipitation-evapotranspiration index 12	Standardized precipitation-evapotranspiration index calculated at 12-month time scale	
spei-24	Standardized precipitation-evapotranspiration index 24	Standardized precipitation-evapotranspiration index calculated at 24-month time scale	
WPLM	Weighted palmer drought severity index	Time series of weighted palmer drought severity index	
Global radiation			
bio20	Mean radiation	Average daily global radiation	W/m^2^
Multi-element indices			
hi	Heat index	Combines air temperature and relative humidity to determine the human-perceived equivalent temperature	
mi	Mould index	Combined air temperature and relative humidity affecting growth rate of mould. Number of days with a relative humidity over 90% and temperature falls over 10 °C	
pet	Reference evapotranspiration	Using Hargreaves method	mm/day
utci	Universal thermal climate index	Air temperature of reference condition causing the same model response as actual conditions	
wci	Wind chill index	Combines air temperature and wind speed; lowering of body temperature due to the passing-flow of lower-temperature air	
Precipitation indices			
cdd	Longest dry period	Maximum length of consecutive dry days	days
cwd	Longest wet period	Maximum length of consecutive wet days	days
d50mm	Heavy precipitation days	Number of days with precipitation above 50 mm	days
d95p	Very wet days	Days with precipitation > 95th percentile	days
dd	Dry days	Number of days with precipitation less than 1 mm	days
dr1mm	Wet days 1 mm	Total number of wet days ≥ 1 mm	days
dr3mm	Wet days 3 mm	Total number of Wet days ≥ 3 mm	days
prcptot	Total precipitation wet days	Precipitation amount on days ≥ 1 mm	mm
r10mm	Days precipitation ≥ 10mm	Days with daily precipitation amount ≥ 10 mm	days
r20mm	Days precipitation ≥ 20mm	Days with daily precipitation amount ≥ 20 mm	days
r95tot	Percentage precipitation of very wet days	Precipitation at days exceeding the 95th percentile divided by total precipitation	%
r99tot	Precipitation fraction extremely wet days	Precipitation at days exceeding the 99th percentile divided by total precipitation	%
rti	Total precipitation	Total amounts of precipitation	mm
rx1day	Maximum precipitation	Highest amount of daily precipitation	mm
rx5d	Maximum 5 days precipitation	Maximum consecutive 5-day precipitation	mm
sdii	Simple precipitation intensity index	Sum of precipitation in wet days (> 1 mm) divided by number of wet days in the period	mm/day
Relative humidity indices			
rh	Mean relative humidity	Average daily relative humidity	%
Sea level pressure indices			
slp	Mean sea level pressure	Average daily sea level pressure	hPa
Temperature indices			
cfd	Maximum consecutive frost days	Maximum number of consecutive days with TN < 0 °C	days
csd	Maximum consecutive summer days	Maximum number of consecutive summer days with TX > 25 °C	days
csdi	Cold spell duration	Count of days with at least 6 consecutive days when daily minimum temperature < 10th percentile	days
dd17	Difference days above/below TX-17 °C	days TX > 17 °C–days TX < 17 °C	days
dtr	Diurnal temperature range	Mean difference between TX and TN	°C
etr	Extreme temperature range	Difference between the maximum TX and the minimum TN	°C
fd	Frost days	Number of days with TN < 0 °C	days
gd4	Growing degree days	Sum of degree days of TG over 4 °C	°C
gtg	Mean TG	Mean of daily mean air temperature (TG)	°C
gtn	Mean TN	Mean of daily minimum air temperature (TN)	°C
gtx	Mean TX	Mean of daily maximum air temperature (TX)	°C
hd17	Heating degree days	Accumulated degree when TG is below 17 °C	°C
id	Ice days	Number of days with TX < 0 °C	days
ntg	Minimum TG	Minimum value of daily mean air temperature	°C
ogs6	Onset of growing season 6 days	Date of the start of the first span with at least 6 days with TG > 5 °C	days
ogs10	Onset of growing season 10 days	Date of the start of the first span with at least 10 days with TG > 5 °C	days
su	Summer days	Number of days with daily maximum temperature > 25 °C	days
tn10p	Percentage of cold nights	Percentages of days with TN < 10th percentile	%
tn90p	Percentage of warm nights	Percentages of days with TN > 90th percentile	%
tnn	Minimum TN	Minimum of daily minimum air temperature	°C
tnx	Maximum TN	Maximum of daily minimum air temperature	°C
tr	Tropical nights	Number of days with TN > 20 °C	days
tx10p	Percentage of cold days	Percentages of days with TX < 10th percentile	%
tx90p	Percentage of warm days	Percentages of days with TX > 90th percentile	%
txn	Minimum TX	Minimum of daily maximum air temperature	°C
txx	Maximum TX	Maximum of daily maximum air temperature	°C
vcd	Very cold days	Days with TN < 1st percentile	days
vdtr	Mean daily difference dtr	Mean absolute day-to-day difference in dtr	°C
vwd	Very warm days	Days with TX > 99th percentile	days
wsdi	Warm spell duration	Number of days which are part of groups of at least 6 consecutive days when TX > 90th percentile	days
xtg	Maximum TG	Maximum of daily mean air temperature	°C
zcd	Zero crossing days	Number of days with TX > 0 °C and TN < 0 °C	days
Wind speed indices			
fg	Mean of daily FG	Mean of daily mean wind strength	m/s
fg6bft	Number of days with FG ≥ 10.8m/s	Number of days with averaged wind ≥ 10.8m/s	days
fgcalm	Calm days	Number of days with averaged wind ≤ 2m/s	days

Overall, from the hazard monthly pairs global radiation indices such as bio20, combined indices such as pet, hi, utci, wci, and some of the temperature indices (cfd, fd, gd4, gtn, gtg, gtx, hd17, ntg, tnn, tnx, txn, txx, xtg) indicates high correlation and significance level less than 0.05 using all three-correlation measures. There are climate indices related to drought, precipitation, relative humidity, sea-level pressure and wind speed which have strong correlation across the list of indices computed but they do not follow throughout annual strong positive or negative correlation hence have been eliminated. However if seasonal or specific month is to be focused then further analysis could be carried out^42^. Supplementary Table S1 indicates those pairs of very high and statistically significant positive (negative) correlation of seasonal and monthly climate indices, which do not fall in the annual cycles. Further, climate indices derived from similar weather data might reveal strong correlation values that must also be eliminated. The strength of correlation values however varies slightly, with Pearson being largest, followed by Kendall and Spearman where the difference are smaller. Characteristics of each correlation technique creates these differences as Pearson assumes that the relationship is parametric between variables and highly sensitive to outliers whereas Kendall and Spearman are non-parametric and robust to extreme values. Our results, depicts that Kendall (tau), Pearson (r), and Spearman (rho) are useful measures for selection of hazard pairs for compound events giving further insights of relationship between climate indices variables under significance value of 0.05. Based on the correlation strength (threshold > 0.60 & < − 0.60) and significance values < 0.05; 27 bivariate and 10 trivariate pairs are highly possible compound events for the European region. The bivariate and trivariate pairs that include wind speed computed indices that is wci and utci, results in less spatial coverage due to the missing observed gridded data (~ 31%), limitation of the risk map coverage related to entire European continent for those indices based compound pairs must be acknowledged in this analysis.

### Risk maps of compound event

**Figure 2:**
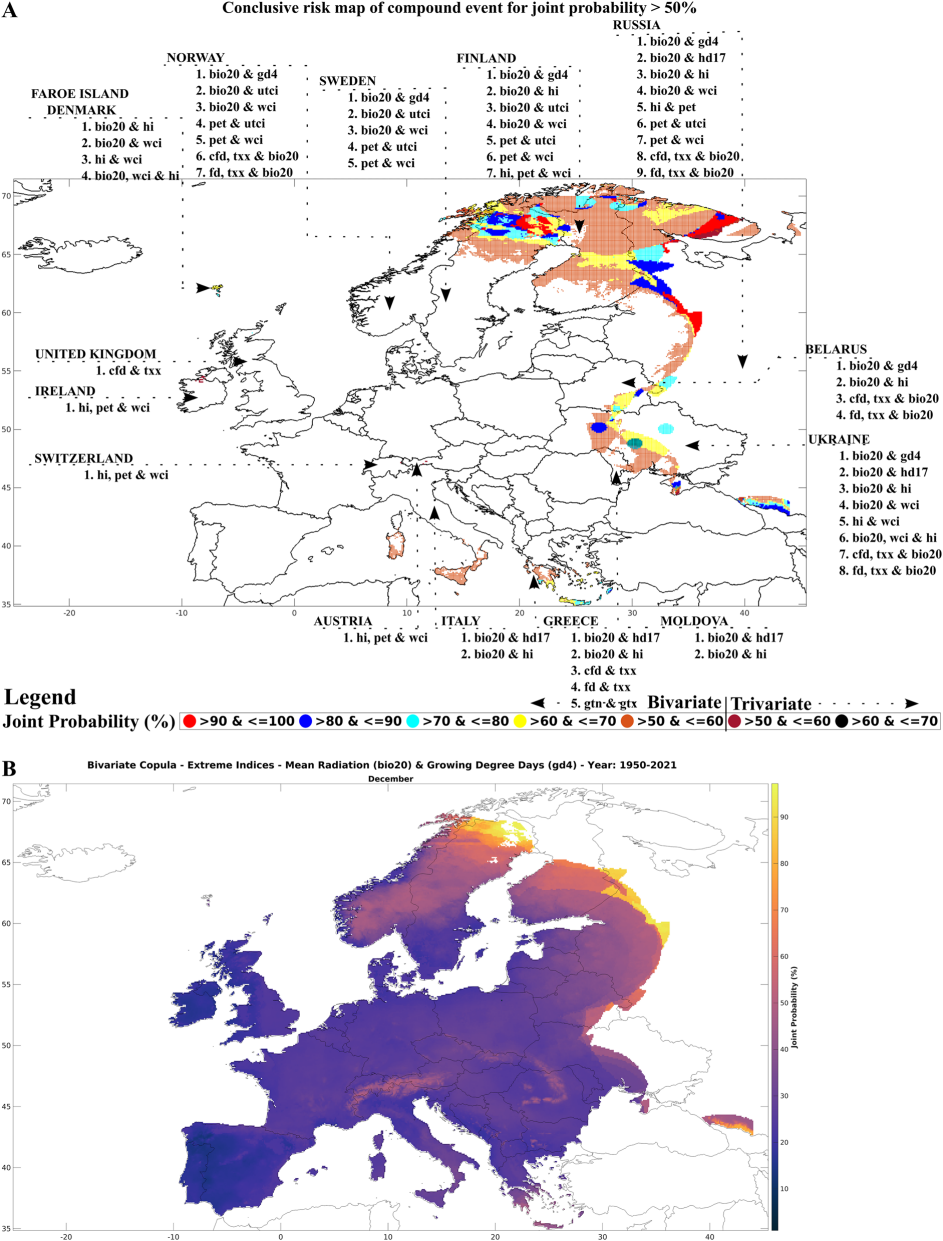
Highlighting Europe’s high risk zone: compound event analysis with joint probability exceeding 50%. (**A**) Identifying the zones, which are of high risk. Red (maroon) colour indicates maximum joint probability for bivariate (trivariate) pair. (**B**) Highest joint probability of 97% for bivariate pair bio20 and gd4 during December to analyse the risks.

Compound event hazard pairs are identified based on the correlation coefficient and using various copulas; (see “Methodology”—“Hazard maps of compound events”). The joint probabilities are computed at a monthly scale for a spatial resolution of 0.1°. In multivariate analysis (bivariate and trivariate) joint probabilities for each grid are based on selection of best-fit copula from the combined statistical indicators Akaike information criterion (AIC), Bayesian information criterion (BIC), Maximum likelihood (MAXL), Nash–Sutcliffe model efficiency coefficient (NSE) and root mean square error (RMSE). Statistical significance (p-value) of the selected copula was tested using Cramér-von-Mises (CvM) hypothesis. Previous research study shows the application of the goodness-of-fit requires at least one performance measure with CvM being utilized often in comparison to Kolmogorov–Smirnov (K-S) statistics^27^. However, CvM tends to be more powerful than K-S test taken with similar approach^43^. To include as a tool for elimination before application of statistical indicators depends on several factor, as the significance value is highly inaccurate for smaller sample size as it plays a significant role in the parametric bootstrap procedure to determine the statistics null distribution^43^. All the monthly risk maps related to bivariate and trivariate compound event pairs are shown in Supplementary Fig. S2. The hotspot analysis leads to the assumption that we are targeting only the highest value (joint probability). However, the next best value could be a region near the hotspot or another area in Europe. Defining the threshold for a hotspot could be a difficult decision if one does not understand the regional functioning, as lower probabilities sometimes lead to high risks and vulnerabilities due to lack of resources and timely decisive management^44,45^. Figure 2 shows a conclusive risk map of compound events occurring in various countries with a joint probabilities > 50%. Once the country and the compound event identified, the specific risk maps can be examined in detail from Supplementary Fig. S2.

**Figure 3:**
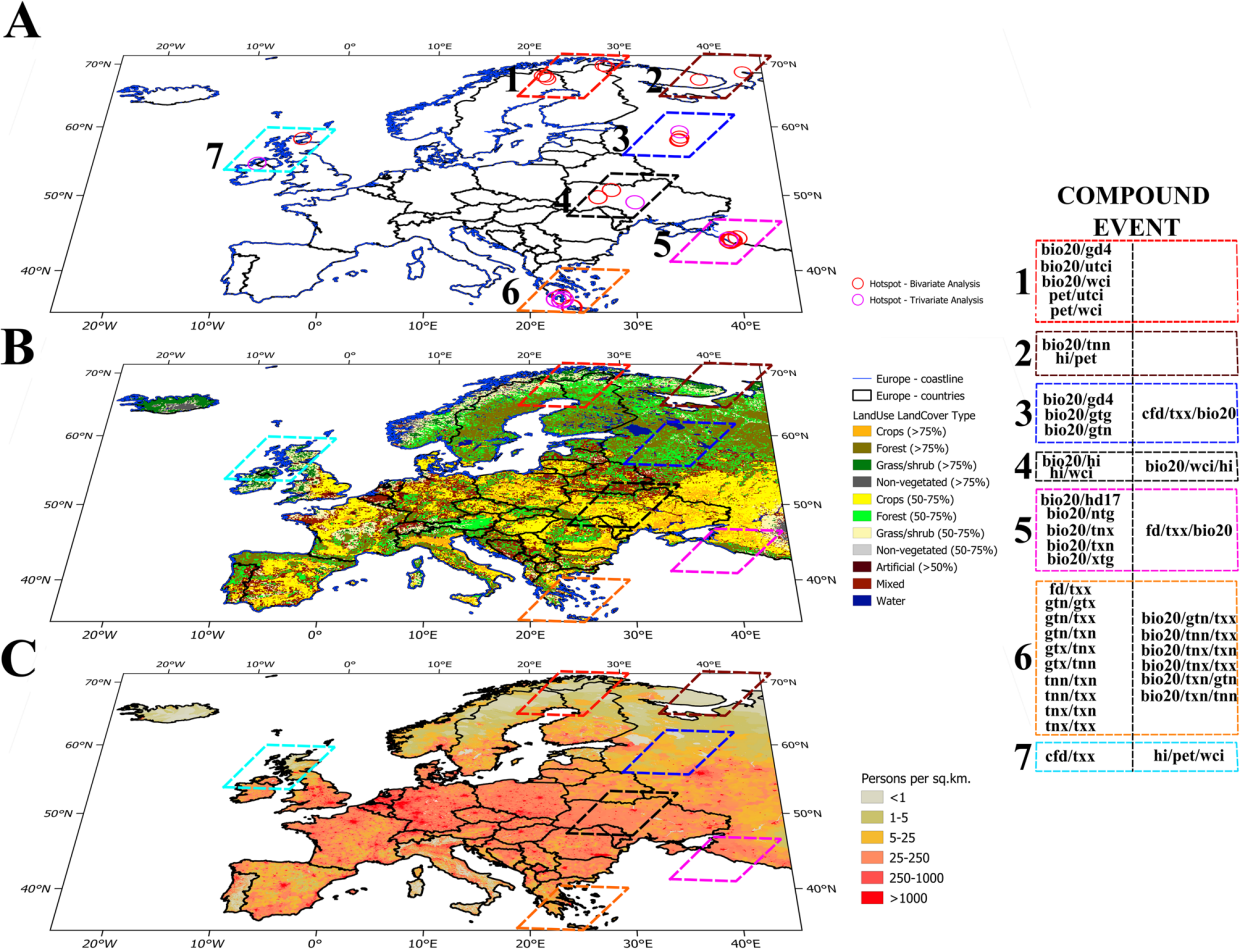
Hotspot of high risks compound events. (**A**) Hotspots of compound events highlighting bivariate and trivariate pairs (**B**) LandUse/LandCover map of Europe (resolution ~ 1 km) (reference: European Environment Agency for coastline demarcation^48^; Food and Agriculture Organization for landcover map^49^) (**C**) Population density map (resolution ~ 1 km) (reference: socioeconomic data and application center^50^).

Prior research studies^8,42,46,47^ estimated copula selection using one best fit for entire study area; however each grid could represent a different copula fit; this misconception leads to errors which have been highlighted in a recent study^29^. Furthermore, steps of methodology failed to demonstrate best copula method opted was on which statistical indicators as their integration with combining performance of multiple indicators were missing. In our study, we have identified the proper copula, through ranking statistical indicators on a scale of 0 to 1, and assigning this scales based on their performance and cumulating it at the end for each grid. The findings indicate that the copula method at each grid for defined pairs of compound event varies. If the values are averaged, for each of the compound event across Europe than Gumbel method is best fit for capturing maximum number of outliers and Student-t can capture both the minimum and maximum outliers. Gaussian copula has outperformed in comparison with all the copula methods and can fit maximum values of joint probability based on the quartile range. Supplementary Fig. S3 indicates comparison of various copula approaches through visualization of box plots for all the determined monthly joint probability of compound event pairs. The above statements of using averaged best-fit copula method on whole Europe would be misleading and add uncertainty if every grid is not tested separately to determine joint probability. Figure 3 hotspots for compound events are shown in Supplementary Table S2, along with the month in which they had high joint probabilities, the best-fit copula technique for that particular grid at that time, and the value of joint probability for bivariate and trivariate pairs.

Based on the aforementioned findings, it is evident that the northern and eastern regions of Europe face the highest risk for the derived compound events. Hotspot regions with compound events of high correlation value for each month where the climate monitoring is required are Sweden, Finland, Russia, Ukraine, Greece, United Kingdom and Ireland. Specifically, the combinations of various hazard pairs, such as temperature, radiation, potential evapotranspiration, and bioclimatic indices, pose significant threats to Norway, Sweden, Finland, and Russia. Belarus and Ukraine are at risk of similar combination of compound event pairs except potential evapotranspiration. Southern Europe is also vulnerable, with temperature and radiation based extremes, posing the higher risk. In Italy, additional threats from radiation and bioclimatic indices – heat index occur. In the western part of Europe, the Faroe island are affected by radiation, bioclimatic indices such as heat and wind chill index, while the United Kingdom faces risks associated with temperature and radiation indices. For bivariate pair bio20 and gd4 (hd17), pet and utci (wci) indicates a likelihood of occurrence of > 90%. The upper bound for trivariate pairs is 64% for bio20, wci and hi compound event. Ireland, Switzerland and Austria are at risk from trivariate pairs of potential evapotranspiration, heat index, and wind chill index. The joint probability increases in extent along the adjoining zones as the threshold of joint probability decreases. However, the assumption of considering the threshold > 50% should not be misinterpreted as the impact of lower joint probabilities being ignorable. This is because the compounding effect with other pairs or unidentified pairs could lead to more vulnerability^51^. Therefore, it is crucial to consider all possible combinations of hazards to fully assess the risk posed by compound events in different regions of Europe. Figure 3 displays the countries of European regions where compound events are producing high risks based on the visualization and copula analysis of joint probability.

According to the zonal analysis of hotspot, combined effects of radiation with growing degree days > 4 °C, thermal stress and prevailing wind effects on environment experienced by Kiruna-Sweden. The landuse map reveals that the agricultural sectors of mixed forest and grass in that zone are affected by this phenomenon. Even though there is no significant trend over past 100 years, the ongoing climate change leads to flooding in Sweden, primarily due to temperature acting as a strongest climatic driver resulting in high flows due to snowmelt^52^. Potential evapotranspiration with thermal stress and prevailing wind effects is influencing Northern Lapland and Finland coastal zones of similar landcover type. Various zones of western Russia experience a higher likelihood of extreme events due to radiation, with minimum of daily minimum, mean and maximum temperature, maximum of daily minimum and maximum temperature, mean of daily minimum and mean temperature, growing degree days > 4 °C and heating degree days < 17 °C, heat index and potential evapotranspiration. Western Russia also experiences a higher likelihood of events due to radiation, maximum of daily maximum temperature and frost days. The landcover type for most of the hotspot zone in western Russia is a mixture offorest and grass, with some regions in proximity to the coastline. Frost days and maximum of daily maximum temperature combined effects are prevailing in Inverness-United Kingdom as well whereas Donegal-Ireland experiences trivariate effect of potential evapotranspiration, heat and wind chill index. Landcover for both the event pairs is grass zone and near coastal zone. Zhytomyr (Cherkasy) Oblast located in northern (central) Ukraine are likely to be impacted by combined effect of radiation with heat index and wind chill index. Various locations in the Greece are likely to be influenced by bivariate and trivariate pairs of minimum, mean and maximum of daily minimum, mean and maximum temperature, radiation and frost days. The areas of Ukraine and Greece impacted are urban and crop areas. Population density map indicates that most of the regions where hotspots are marked lies under a low-density class. However, regions in proximity to the hotspots especially the southern Europe could lead to more distress condition as that zone is under high population density class. There are different seasonal or monthly compound event pairs (Supplementary Table S1) but the focus was on pairs that have a high correlation value (i.e. > 0.8 and < − 0.8) and is statistically significant (p-value < 0.05) throughout the annual period for this hotspot zones of high priority as monthly shifts could be tracked.

To outline the relevance of this work, we need to further access the compound events defined hotspot in connection to ground reality. The combined effect of bio20 and gd4 on the agricultural sector influences crop growth development, which in turn affects landuse strategies. For instance, stakeholders in Kiruna, Sweden, have declared a need for a change in the landuse plans through animal footprints of reindeer herding across the Northern Sweden affected by rough weather change^53^. Urban designs proposals for the region must also consider thermal comfort^54^ that could be altered by the combined effect of bio20 with utci and wci. Changes in the dynamics of the treeline ecotone for various species has been discussed with underlying cause mentioned as global warming^55^, but the combination of pet with utci and wci could have influenced the scenario. Western Russia has combined effects of temperature, heat, radiation and potential evapotranspiration, which could be responsible for hot and dry compound events^56^. Further, these effects could also be underlying reason for a drop of more than 70% in wheat producing at oblasts affecting food security and agricultural impacts^57^. It has already proven that the drought event of Western Russia had developed due to combination of heat and increased evaporative demand^58^. Zhytomyr Oblast, Ukraine recently has experienced wildfire events where air quality change and episodes of dust storm were major reason for this extreme event^59^ and the hazard linked with it suspects towards combination of radiation, heat index and wind chill index. In Greece similar extreme events took place risking infrastructure which compounded by ecological responses through increased crop growth leading to more vulnerable situation than expected^60^. Inverness, United Kingdom experiences increased ticks with warmer climate^61^ from ecological perspective and vulnerable conditions for population due to coastal erosion^62^ with underlying cause unidentified other than mentioning climate change. Landslides^63^ and wildfires^64^ for last 12 decade has been impacting Donegal, Ireland county and the underlying causes are identified but combined effects identified in this study is still missing to be addressed as a part of the issue. Outlining the relevance of this work signifies the results would have added benefit in the previous researches for identifying the other drivers responsible for hazards. The identified compound event along with joint probability could further mitigate the risk by re-designing the framework plans. Land-use and population density maps would be a key to focus on dividing the sector responsible and the prioritizing the key compound events. The further assessment is based on arbitrary threshold; that is focusing on pairs only where the frequency of occurrence for compound event is > 50%. Further, once hotspot has been analysed a monthly shifts indicates how does the movement of the hotspot occurs across the European region.

### Monthly shifts of compound event hotspot

Supplementary Table S3 presents the statistical performance using various statistical indicators and statistical significance (p-value) for the derived compound event pairs of the selected copula method. From statistical performance highlighted in Supplementary Table S3 it could be stated that AIC, BIC, MAXL, RMSE and NSE for all the selected copula methods outperforms from statistical standards indicating a best fit. Statistical significance was computed for the stakeholders to make a final decision based on the best possible selection of the model with a similar approach to previous study^27,29^. AIC and BIC value are similar for the compound events across all the months, while the copula method varies across each month for same compound event. Some copula families are best to capture extreme joint probabilities due to number of times it repeats for entire study area but an analysis of grid-by-grid is required as it could vary especially if hotspots are to be identified. The goodness of fit misses the study for consideration of p-value to check the statistical significance of theoretical copula fits to empirical copula as an elimination step for selection of copula^43,65^. However, the underlined statistical significance of the hotspots determined was tested through nature of trends where the statistical threshold p-value is < 0.05, indicating the selection of copula fit is correct. A detailed Supplementary Table S4 indicates the monthly movement of hotspots of identified compound event pairs along with direction and magnitude of trend using Mann–Kendall and Sen’s slope approach respectively. Statistical estimation always leads to uncertainties and rather sizeable uncertainties exists in multivariate analysis. Previous studies have addressed this uncertainty based on copula fits and developed procedures to estimate based on parametric or non-parametric bootstrapping^66,67^. This study also misses the uncertainty of the copula model selection, parameter and input data, which needs further investigation to acknowledge the stakeholders how uncertain the hotspot estimates are. In previous studies, it was estimated that with decreasing probabilities, the uncertainty range of the copula parameter estimation and input data for copula are increasing^68^. Further, stronger the heavy tailed marginal distribution such as extreme events, the greater is the uncertainty of the joint distribution leading higher uncertainty in joint probabilities^69^.

**Figure 4:**
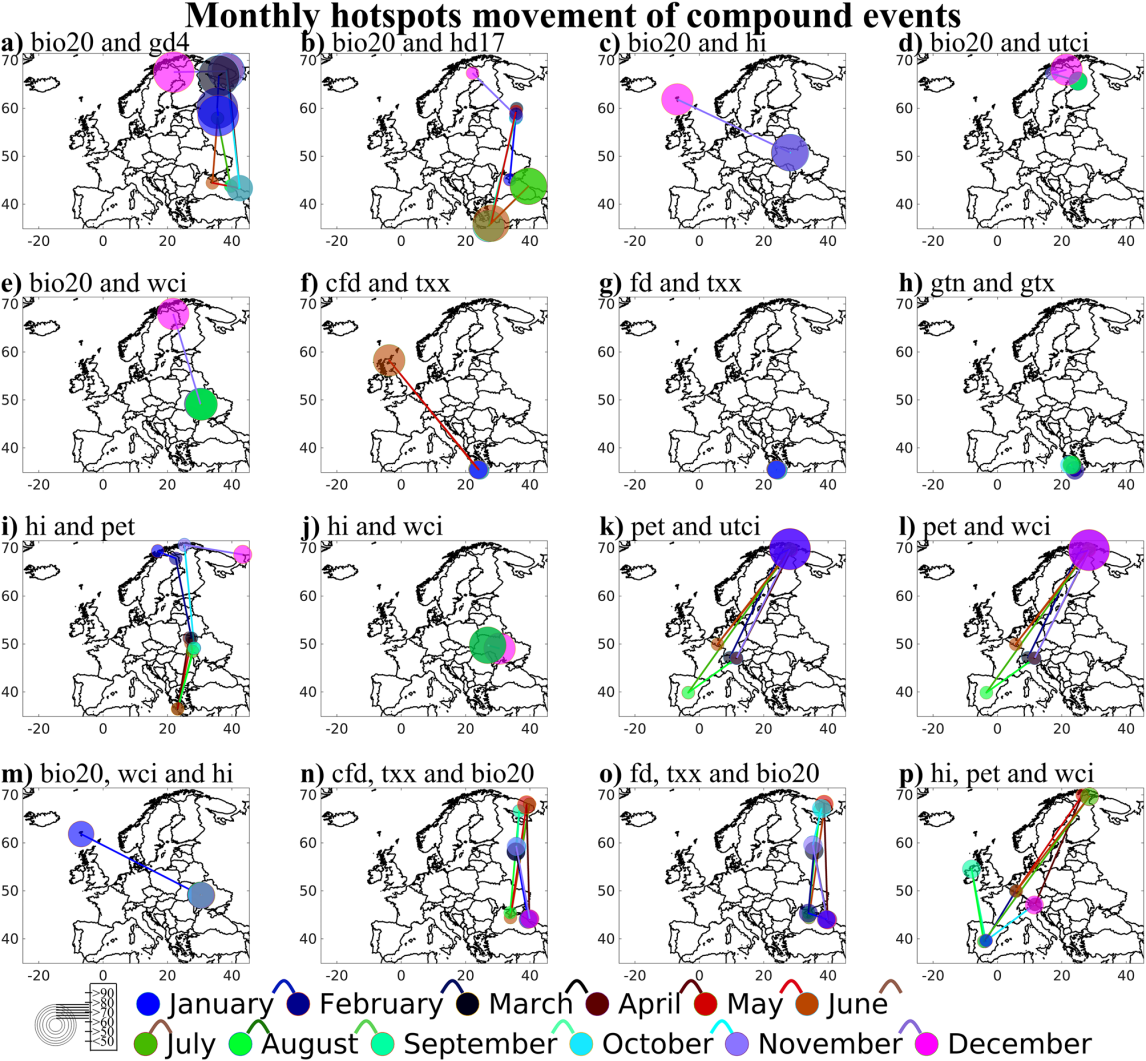
Monthly shifts of hotspots of compound events for joint probabilities > 50%. Movement of hotspot through combination of various bivariate (**a**–**l**) and trivariate analysis (**m**–**p**) where any of the month has threshold of joint probability > 50%. Legends include the circles where the size indicates the joint probability and the color depicts the specific month for the hotspot identified. The connecting line indicates the path of monthly hotspot shifts.

**Figure 5:**
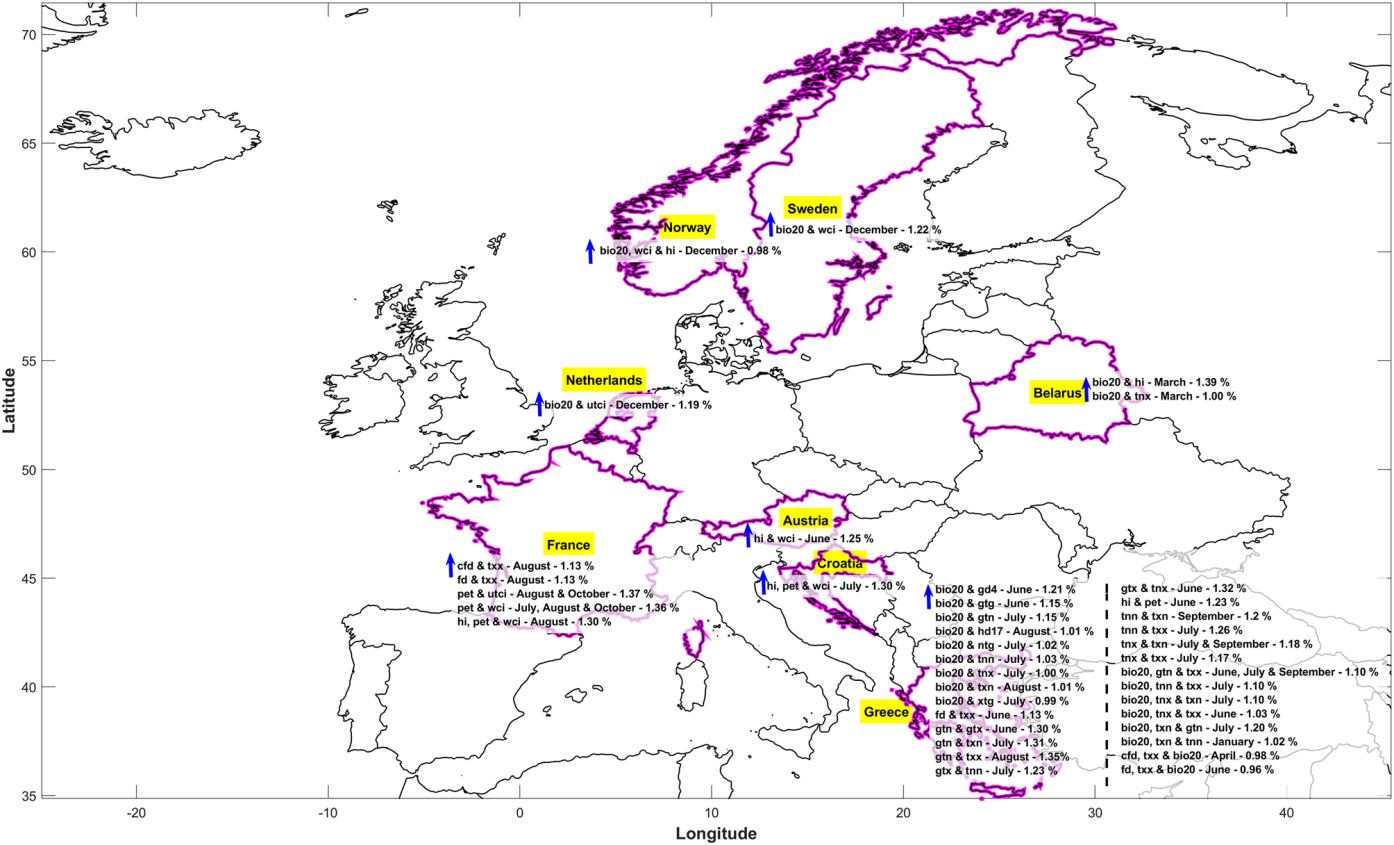
Footprints of future hotspots of compound events. Monitoring the compound events nature and magnitude of trend, (blue) arrow pointing upwards shows maximum future increasing nature and magnitude of trend. Countries are highlighted in yellow with the magnitude of change per month expressed in %.

Figure 4 provides an overview of the movement of hotspot for each month and clusters for similar compound events occurring and timeline for how frequently they repeat each month. This information may be helpful for mitigation purpose and as a deciding factor for stakeholders. The marker sizes on the map indicate the intensity of joint probability, whether there is an influence of this compound event over the region and underlying cause for any potential hazards. Figure 4 shows only the pairs where the highest monthly threshold limit of joint probability > 50%. Spatial distribution of identified compound event after application of Mann–Kendall and Sen’s slope is shown in Supplementary Fig. S4 and based on that Supplementary Table S4 is produced at each identified hotspot grids displaying the direction and magnitude of change of the compound events. In addition, it depicts the location where there is maximum increasing trend and warning signs of potential future hazards. This could be considered as a monthly shift of hotspots in future from the previously shown in Figs. 2, 3A and 4. The magnitude of change is expressed as a percentage increase/decrease per month. The hotspot movements for bio20 and gd4 are observed in Russia and Sweden, with Russia showing increasing trend in risks during summer months, and Greece at the similar time has the maximum % increasing trend. In winter, the risk is > 90% for the compound event to occur over Russia, however the warning zone could be Greece in future. Spring season in Russia indicates a decreasing trend of risks due to bio20 and hd17, but Greece during June and September shows an increasing trend. Greece would experience the maximum risk in future due to joint probability increasing each month at least at a rate of more than 0.70%/month for bio20 and hd17 compound events. Ukraine shows a cluster of bio20 and hi compound event with increasing trend but no significant movement across months except during December where Iceland is influenced. For future, Belarus shows highest increasing risk trend of > 1%/month for the compound event pair. Winter, spring, and summer season of Finland (Ukraine) experience a decreasing (increasing) trend of ~ 0.9–1.1%/month joint probability for bio20 and utci (bio20 and wci). Clusters across Finland (Ukraine) indicates prolonged impact but the movement in future could modify. Highest increasing trend ~ 0.9–1.1%/month occurs in France, United Kingdom, Netherlands, Germany, Spain for bio20 and utci whereas for bio20 and wci similar countries along with Hungary and Sweden movement could be possible. Greece for all seasons would experience a risk of compound event related to frost days and maximum temperature. Hotspot shows a minimum increasing risk trend > 0.6%/month in Greece but in future, for some season France could also influence the clusters as it is increasing at a rate ~ 0.9–1.1%/month. Gtn and gtx combined events across Greece is prevalent for each of the months with an increasing trend of risks ranging from ~ 0.6–1.1%/month and this cluster could change for some months to Sweden and France where the risk has been increasing at rate > 0.9%/month. Hi and pet movement varies a lot across the region without following any seasonality of cluster but highest joint probability 50–55% is in Russia with increasing trend of ~ 0.84%/month during December however maximum trend is observed in France ~ 0.89%/month. Countries that are currently encountering the influence of these compound events are Norway, Sweden, Ukraine, Greece, Moldova and Russia whereas in future warnings could appear in Italy and France. Hi and wci cluster at Ukraine with no movement throughout the year has high probability ~ 80–90% could be replaced by France in future as the trend is increasing at an alarming rate of > 1%/month. Pet with combination of wci and utci follows similar movement across European countries influencing Finland, Norway, Switzerland, Austria, Belarus and Spain with highest risk in December of > 90% probability. Maximum increasing trend with a magnitude of > 1.20%/month is observed in France during summer season. For trivariate pairs clusters forms at Ukraine region for compound event bio20, wci and hi and for various months, the maximum increasing trend is variable across various regions such as Sweden, Spain, France, Ireland, Belarus and Norway with the highest increasing trend at Ireland of ~ 1%/month during August. Cfd (fd), txx and bio20 hotspot movements are within the eastern Europe within Russia and Ukraine. However, in future Greece and Italy could also be under surveillance for the effects due to this compound event as the rates are increasing at ~ 0.7–1.0%/month. Hi, pet and wci is highly variable in movement as the hotspot lies in Spain, Belarus, Norway Luxembourg, Finland, Ireland, Sweden, and Austria that is covering all zones of Europe. With the maximum trend, also, it is variable and the rates are high enough to replace the movements in future with Latvia, Croatia and Hungary. The analysis helps in identifying how the hotspot movement is taking place and which regions are susceptible to the compound event hazard. Stakeholders can make decision based on the compound event pairs which months are under scrutiny, mitigation decision and developing country framework to reduce the risk. Figure 5 is the visualization of Supplementary Table S4 but for compound events that exhibits maximum increasing trend that would replace in future the hotspots of Fig. 2 demarcated.

## Conclusions

In this study We identified 27-bivariate and 10-trivariate pairs of compound events derived from 74 climate indices that exhibit a high correlation (r > 0.6) and statistical significance (p-value < 0.05) at the continental scale. The methodology for determining the joint probability of these hazard pairs using copula covers all the misconception identified in the recent study^29^. The compound events include bioclimatic indices that influence the living organism such as hi, utci and wci, potential evapotranspiration, radiation (bio20) and temperature based indices. The pairs depict correlation strength and significance marker for each month. Due to the fact that they only follow seasonal relationships, the pairs of indices relating to precipitation or drought were not discussed in the current study. Other correlation pairs could be examined in future with similar approach to determine the hotspots and movements within the season of interest. The average best-fit copula method is student-t copula to capture outliers. Considering the values of statistical indicators and the computation time, this approach could be utilized, but to avoid uncertainties, each grid must be tested similar to this study.

Based on the analysis of the compound event pairs derived we conclude:

- Northern and Eastern Europe are highly influenced by the risks due to hazard pairs of indices related to radiation, temperature, evapotranspiration and bioclimatic-based indices, such as the universal thermal climate index, wind chill index, and heat index.
- Agricultural and coastal zones highlighted higher vulnerability compared to urban infrastructure.
- Outlining the relevance of this work, results indicate that the climate change is affecting certain regions but most of the studies fail to indicate the influencing factors for such modification.
- Monthly shifts of hotspots for compound events are clustered for pairs related to bioclimatic indices – hi, utci, and wci, which leads to more risk on living organisms due to compounded effect.
- The trend analysis showcase that the increasing trend might affect the movement and the shifts of hotspot patterns could lead to risk other European countries.

For the future it is advisable to identify a list of compound event pairs that are of high risks and adhere to specific countries to include them as a part of a potential warning system, similar to how ETCCDI has determined a set of 27 core climate indices^70,71^ to monitor occurrence of climate extremes. As an illustration, Sweden and Russia could integrate bio20 and gd4 due to high risk of joint probability and during that period Ukraine and Greece should be closely monitored as it is showing hotspot interference by higher increasing trend during that period. Overall, this study is still challenging in answering the unresolved question of why these compound events pose a high risk to this zone and what mitigation measures are in place to reduce vulnerability to these events when they increase many times over. By understanding the dependence between different variables/climate extreme indices, we can better predict the likelihood of extreme events occurring and develop strategies to mitigate their impact. Moreover, the use of copulas is an important tool for improving our understanding of the complex and interdependent nature of extreme climate events.

## Methods

### Data

The study region focused here is Europe which is the westernmost part of Eurasia and whose climatic data covering the entire European land surface (25° N–71.5° N × 25° W–45° E) is obtained through European Climate Assessment and Dataset (ECA&D) Ensembles daily gridded observation dataset (E-OBS) v25.0e^72^. These data source provides daily gridded observation dataset from 1950 to 2021 (72 years) with resolution ~ 11 km (0.1°) of global radiation, maximum air temperature, minimum air temperature, precipitation, relative humidity, sea level pressure and wind speed which is further utilized to obtain the monthly gridded climate indices. The data source was opted due to the high resolution gridded observational data, requisite frequency of weather data, European area coverage, and significant model validation for climate monitoring^73–75^. The European National Meteorological and Hydrological Services (NMHSs) entity provides the station data; weather data such as wind speed is covered less spatially and global radiation is derived based on in-situ and satellite data—Clouds and the Earth’s Radiant Energy System (CERES). Other sources, such as ERA-5 reanalysis, could be used to obtain complete spatial coverage of wind speed related weather data, but it has been avoided due to different spatial resolution and data source that can further lead to uncertainties and lacking consistencies^76–78^. However, for global radiation users do not have the option to utilize only in-situ observations. 74 climate indices diversified into drought indices, global radiation indices, multi-element indices, precipitation indices, relative humidity indices, sea level pressure indices, temperature indices and wind speed indices which are computed is showcased in Table 1 along with the description of each of them. The computation of climate indices is carried out using R platform “ClimInd” (https://cran.r-project.org/web/packages/ClimInd/index.html) and “scPDSI” (https://cran.r-project.org/web/packages/scPDSI/index.html) package^79,80^. The decision of selection of climate indices were based on potential monthly time-scale from the input weather data. The spatial visualization of all the climate indices across Europe is showcased in Supplementary Fig. S5. Further, the climate indices used in this study cover also all the 27 core extreme climate indices defined by Expert Team on Climate Change Detection and Indices (ETCCDI)^70,71^.

### Methodology

**Figure 6:**
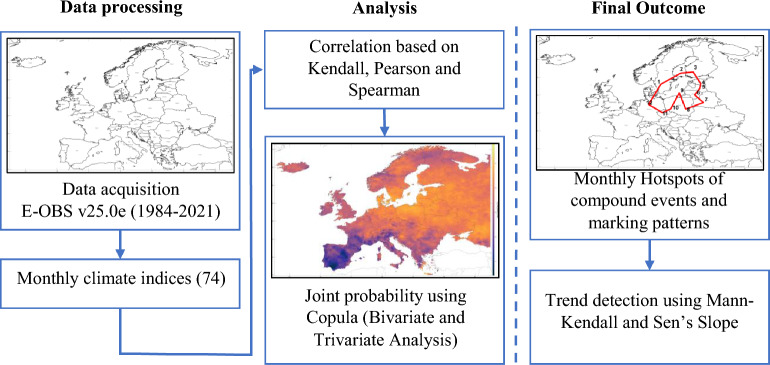
Schematic representation of the methodological framework. The steps taken to determine the monthly hotspots and patterns of the compound events.

This study’s workflow along with strategies used are detailed below and depicted in Fig. 6.

### Data processing

The majority of data acquisition has been covered in the data section, with an important note to consider the uncertainty associated with the global radiation as well as the spatial variability coverage across Europe in relation to wind speed data for the computed climate indices. The daily gridded weather data from E-OBS, when used an input for the requisite R-package results in 74 different monthly climate indices. Mean wind speed (fg), number of days with wind speed ≥ 10.8m/s (fg6bft), and calm days (fgcalm) are climate indices that only use wind speed as input weather data, while universal thermal climate index (utci) and wind chill index (wci) combine it with other weather data. Mean radiation (bio20) is a climate index that only used global radiation as input weather data. Climate indices reference evapotranspiration (pet) further impacting computation of drought indices requires attention through proper selection of empirical method. By using the Hargreaves method which requires only minimum and maximum air temperature as climatological data for the calculation, instead of the Penman–Monteith method in this case, we are able to achieve the full coverage of Europe over uncertain global radiation data and lower spatial coverage due to wind speed data. The Hargreaves method is a recommended approach because the simulations are continuous, the approach is straightforward and produces acceptable results with readily available input data^81^.

### Analysis

#### Correlation of climate indices

Number of correlation coefficients are available based on various statistical hypothesis which are popular to quantify the degree of two variables in relation. There are three of them Kendall, Pearson and Spearman rank correlation coefficient commonly used^82–84^. Pearson is parametric test^85^ and Kendall and Spearman are non-parametric test^86^ to measure the degree of strength and association between climate indices variables. Pearson is utilized with an assumption that data is normally distributed about regression line^85^ whereas Kendall and Spearman are rank-based computation illustrating monotonic relationships^86^. In our situation where there are climate indices which are numerically equal such as zeros, the values are given an arithmetic mean rank numbers related to the ties. Kendall and Spearman have different correlation procedures to handle ties^87^. For 74 climate indices, the correlation values based on each technique were computed using the built-in “corr” function of Matlab. In the documentation for the Matlab function, the formulas for each of the technique have been described^88–91^. The next phase involved choosing the climate indices that had a strong correlation value and were statistically significant. The correlation coefficient value ranges between ± 1; ± shows the correlation’s direction. Climate indices are divided into groups of ± 0.80 to ± 1.00 (very high correlation), ± 0.60 to ± 0.80 (high correlation), ± 0.40 to ± 0.60 (medium correlation), ± 0.20 to ± 0.40 (low correlation), ± 0.00 to ± 0.20 (very low correlation). The statistical significance level (p-value) is set at < 0.05; this means that the null hypothesis is rejected if the targeted statistical correlation reaches statistical significance. With this boundary points framework, the question of correlation strength cannot be justified^92^; however, for further multivariate analysis we are interested in the climate indices with correlation > 0.60, < − 0.60 and significance level (p-value) < 0.05. The correlation and significance values of each methodology would be different, but the combination is based on the union of a subset of the Penman, Kendall, and Spearman methods.

#### Hazard maps of compound events

“Hazard” maps is a visualization of the probability of occurrence that a given natural phenomenon of a given magnitude will occur within a given time frame and given location^93^. In our study, multivariate analysis (bivariate and trivariate) through joint probability is used to visualize the hazard maps of compound events. Joint probabilities are computed statistically through copula function. Early works by Sklar^94^ introduced copula which is a joint multivariate distribution in which the marginal distribution is uniform over range (0, 1) and from which the joint distribution of two or more variables may be derived^95,96^, assuming variables are continuous and time-independent^29^. If R is an n-dimensional cumulative distribution function (CDF) with 1-dimenisonal marginal distribution F_i_(x_i_), then n-dimensional copula (C) exists such that:

1 $$\mathrm{R}\left({\mathrm{x}}_{1},{\mathrm{x}}_{2},\dots ,{\mathrm{x}}_{\mathrm{n}}\right)=\mathrm{C}\left({\mathrm{F}}_{1}\left({\mathrm{x}}_{1}\right),{\mathrm{F}}_{2}\left({\mathrm{x}}_{2}\right),\dots ,{\mathrm{F}}_{\mathrm{n}}\left({\mathrm{x}}_{\mathrm{n}}\right)\right)=\mathrm{C}\left({\mathrm{u}}_{1,}{\mathrm{u}}_{2},\dots ,{\mathrm{u}}_{\mathrm{n}}\right),$$

where (x_1_,x_2_,…,x_n_) are random variables, and (F_1_(x_1_),F_2_(x_2_),…,F_n_(x_n_))-denoted by (u_1_,u_2_,…,u_n_) are marginal distribution for which dependence structure is to be modelled by copula C^97^. Copula distribution is diversified into empirical and theoretical copula. The two types of copulas most frequently used in theoretical work are Elliptical (Gaussian and Student-t) and Archimedean (Clayton, Frank, Gumbel). In this study the joint probability distribution of bivariate and trivariate combinations of climate indices were analysed using Gumbel, Clayton, Frank, Gaussian and Student-t copula. General formula for Elliptical and Archimedean copula are defined below^29,98,99^:

Elliptical copula:

2 $$\mathrm{C}\left({\mathrm{u}}_{1},{\mathrm{u}}_{2}\right)=\upphi \left[{\upphi }^{-1}\left({\mathrm{u}}_{1}\right),{\upphi }^{-1}\left({\mathrm{u}}_{2}\right)\right] \in [\mathrm{0,1}{]}^{2},$$

where, ϕ^–1^ is inverse of univariate marginal distribution, ϕ is multivariate distribution.

Archimedean copula:

3 $$\mathrm{C}\left({\mathrm{u}}_{1},{\mathrm{u}}_{2}\right)=\uppsi \left[{\uppsi }^{-1}\left({\mathrm{u}}_{1}\right),{\uppsi }^{-1}\left({\mathrm{u}}_{2}\right)\right] \in [\mathrm{0,1}{]}^{2},$$

where, ψ^–1^ is pseudo-inverse of generator function, ψ is generator function which is continuous strictly decreasing convex function.

The computation of multivariate joint distribution was carried out using the Statistics and Machine Learning Toolbox of Matlab with ‘copula’ package^100^. Various aspects of the dependence structure are captured differently by various theoretical copula families, with some copulas being better suited to model the overall dependence while others are suited to represent the dependence at the tails of the distribution^101–103^. Main features of probability concentration of various copula such as Clayton in lower tail, Frank in symmetry, Gaussian in symmetry, Gumbel in upper tail, and Student-t in symmetry, upper tail, and lower tail^29^. Assessment of the best-fitted copula based on goodness-of-fit is determined. To identify performance from the families of copula analytical tests such as Cramér-von-Mises (CvM) hypothesis test is one such viable approach^29^. The CvM test^43,104^ was used to evaluate the goodness of fit of theoretical copulas with a lower p-value means we accept the null hypothesis that both samples are drawn from the same underlying distribution and have confidence in the theoretical distribution fit^27^. The p-values for each copula of families for each grid of climate indices compound pair computed joint probability are obtained using Eq. (4)^104^.

4 $${\mathrm{T}}_{\mathrm{c}}=\frac{\mathrm{mn}}{\mathrm{N}}{\int }_{\infty }^{-\infty }[{\mathrm{A}}_{\mathrm{m}}\left(\mathrm{x}\right)-{\mathrm{B}}_{\mathrm{n}}\left(\mathrm{x}\right){]}^{2}{\mathrm{dH}}_{\mathrm{N}}(\mathrm{x}),$$

where criterion of testing to obtain p-value is based on A_m_ and B_n_ which are empirical and theoretical distribution of copula based on compound pairs. H_N_ is empirical distribution function of combined sample, with N = m + n. Different statistical indicators such as AIC, BIC, MAXL, NSE and RMSE are used to determine the best fit from families of theoretical copula in comparison to empirical copula. The formula to compute each of the statistical indicators: AIC^105,106^, BIC^107^, MAXL, RMSE and NSE are defined below.

5 $$\mathrm{AIC}=2\mathrm{D}-2\mathrm{l}=2\mathrm{D}+\mathrm{nln}\left\{\frac{\sum_{\mathrm{i}=1}^{\mathrm{n}}[{\widetilde{\mathrm{y}}}_{\mathrm{i}}-{\mathrm{y}}_{\mathrm{i}}\left(\uptheta \right){]}^{2}}{\mathrm{n}}\right\}-2\mathrm{CS},$$

6 $$\mathrm{BIC}=\mathrm{Dln}\left(\mathrm{n}\right)-2\mathrm{l}=\mathrm{Dln}\left(\mathrm{n}\right)+\mathrm{nln}\left\{\frac{\sum_{\mathrm{i}=1}^{\mathrm{n}}[{\widetilde{\mathrm{y}}}_{\mathrm{i}}-{\mathrm{y}}_{\mathrm{i}}\left(\uptheta \right){]}^{2}}{\mathrm{n}}\right\}-2\mathrm{CS},$$

7 $$\mathrm{MAXL}=-\frac{\mathrm{n}}{2}\mathrm{ln}\left\{\frac{\sum_{\mathrm{i}=1}^{\mathrm{n}}[{\widetilde{\mathrm{y}}}_{\mathrm{i}}-{\mathrm{y}}_{\mathrm{i}}\left(\uptheta \right){]}^{2}}{\mathrm{n}}\right\},$$

8 $$\mathrm{RMSE}=\sqrt{\frac{\sum_{\mathrm{i}=1}^{\mathrm{n}}[{\widetilde{\mathrm{y}}}_{\mathrm{i}}-{\mathrm{y}}_{\mathrm{i}}\left(\uptheta \right){]}^{2}}{\mathrm{n}},}$$

9 $$\mathrm{NSE}=1-\frac{\sum_{\mathrm{i}=1}^{\mathrm{n}}[{\widetilde{\mathrm{y}}}_{\mathrm{i}}-{\mathrm{y}}_{\mathrm{i}}\left(\uptheta \right){]}^{2}}{\sum_{\mathrm{i}=1}^{\mathrm{n}}{\left[{\widetilde{\mathrm{y}}}_{\mathrm{i}}-\overline{{\widetilde{\mathrm{y}} }_{i}}\right]}^{2}},$$

where D is the number of parameters of statistical model, l is the log-likelihood value of best parameter set, ${\widetilde{\sigma }}^{2}=\frac{\sum_{\mathrm{i}=1}^{\mathrm{n}}[{\widetilde{\mathrm{y}}}_{\mathrm{i}}-{\mathrm{y}}_{\mathrm{i}}\left(\uptheta \right){]}^{2}}{\mathrm{n}}$ is gaussian assumption of error residuals, θ is the copula parameter, and cs is a constant. AIC estimates are based on the residual sum of squares instead of maximizing the likelihood function of distribution; the minimum the AIC value the better is the fit of the copula. Similarly, in order to determine the copula of families, it is preferred that BIC have a smaller value, AIC have a higher value, NSE have a higher value near to 1, and RMSE have a lower value near to 0. The method is repeated for each grid in order to calculate the joint probability using each family of copulas and then compare the results with empirical copulas. Based on a variety of statistical indicators, related to each copula family the finalized theoretical copula is the one with the highest total towards goodness of fit. Values from the theoretical copula are close to those from the empirical copulas, they performed well based on statistical indicators used for various climate indices combinations. For various combinations of climate indices that have a correlation > 0.60 and < − 0.60 and significance level (p-value) < 0.05, the joint probability for each grid indicating the best matched copula, is calculated and displayed as a hazard map. Different combinations of the 74 possible climate indices are discussed in the result section within correlation of climate indices para. Best fit copula for the each of the combination using bivariate and trivariate analysis and the joint probability visualization has been discussed in the result section within hotspots and patterns of compound events para.

### Final outcome

#### Defining hotspots and patterns

Copula-based derived joint probabilities of various combined climate indices surpassing the threshold of correlation and significance level for each grid help a stakeholder to determine the frequency of occurrence of that particular compound event for each month. Each grid value of hazard pairs is sorted based on highest to lowest order and a rank is assigned. Further the hotspots are defined based on highest possible frequency of occurrence (rank with number 1 is assigned) across Europe. This process is repeated for each month and for each of the compound events shortlisted from the bivariate and trivariate analysis. Patterns are formed by linking the tagged month of the defined hotspot and additional analyses are based on those patterns, answering the question in the result section: which zones are more vulnerable? For which kind of compound event? And for which month?

#### Trend detection

There are parametric and nonparametric approaches for detecting significant trends in time-series of climate. Non-parametric trend tests need that the data be independent whereas parametric trend test demands data is independent as well as normally distributed^108^. Non-parametric approach Mann–Kendall and Sen’s slope estimator at each grid point was used to assess the trends in the joint probabilities of bivariate and trivariate analysis derived climate indices combinations. Assumption that while computation of trends of the joint probabilities, we assume the stationarity of the climate. Statistical significance of trends estimated using nonparametric Mann-Kendall^109^ method and magnitude of trends were derived using Sen’s slope estimator^110^. The trend analysis that is Kendall’s tau significance and Sen’s slope estimate is computed using R platform “wql” (https://cran.r-project.org/web/packages/wql/index.html) package with function “mannKen”. The formula to determine the trend using Mann-Kendall^90,111^ and Sen’s slope^110^ is defined below:

Mann–Kendall:

10 $$\mathrm{S}=\sum_{\mathrm{i}=1}^{\mathrm{n}-1}\sum_{\mathrm{j}=\mathrm{i}+1}^{\mathrm{n}}\mathrm{sgn}\left({\mathrm{x}}_{\mathrm{j}}-{\mathrm{x}}_{\mathrm{i}}\right),$$

where, n is number of data points, x_i_ and x_j_ are data values in time series and sign function-sgn is given by:

11 $$\mathrm{sgn}\left({\mathrm{x}}_{\mathrm{j}}-{\mathrm{x}}_{\mathrm{i}}\right)=\left\{\begin{array}{c}+1, {\mathrm{x}}_{\mathrm{j}}-{\mathrm{x}}_{\mathrm{i}}>0\\ 0, {\mathrm{x}}_{\mathrm{j}}-{\mathrm{x}}_{\mathrm{i}}=0\\ -1, {\mathrm{x}}_{\mathrm{j}}-{\mathrm{x}}_{\mathrm{i}}<0\end{array}\right..$$

Increasing and decreasing trend is determined by Z_s_ based on positive and negative values obtained respectively. Z_s_ is computed using:

12 $${\mathrm{Z}}_{\mathrm{s}}=\left\{\begin{array}{c}\frac{\mathrm{S}-1}{\sqrt{\mathrm{Var}(\mathrm{S})}}, S>0\\ 0, S=0\\ \frac{\mathrm{S}+1}{\sqrt{\mathrm{Var}(\mathrm{S})}}, S<0.\end{array}\right.$$

Sen’s Slope:

13 $${\mathrm{Q}}_{\mathrm{i}}=\frac{{(\mathrm{x}}_{\mathrm{j}}-{\mathrm{x}}_{\mathrm{i}})}{\mathrm{j}-\mathrm{i}},\mathrm{ i}=\mathrm{1,2},3,\dots ,\mathrm{ N},$$

where N value is sorted for Qi from small to large and then Sen’s slope is obtained based on the two-tailed estimate for the median Qi (Q_med_), which is determined by:

14 $${\mathrm{Q}}_{\mathrm{med}}=\left\{\begin{array}{c}{\mathrm{Q}}_{\frac{\mathrm{N}+1}{2}}, N=odd\\ \frac{{\mathrm{Q}}_{\frac{\mathrm{N}}{2}}+{\mathrm{Q}}_{\frac{\mathrm{N}+1}{2}} }{2}, N=even\end{array}\right. .$$

The outcome from Mann–Kendall would capture the direction of trend that is percentage increasing (positive value) or decreasing (negative value) and Sen’s slope would capture the magnitude of trend that is percentage change in year for the joint probabilities of climate indices resulting compound event over each month for European regions.

### Supplementary Information

Supplementary Figure S1.

Supplementary Figure S2.

Supplementary Figure S3.

Supplementary Figure S4.

Supplementary Figure S5.

Supplementary Table S1.

Supplementary Table S2.

Supplementary Table S3.

Supplementary Table S4.

## Data Availability

All data are from E-OBS (Version 25.0e) used in our analysis are freely available. (https://surfobs.climate.copernicus.eu/dataaccess/)^72^. LandUse/LandCover map of Europe freely available at Food and Agriculture Organization for landcover map (https://data.apps.fao.org/)^49^. Data for coastline demarcation freely available at European Environment Agency (https://www.eea.europa.eu/)^48^. Population density map freely available at socioeconomic data and application center (https://sedac.ciesin.columbia.edu/)^50^. Processed data and code to produce the results are available at: 10.5281/zenodo.10014462.
