# Supplementary material for: Hotspot movement of compound events on the Europe continent
Source: Sci Rep. 2023 Oct 23;13:18100. doi: 10.1038/s41598-023-45067-6 (PMC10593787; doi:10.1038/s41598-023-45067-6)
Supplement: Supplementary file 2 — Supplementary Figure S2. [file 41598_2023_45067_MOESM2_ESM.docx]

**Figure S2: Risk maps of compound event**

1. **Bivariate pairs**


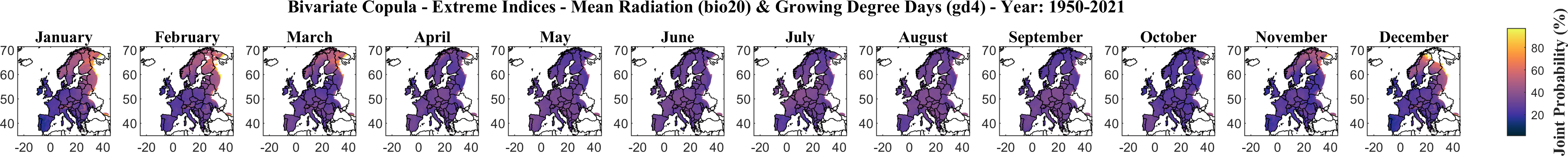

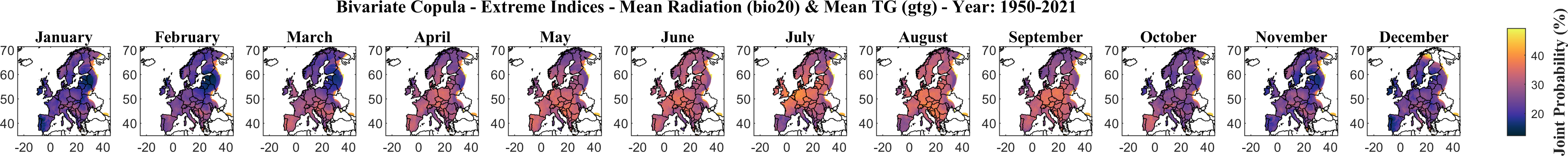

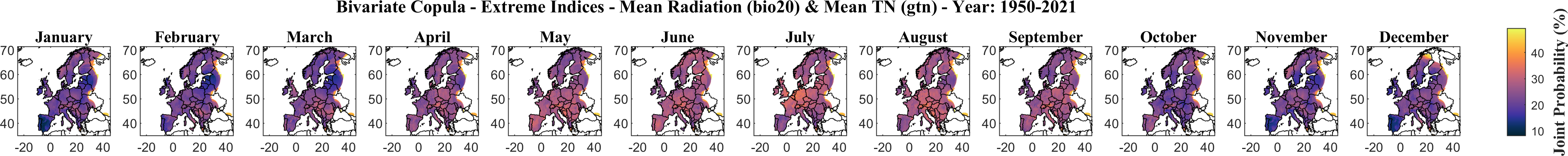

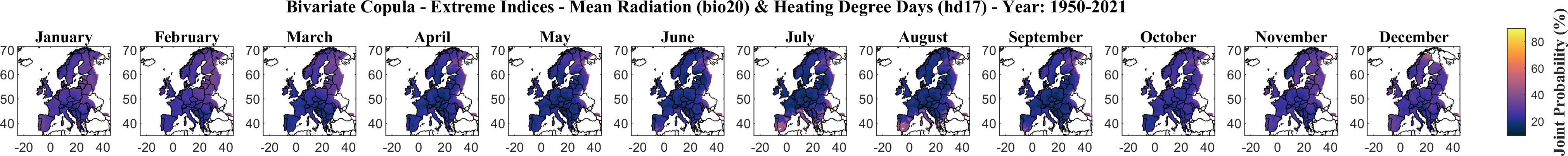

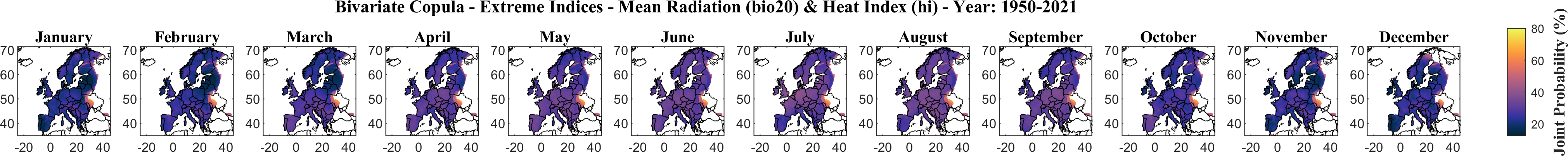

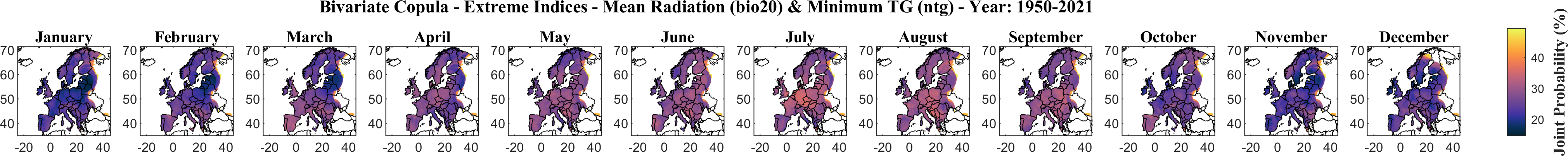

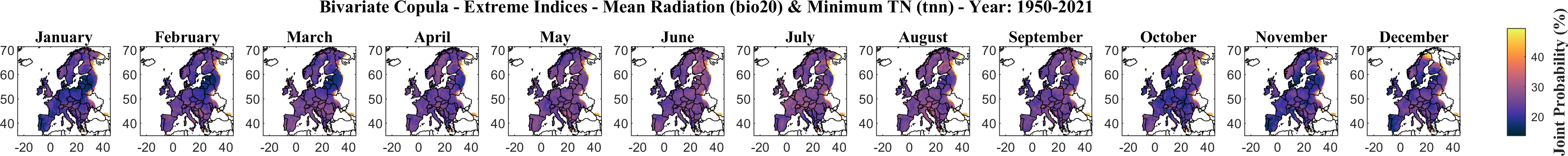

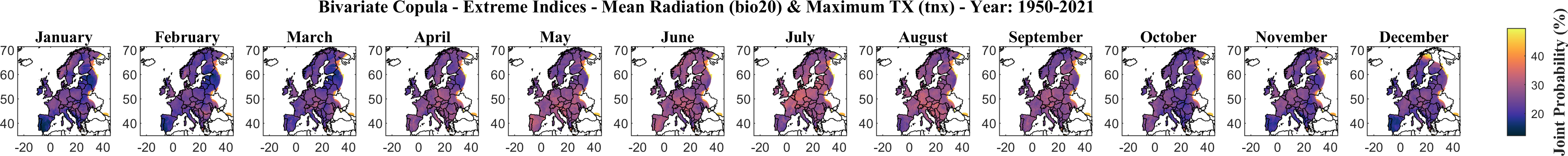

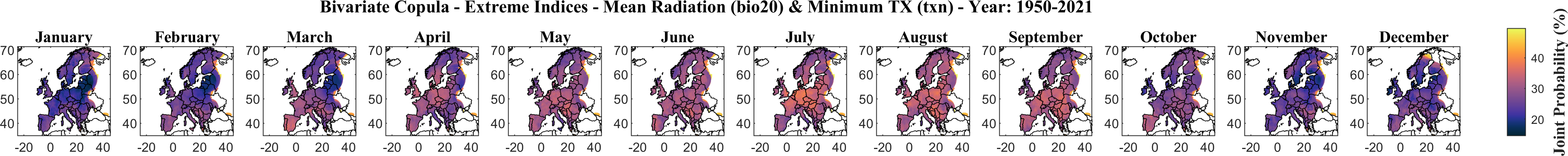

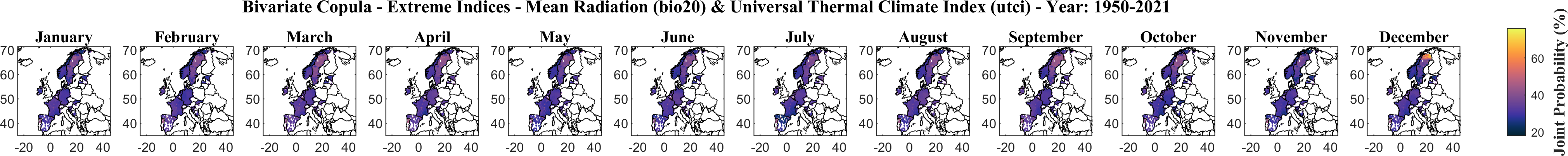

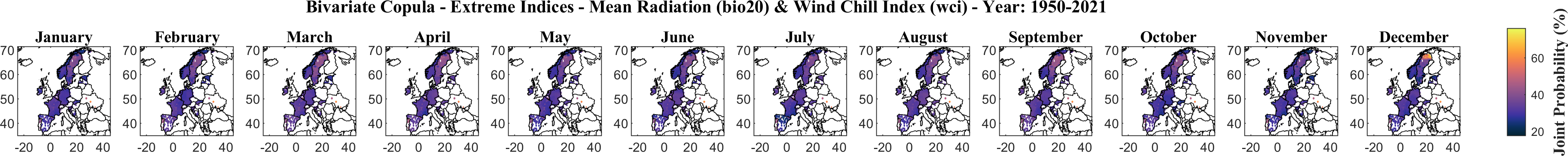

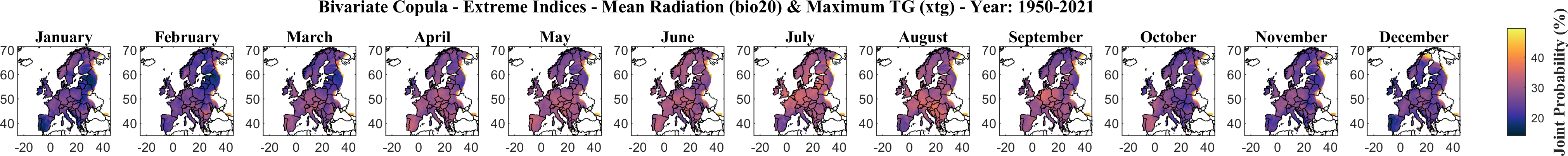

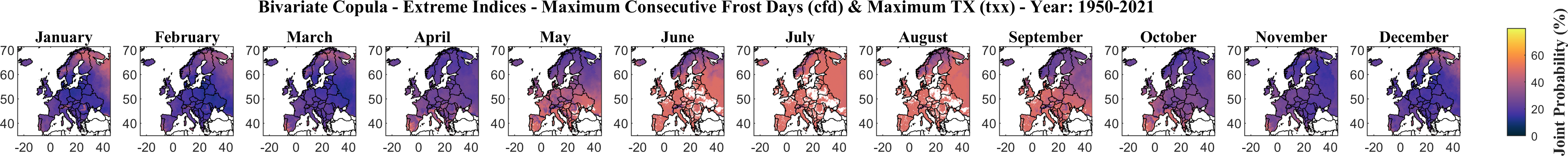

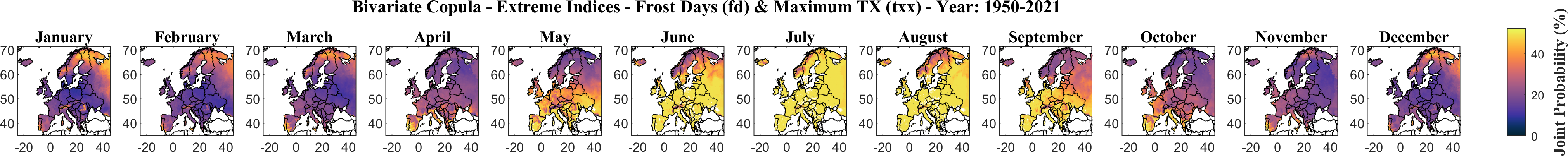

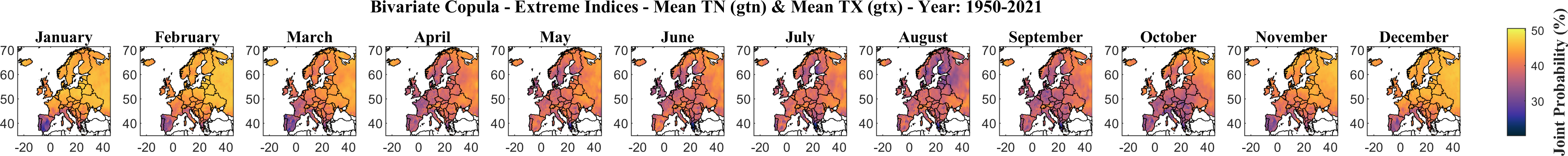

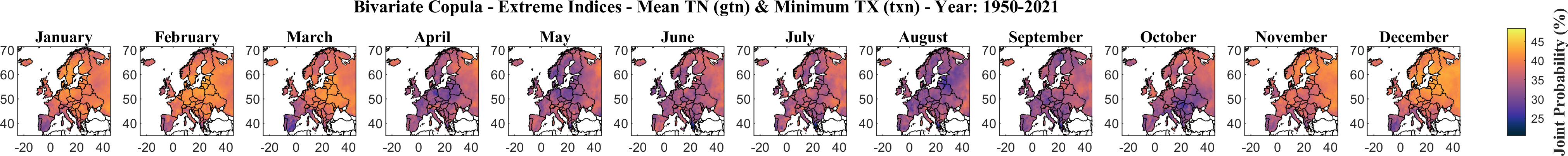

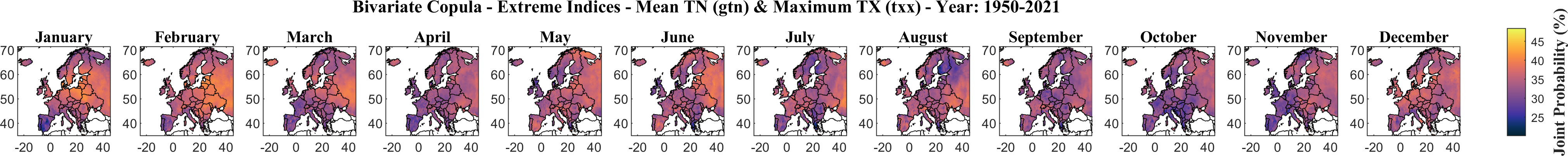

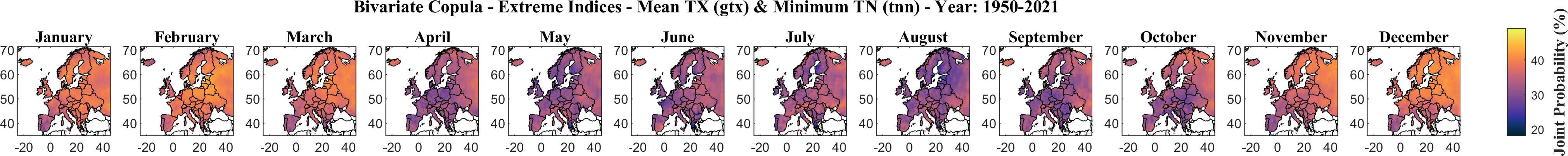

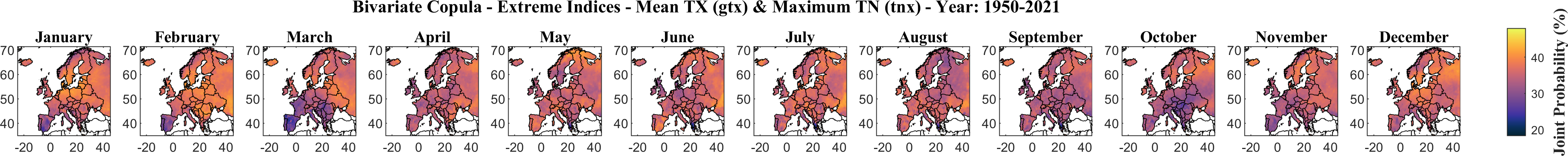

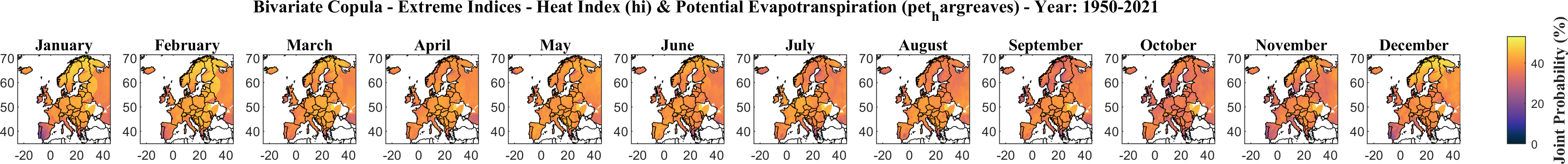

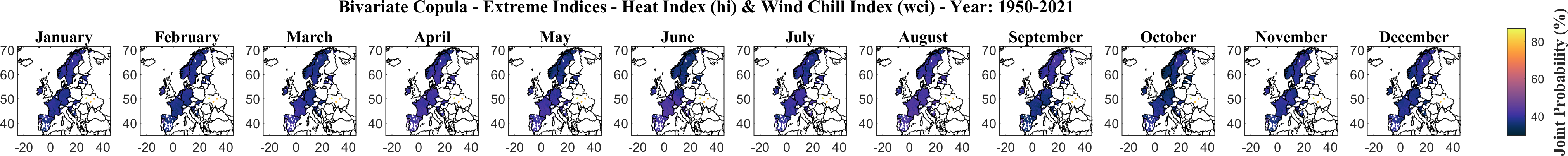

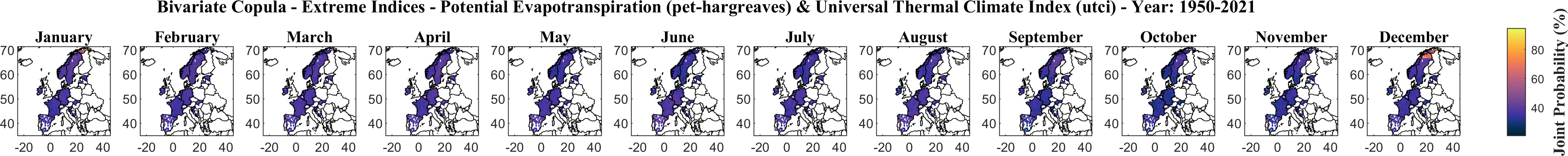

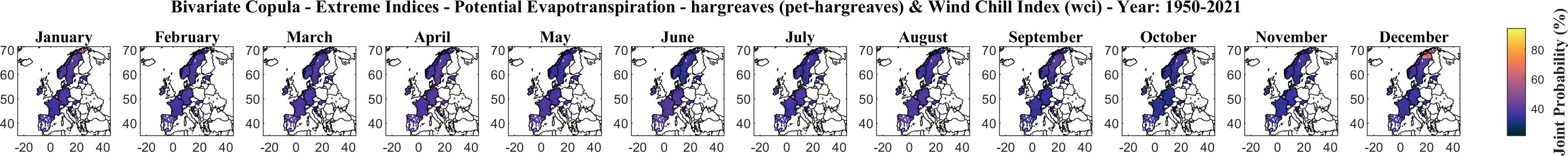

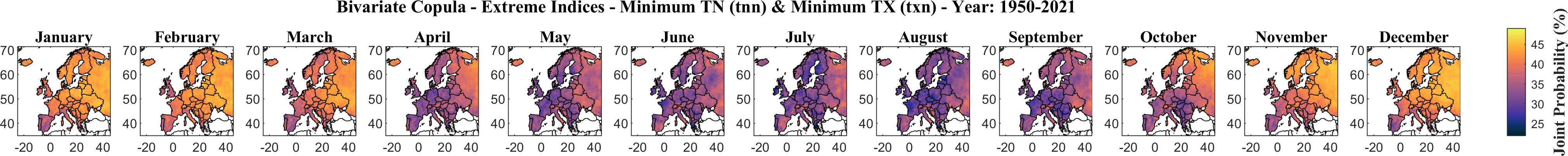

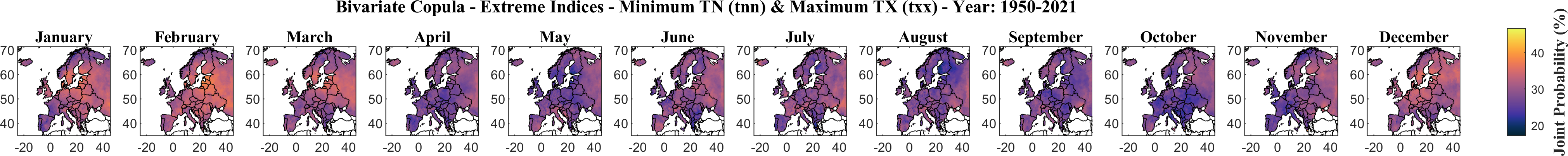

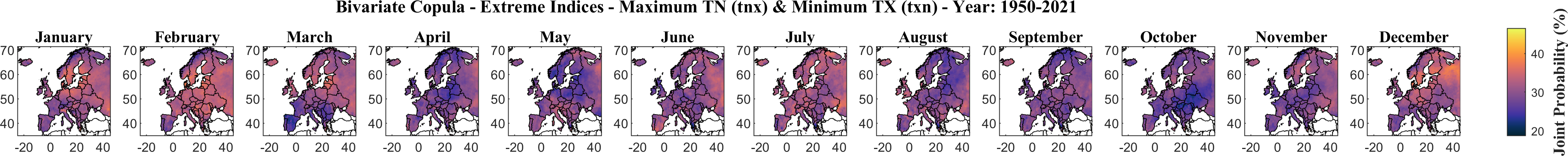

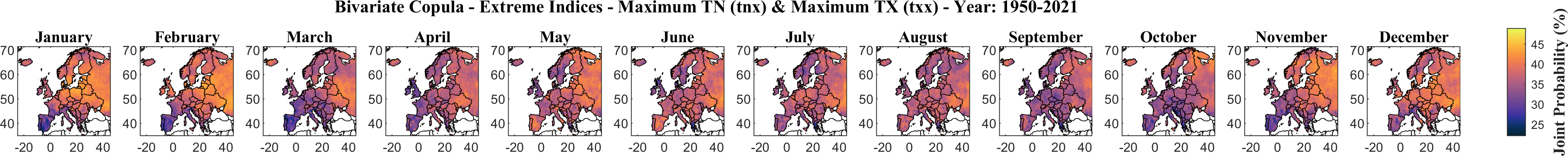


**B) Trivariate pairs**

**
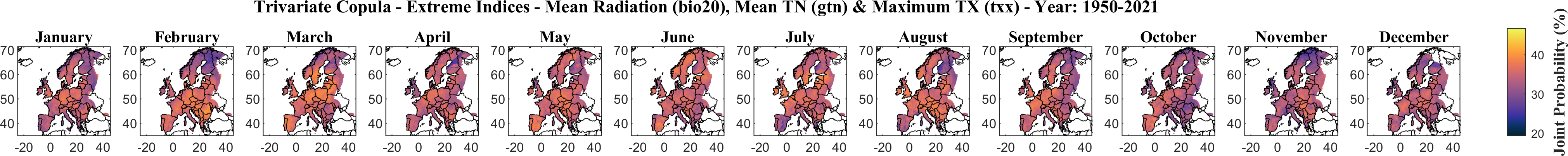

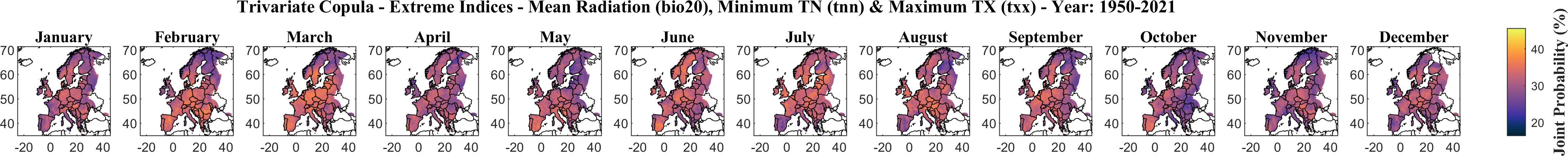

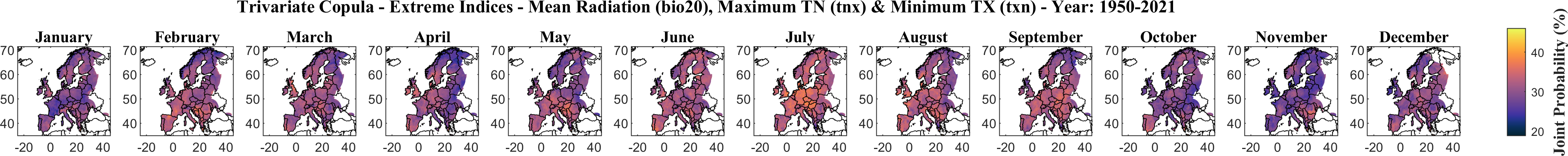

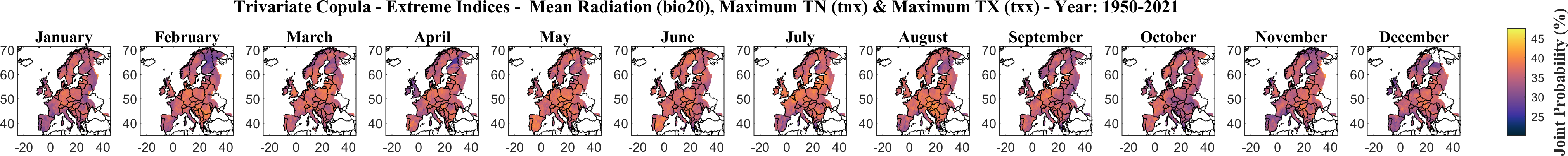

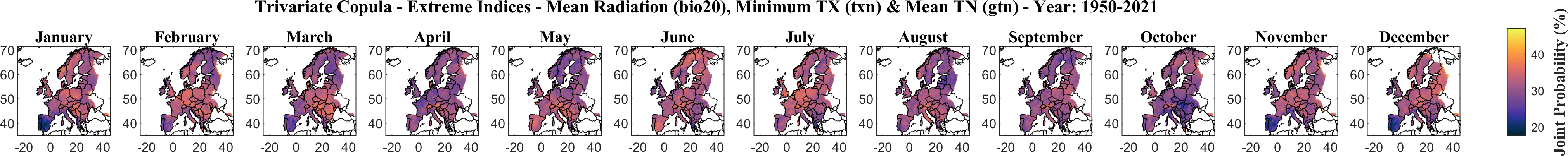

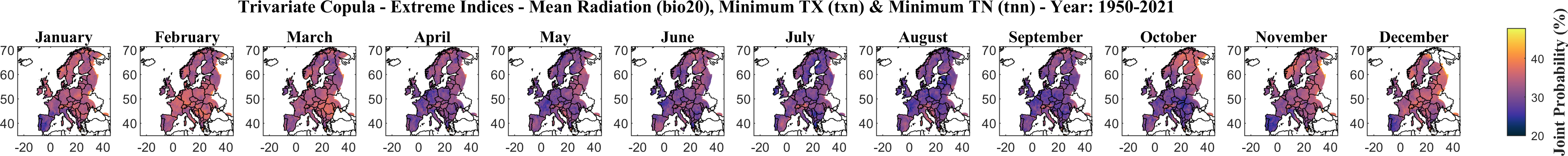

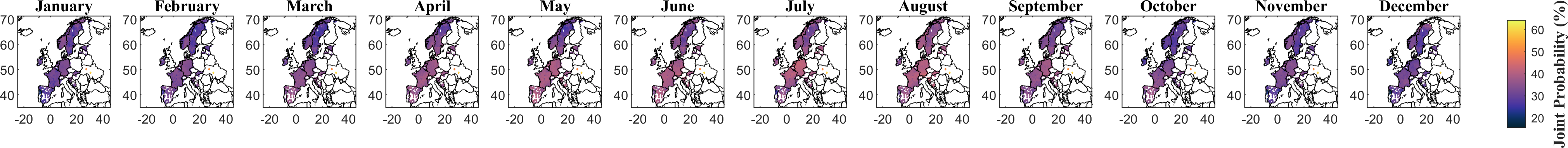

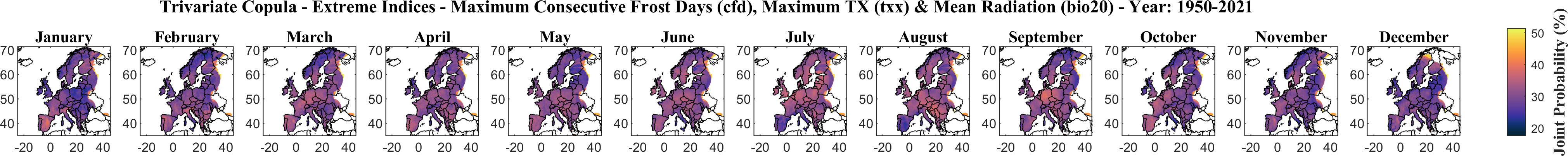

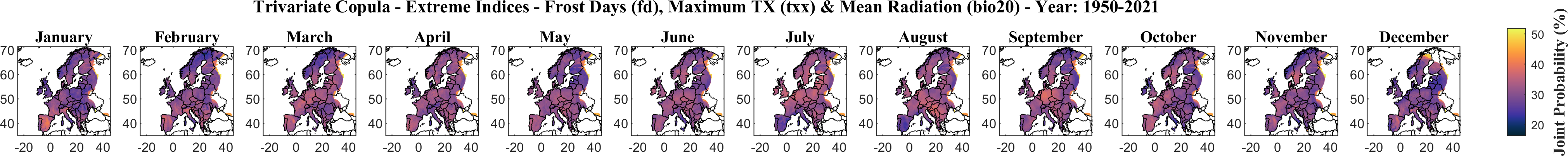

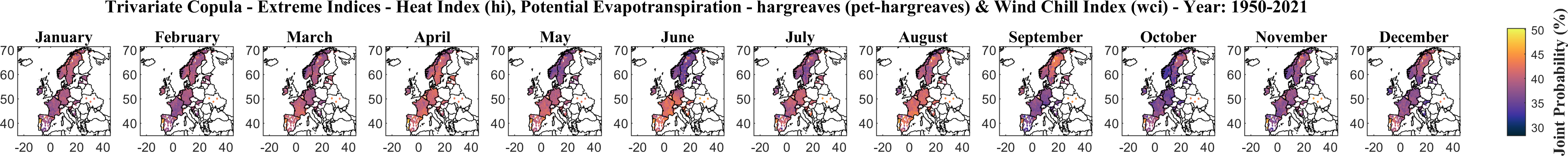
**

Figure S2: Risk maps of compound events of A) bivariate B) trivariate pairs obtained using best-fit copula approach. Risk maps depicts joint probability distribution of A) two B) three different climate indices. Yellow indicates the highest joint probability and dark blue indicates lowest joint probability of that compound event.
