# Supplementary material for: Hotspot movement of compound events on the Europe continent
Source: Sci Rep. 2023 Oct 23;13:18100. doi: 10.1038/s41598-023-45067-6 (PMC10593787; doi:10.1038/s41598-023-45067-6)
Supplement: Supplementary file 3 — Supplementary Figure S3. [file 41598_2023_45067_MOESM3_ESM.docx]

**Figure S3: Box plot for comparison of various copula methods**

**A) Bivariate Analysis**





**B) Trivariate Analysis**





Figure S3: Box plot comparing the performance of various copula methods used in the study to determine the joint probabilities for each grid A) Bivariate B) Trivariate. Minimum, maximum, quantiles, median and outliers could be visualized in each of the approach.
