# Supplementary material for: Hotspot movement of compound events on the Europe continent
Source: Sci Rep. 2023 Oct 23;13:18100. doi: 10.1038/s41598-023-45067-6 (PMC10593787; doi:10.1038/s41598-023-45067-6)
Supplement: Supplementary file 4 — Supplementary Figure S4. [file 41598_2023_45067_MOESM4_ESM.docx]

**Figure S4: Spatial trend analysis using Mann-Kendall and Sen’s slope for compound events**

**A) Mann-Kendall test - bivariate pairs**


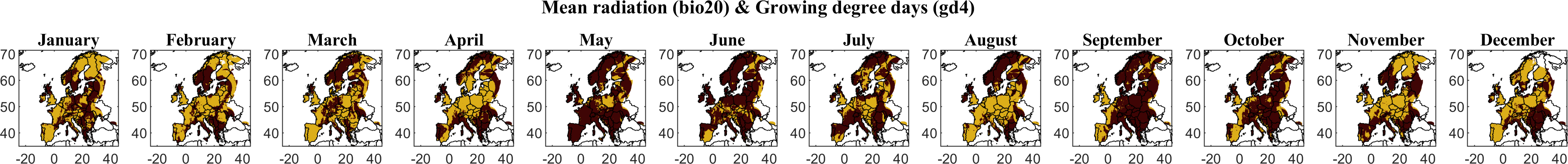

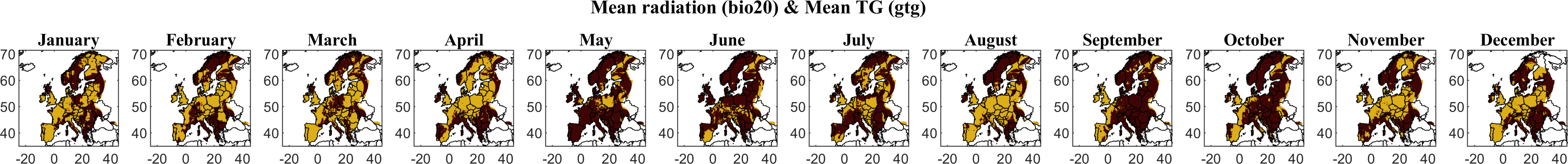

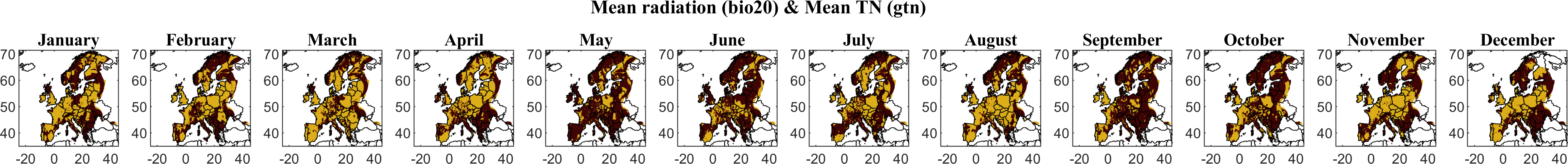

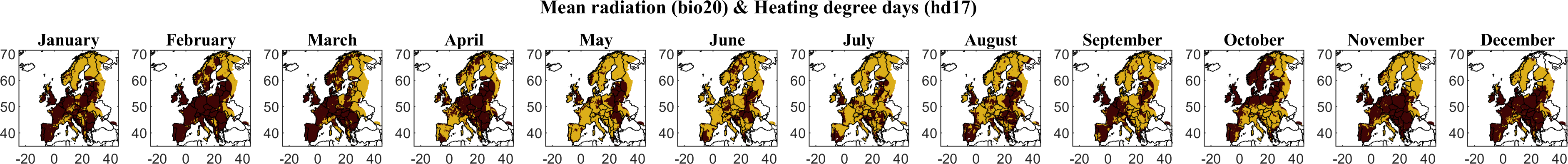

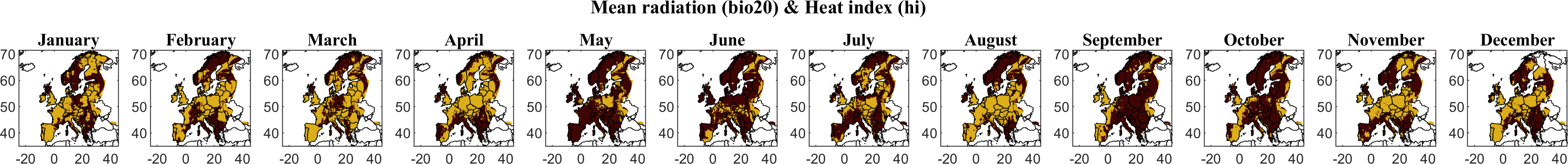

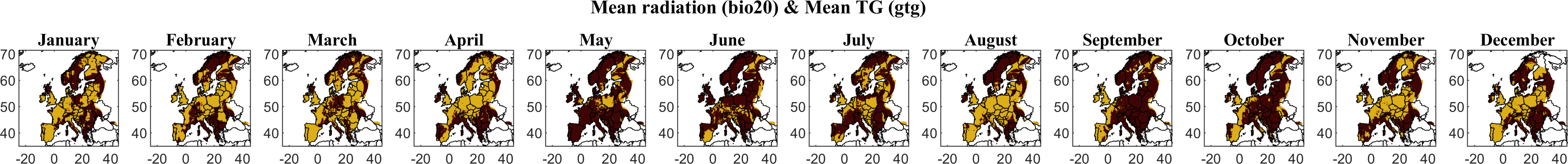

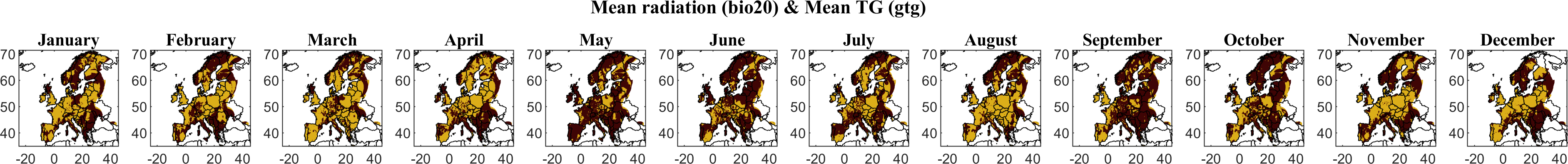

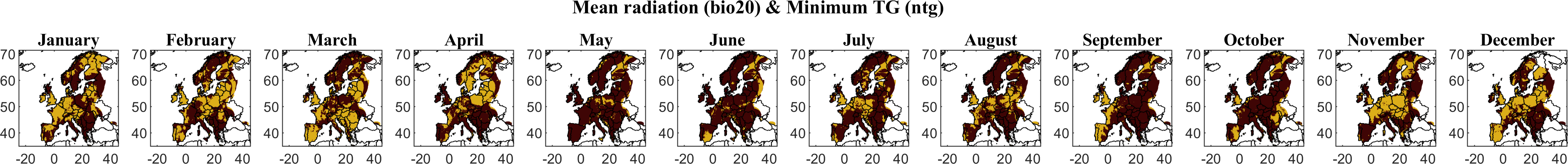

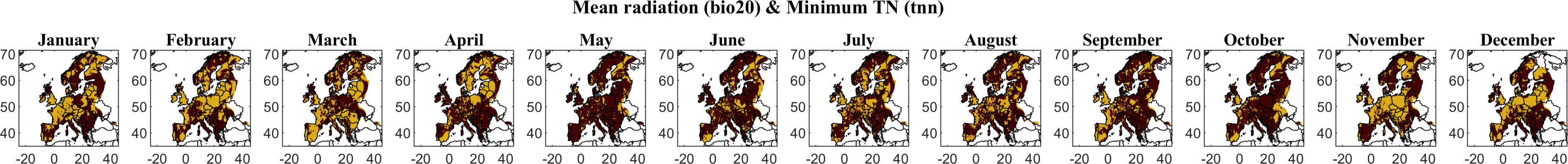

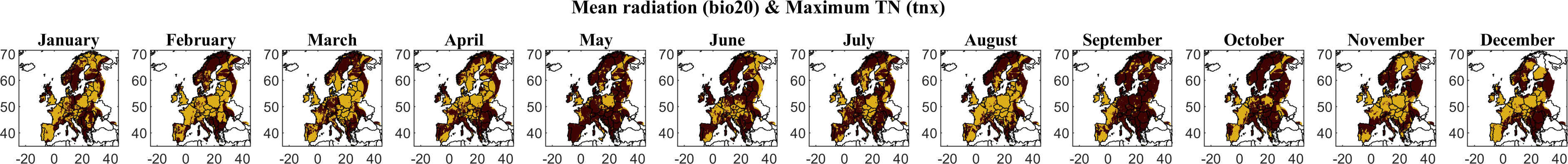

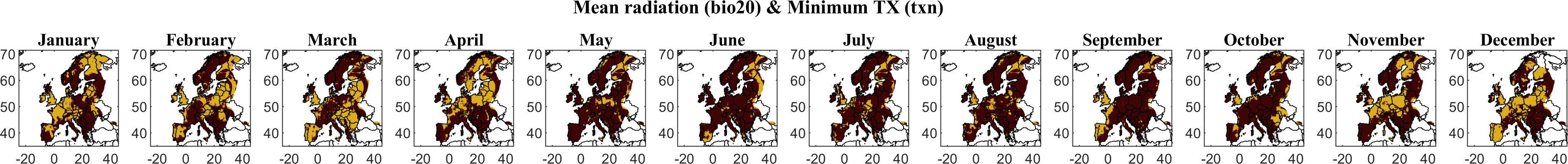

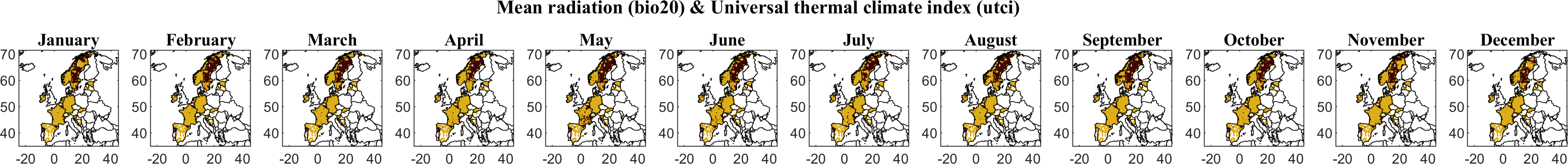

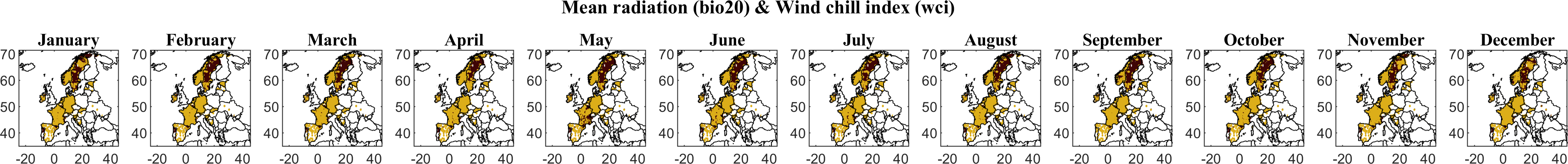

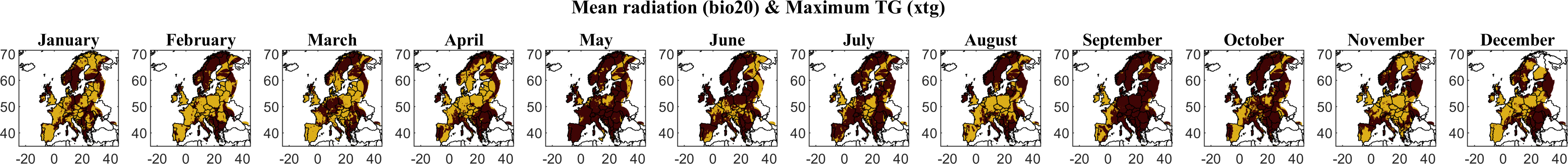

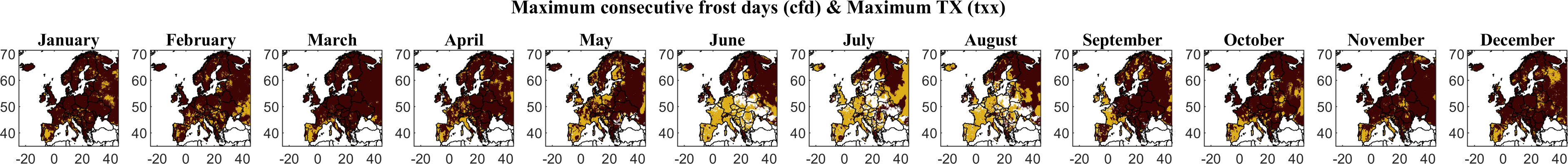

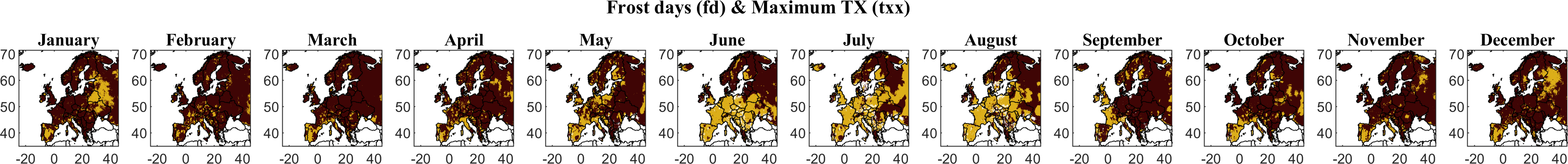

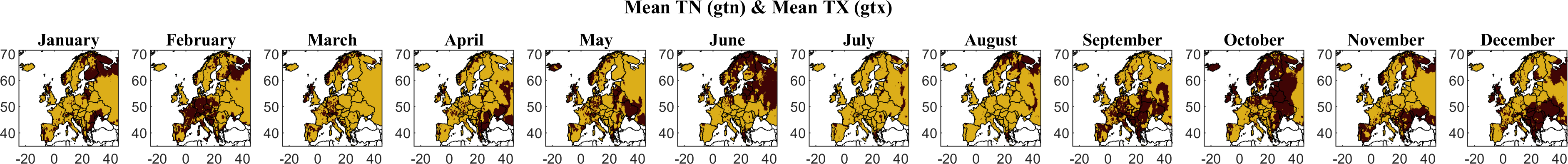

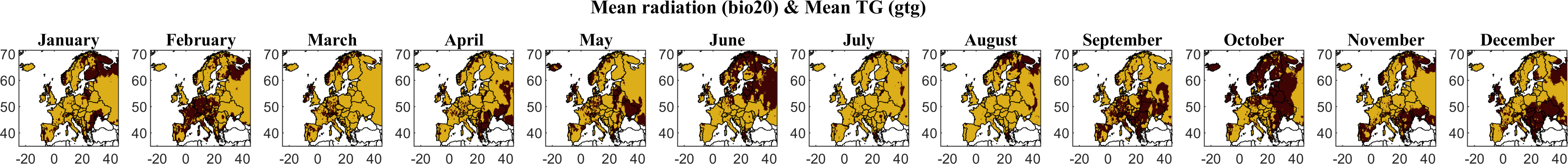

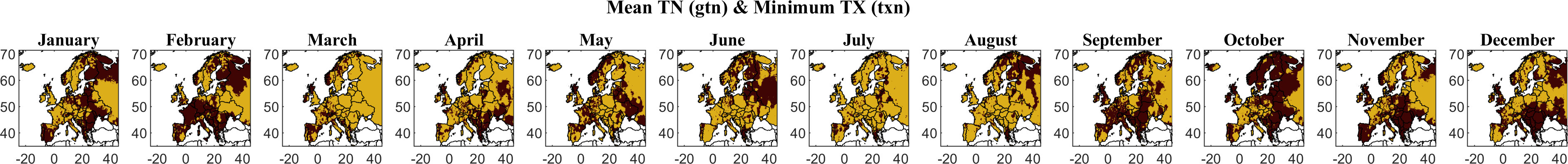

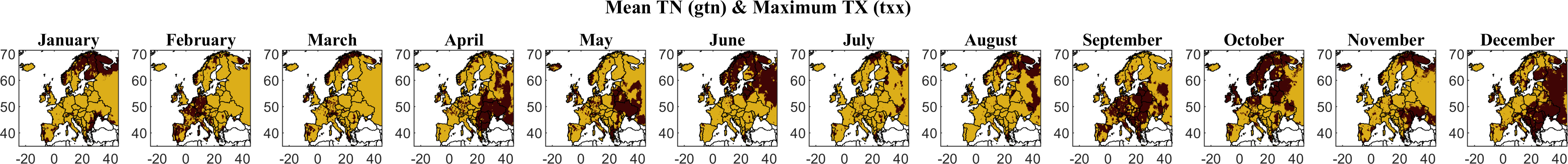

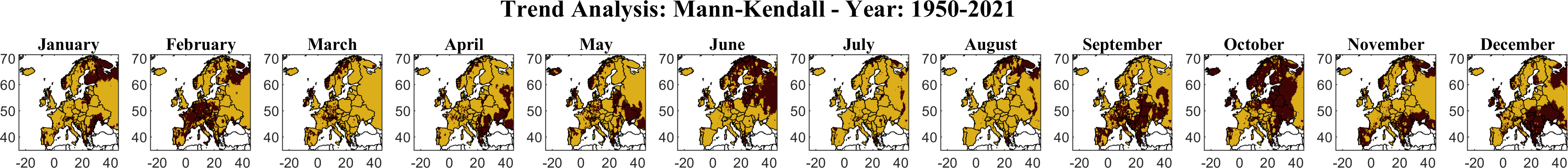

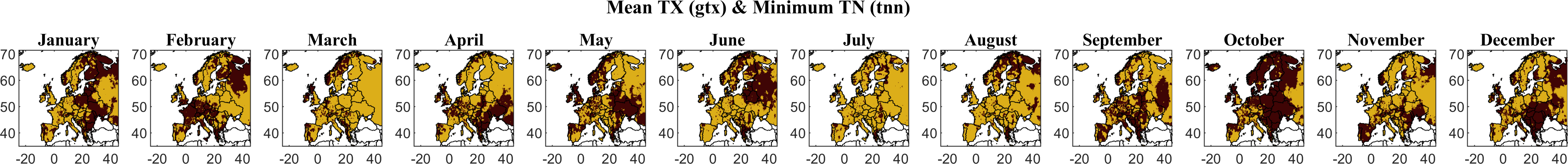

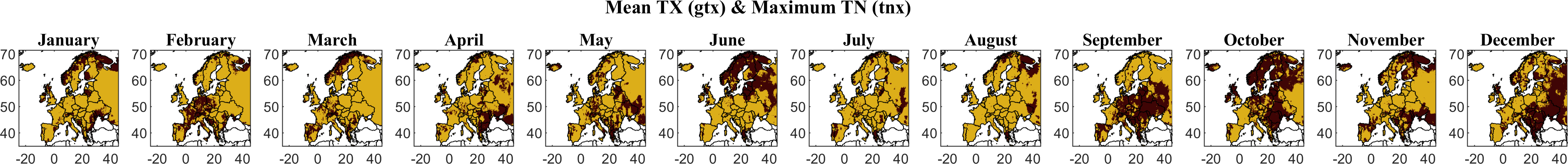

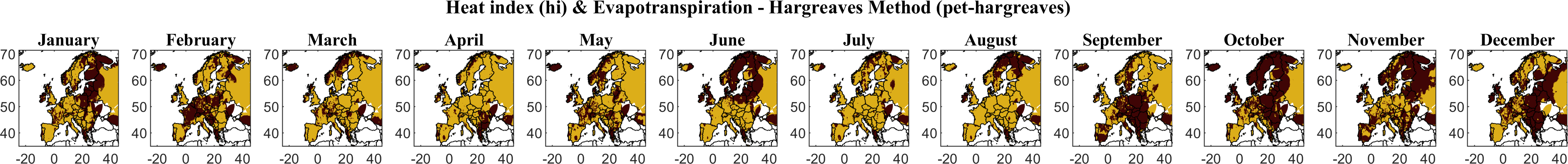

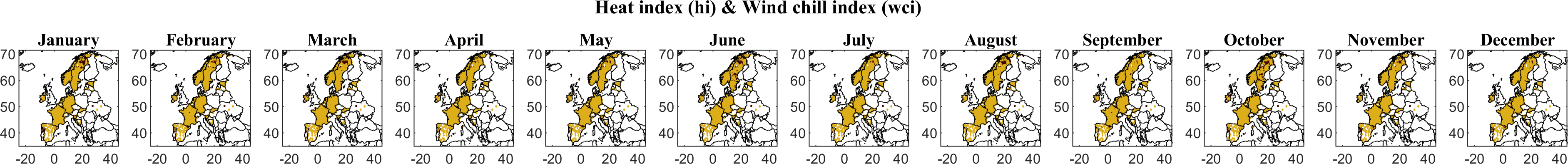

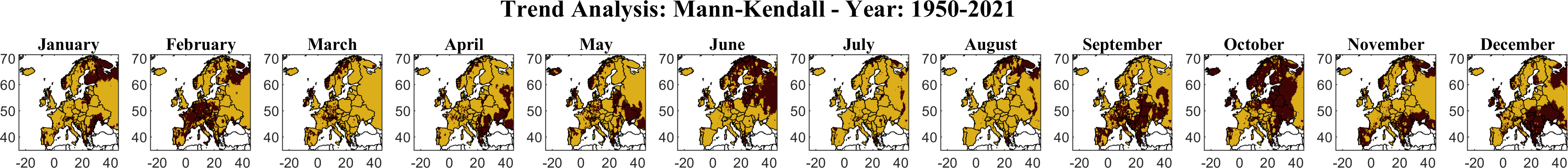

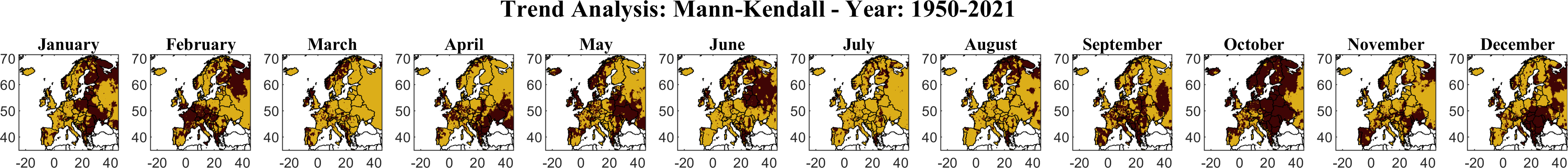

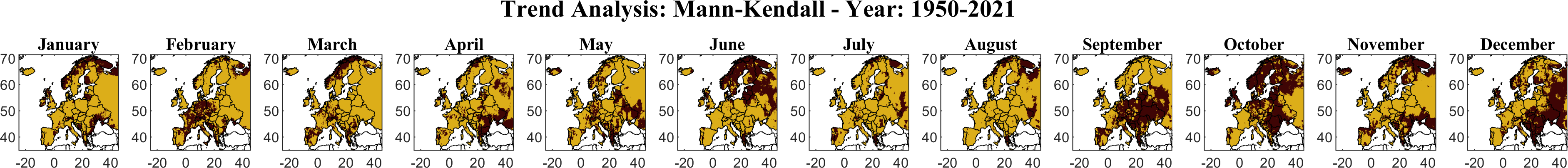

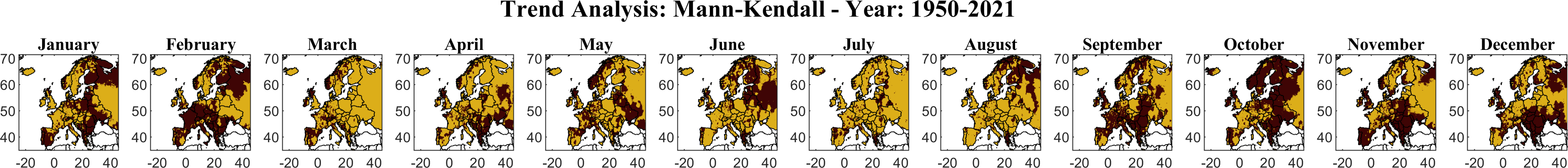

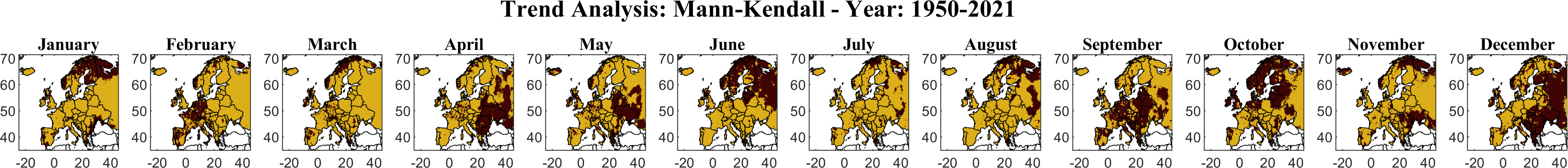

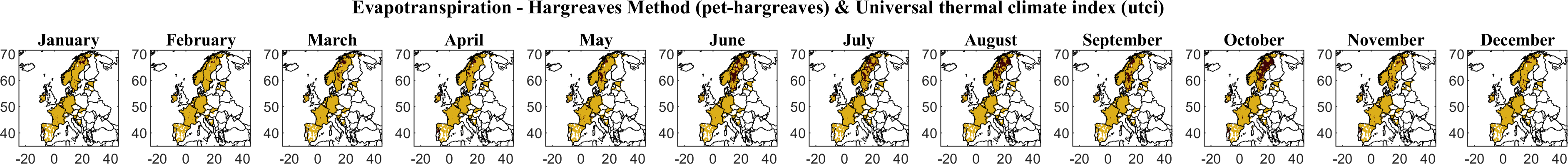

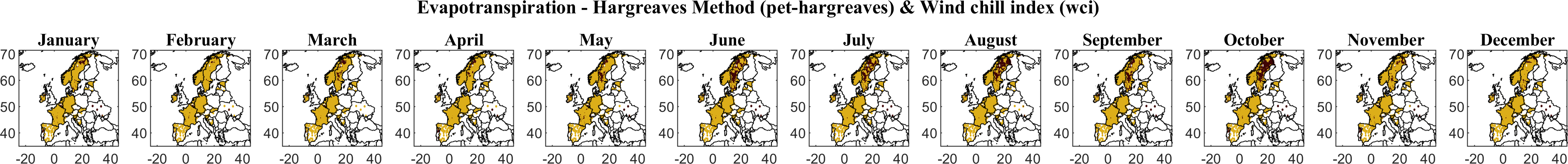

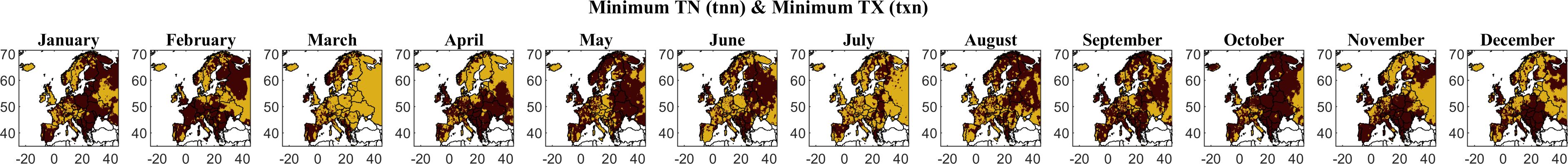

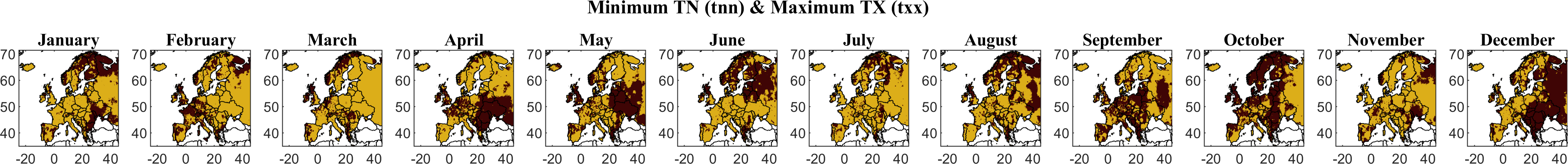

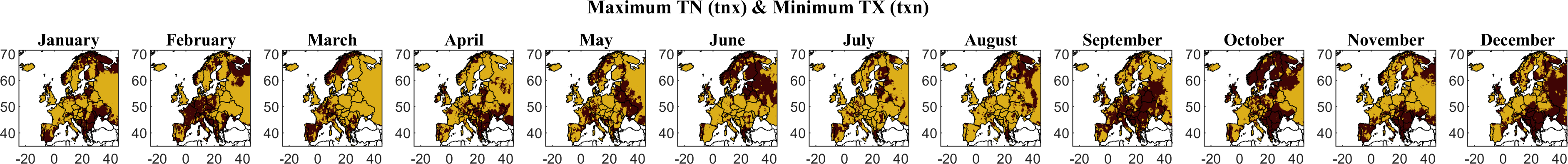

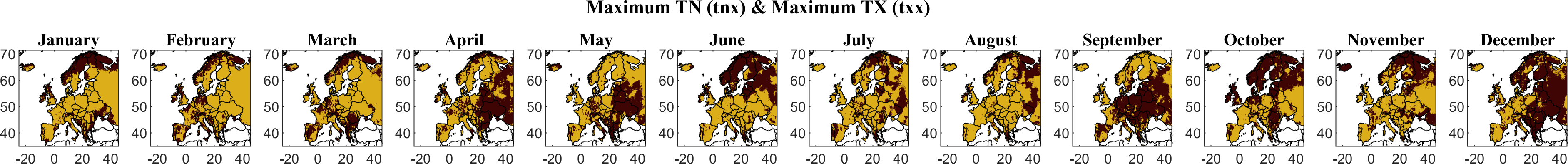

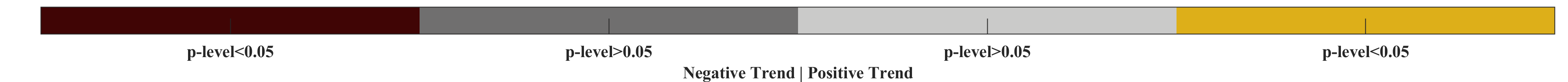
 **B) Sen’s slope estimator - bivariate pairs**

**
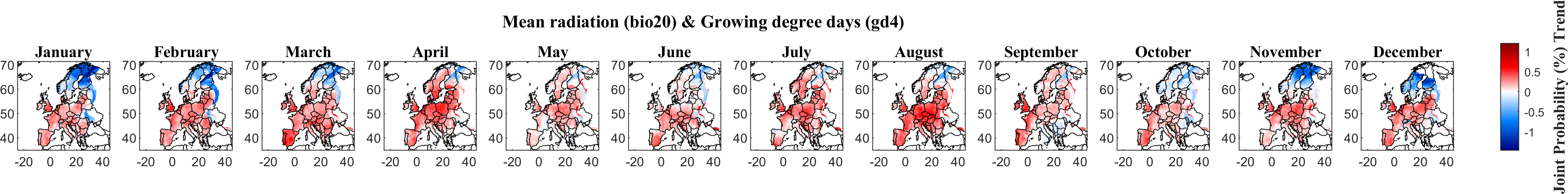

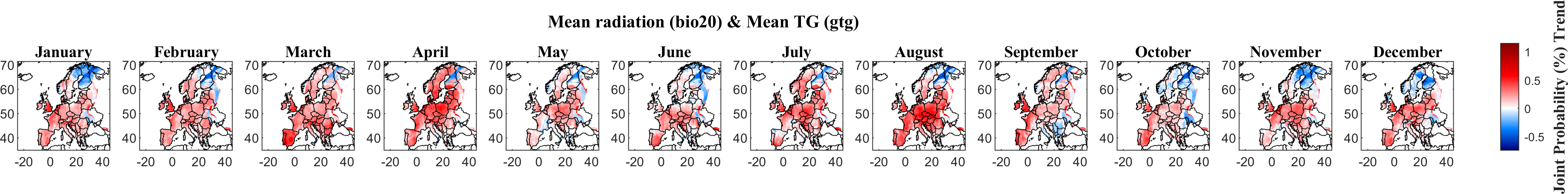

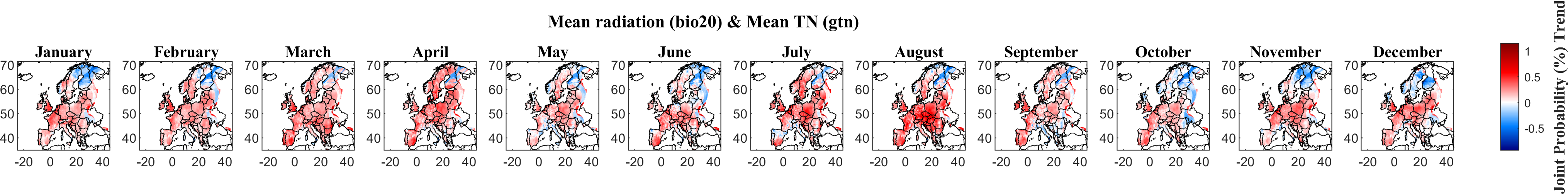

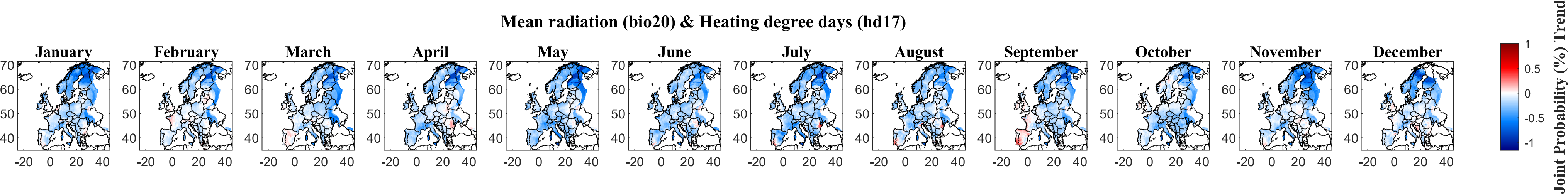

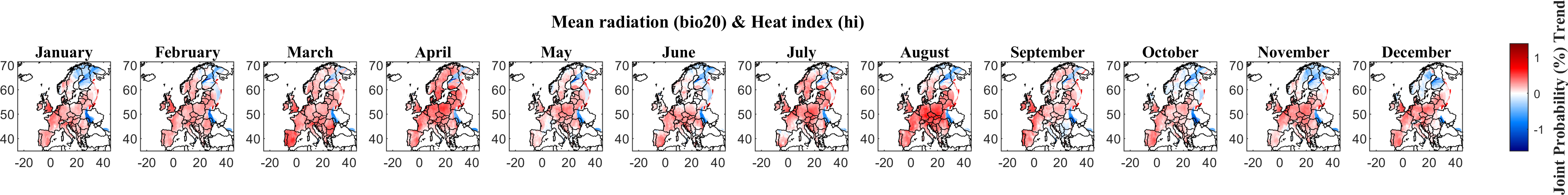

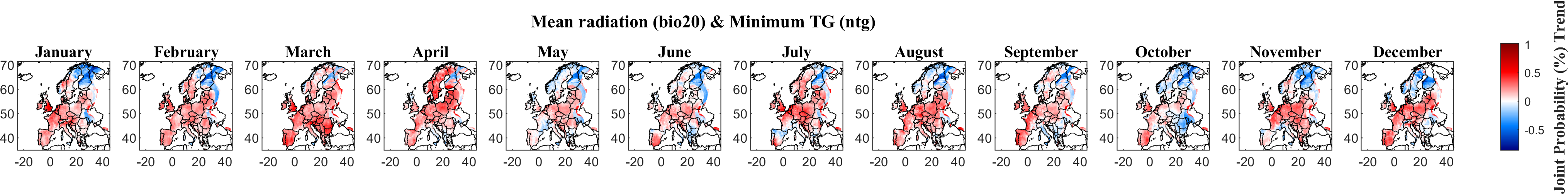

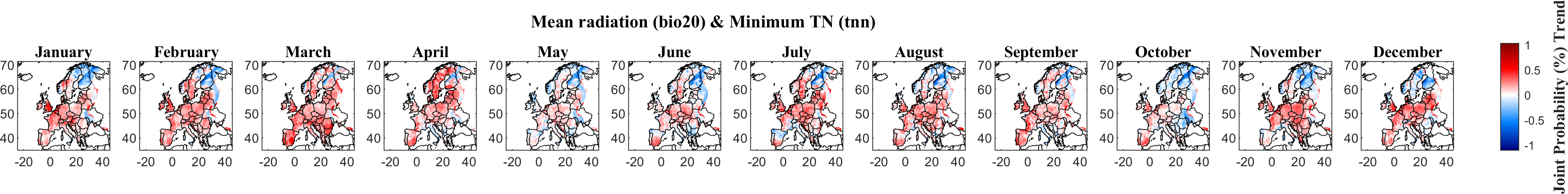

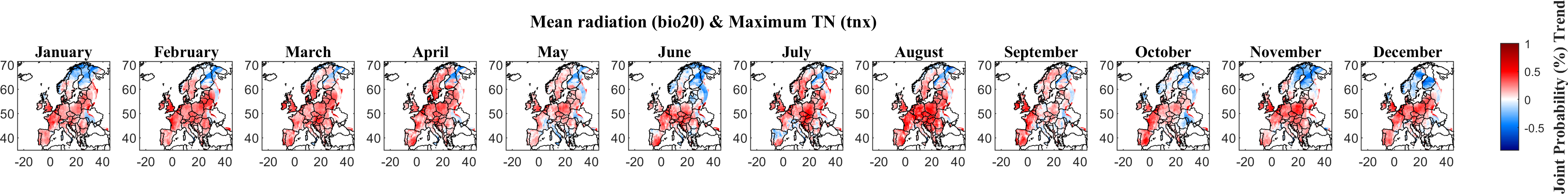

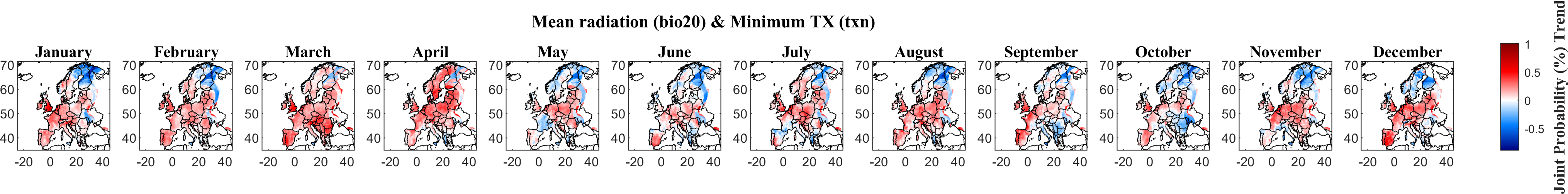

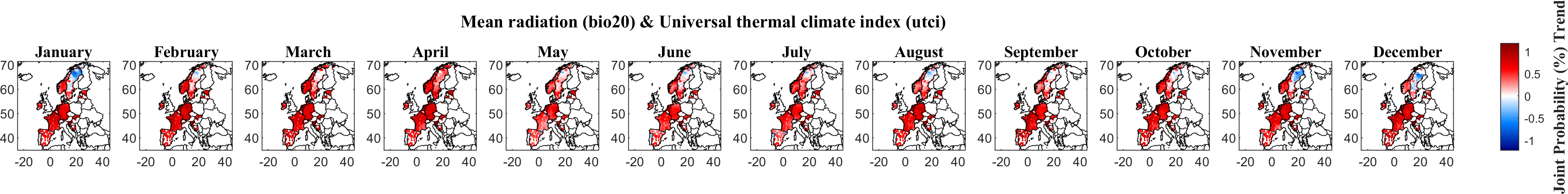

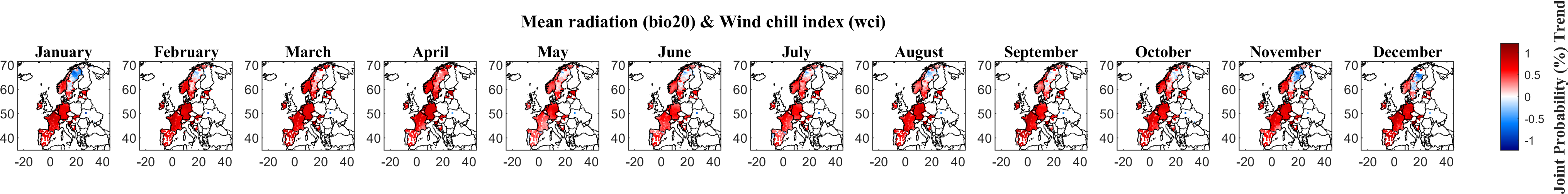

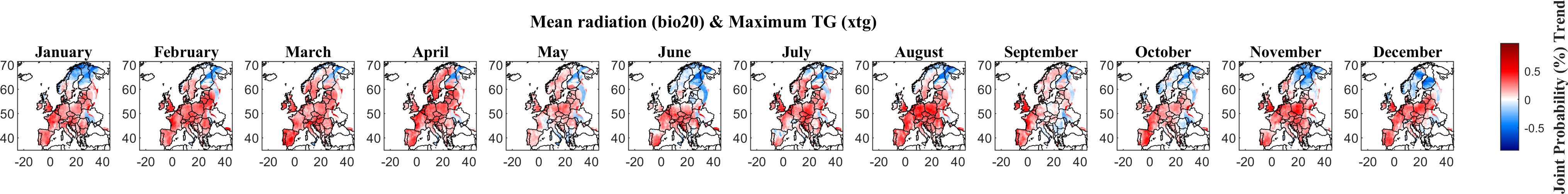

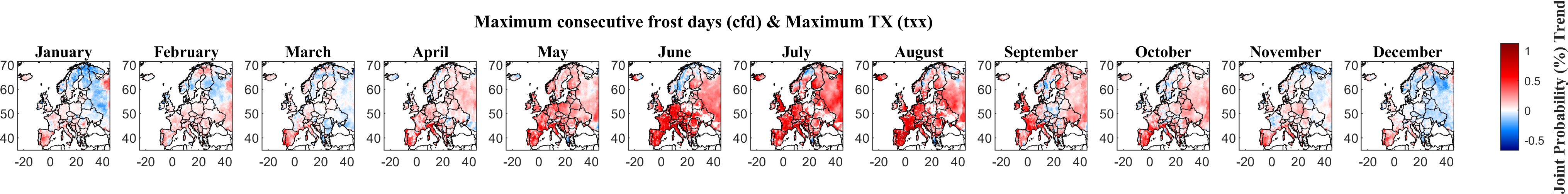

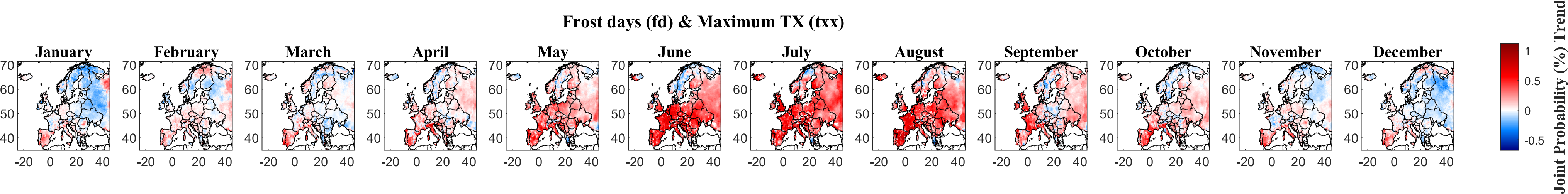

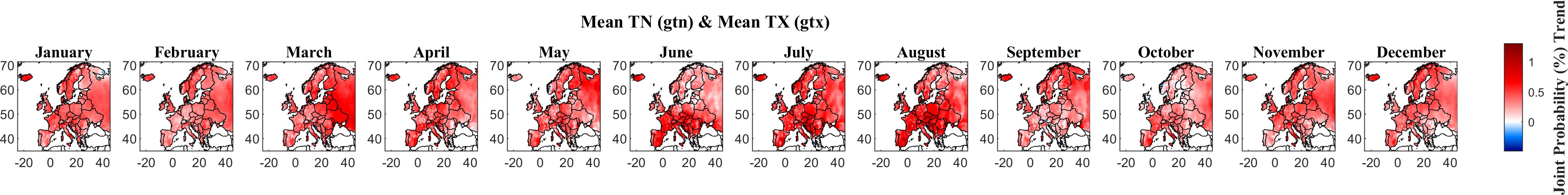

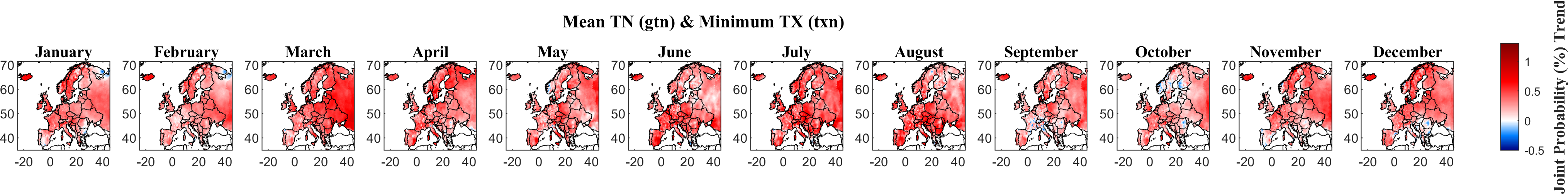

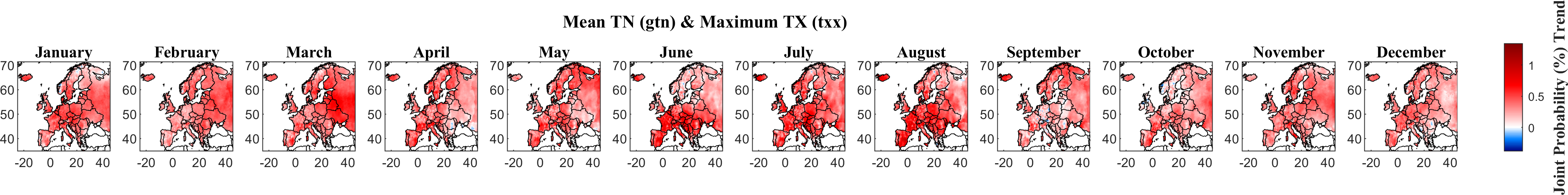

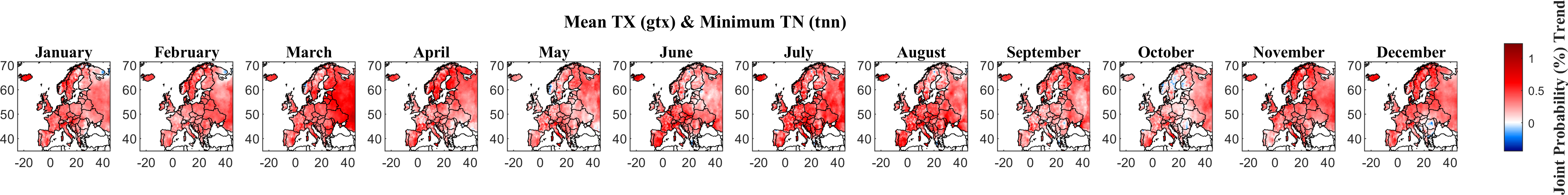

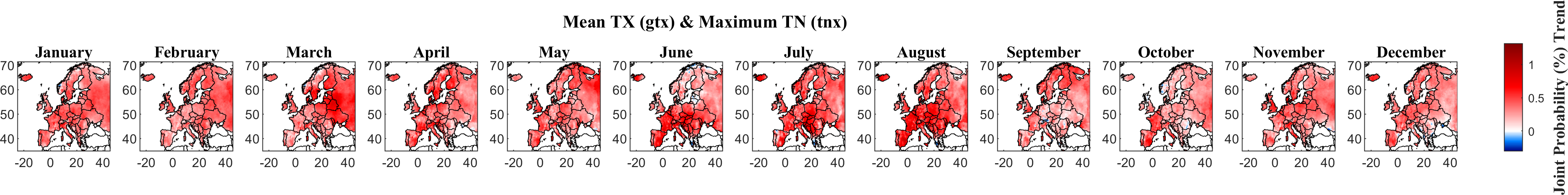

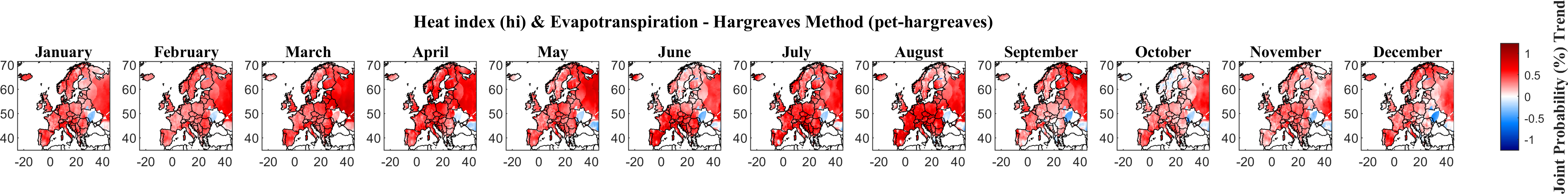

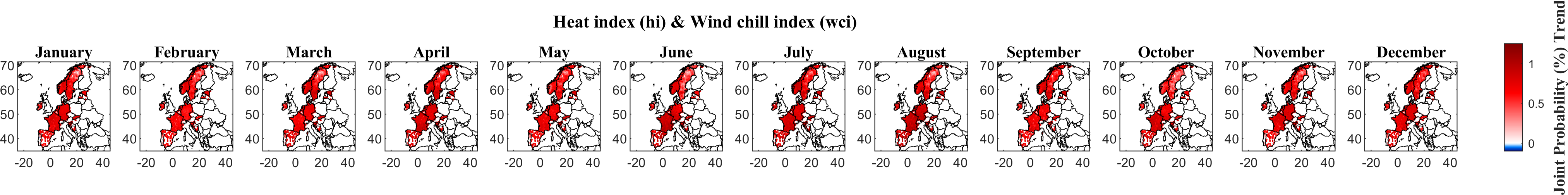

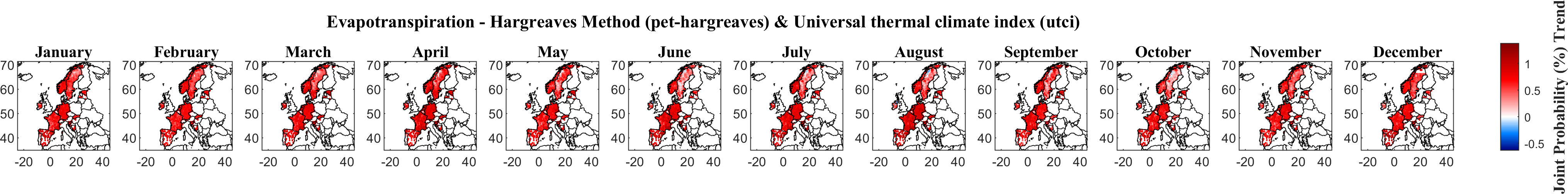

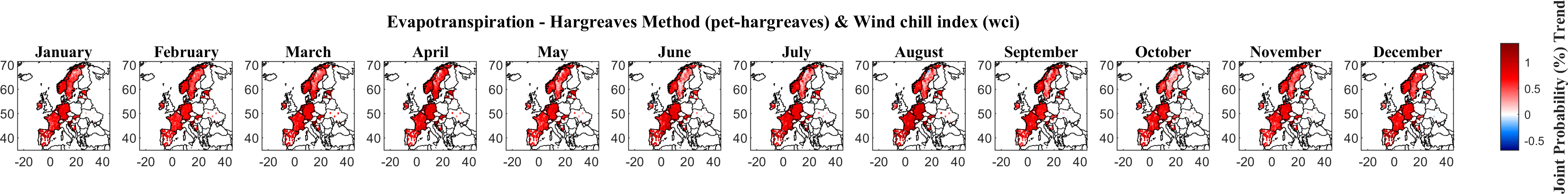

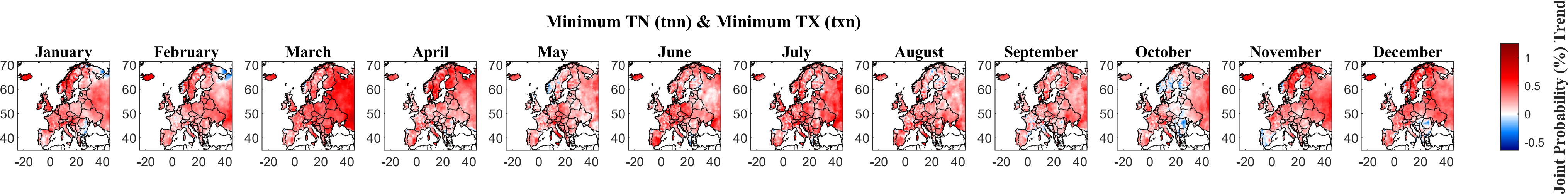

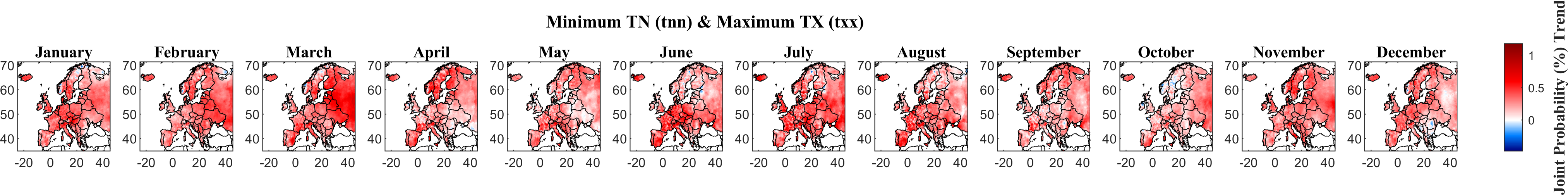

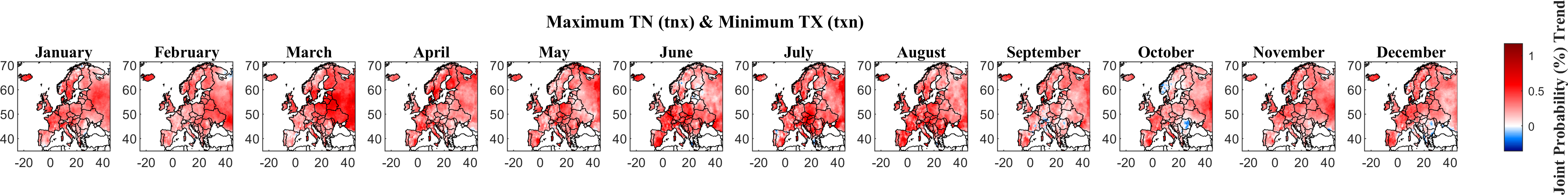

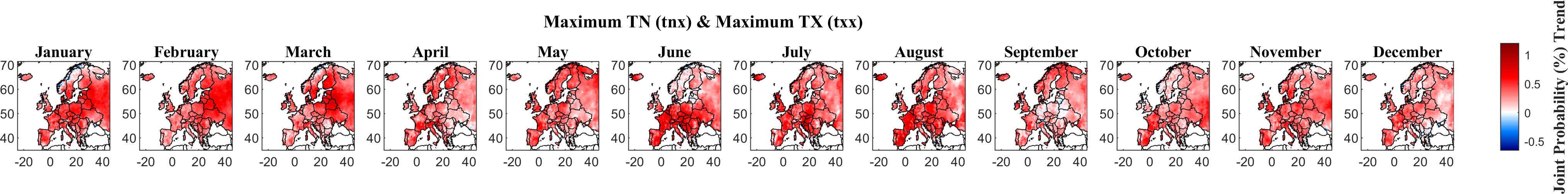
**

**C) Mann-Kendall test - trivariate pairs**

**
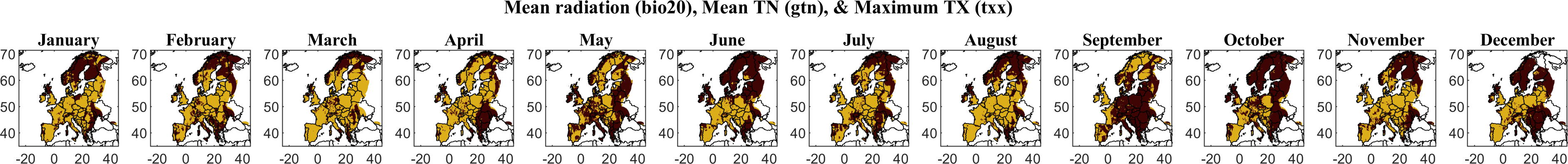

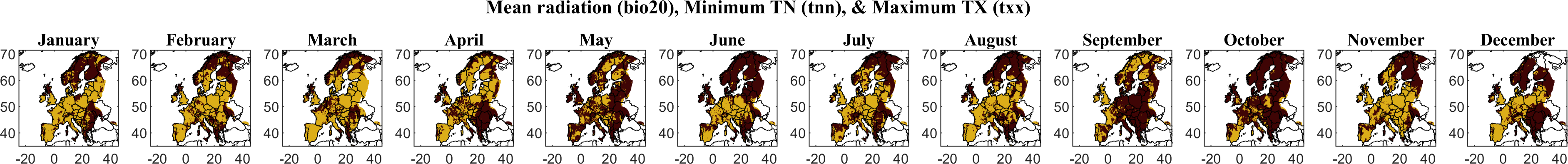

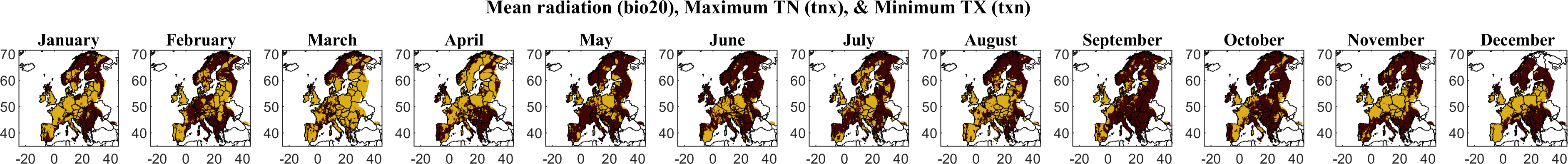

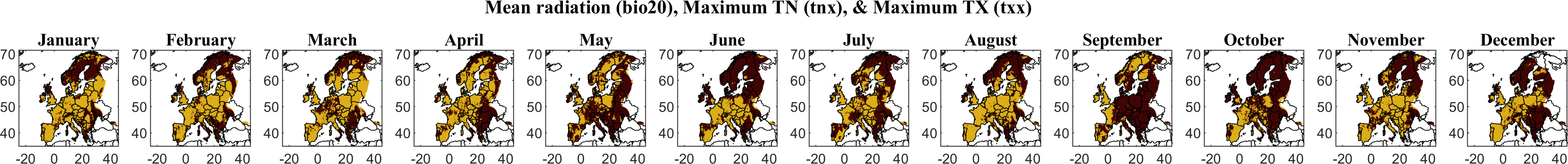

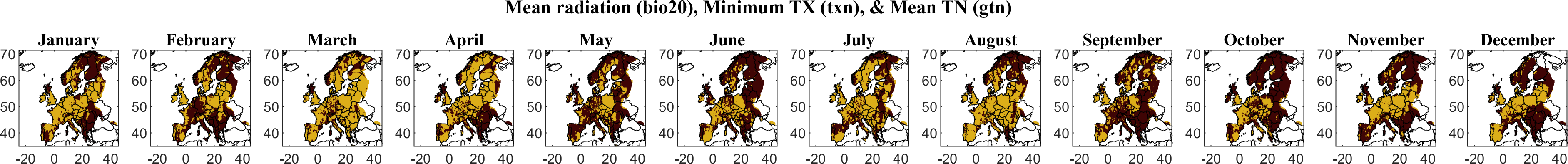

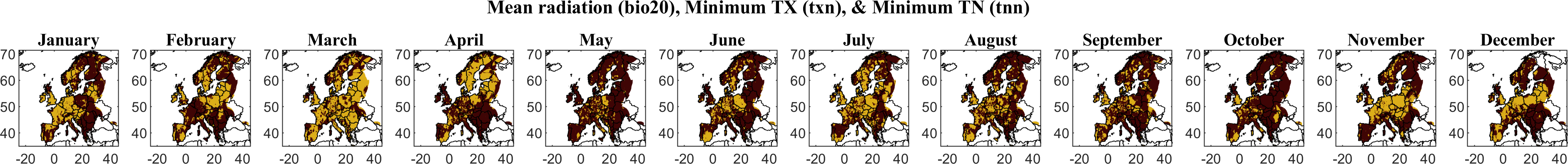

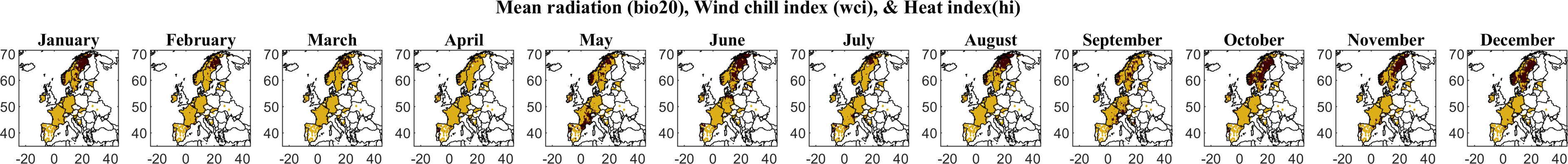

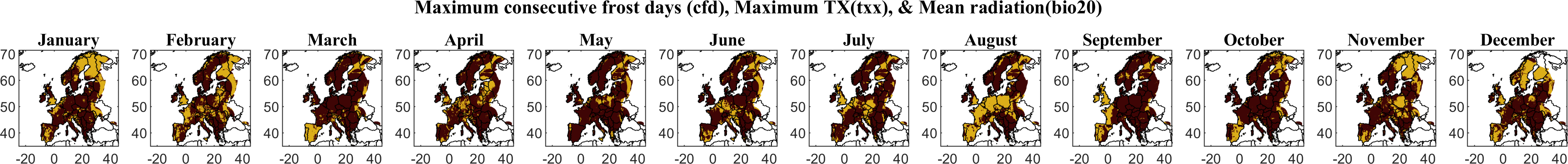

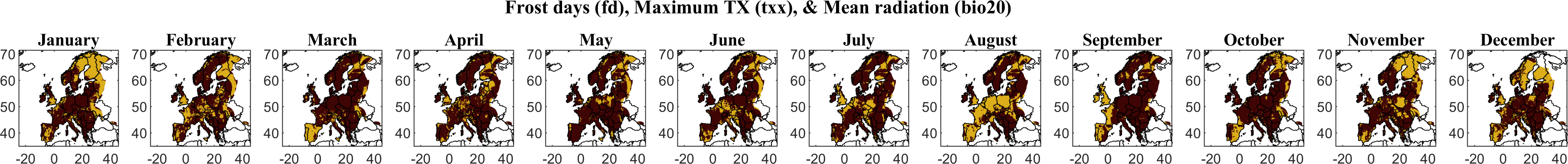

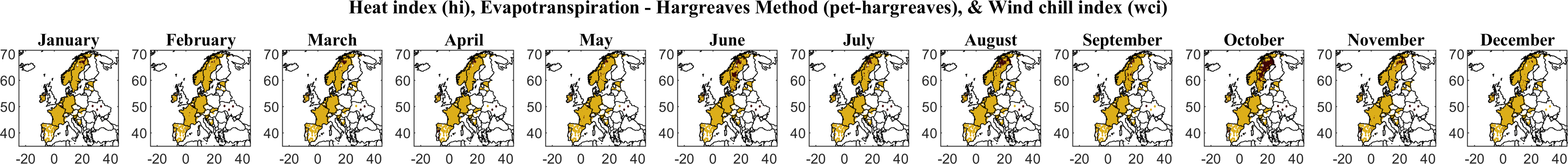
**


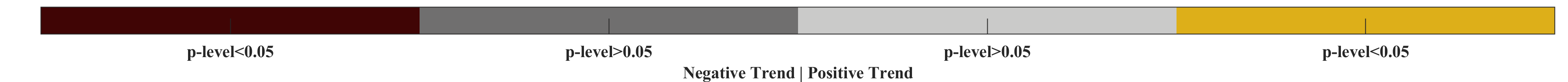


**D) Sen’s slope estimator - trivariate pairs**

**
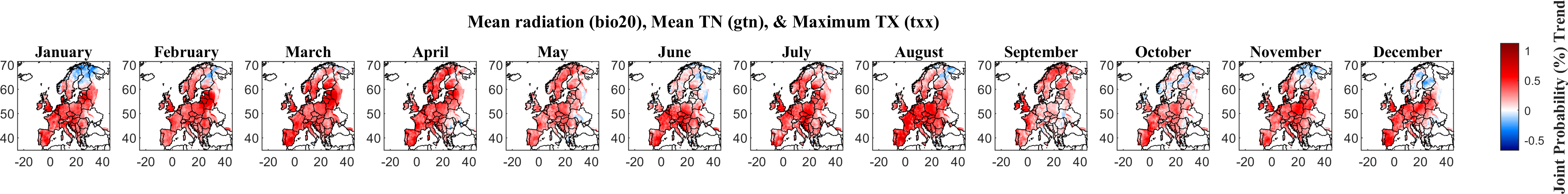
**

**
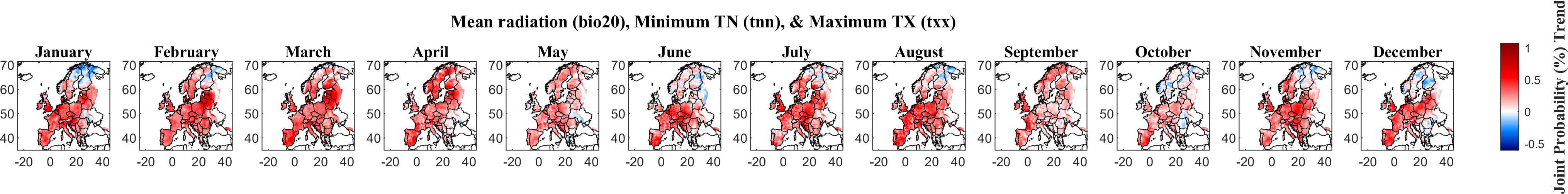

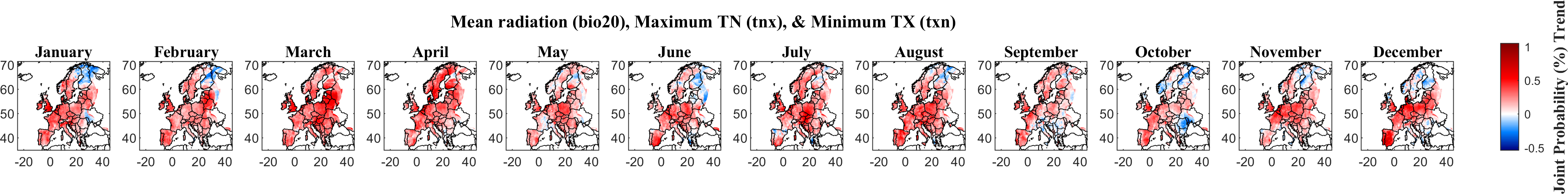

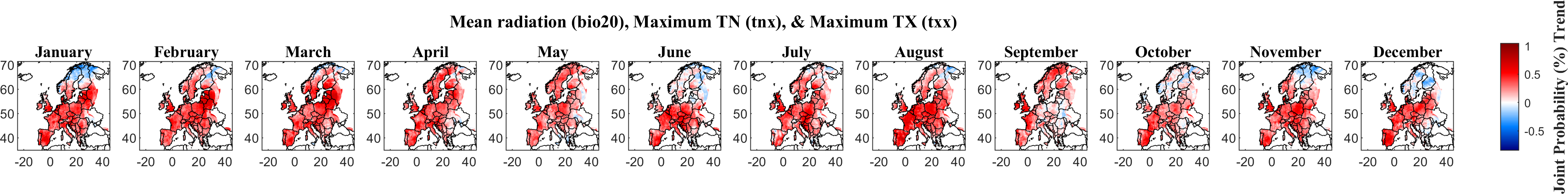

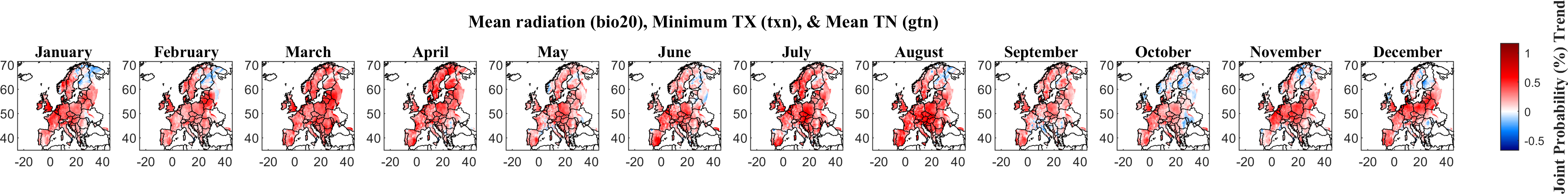

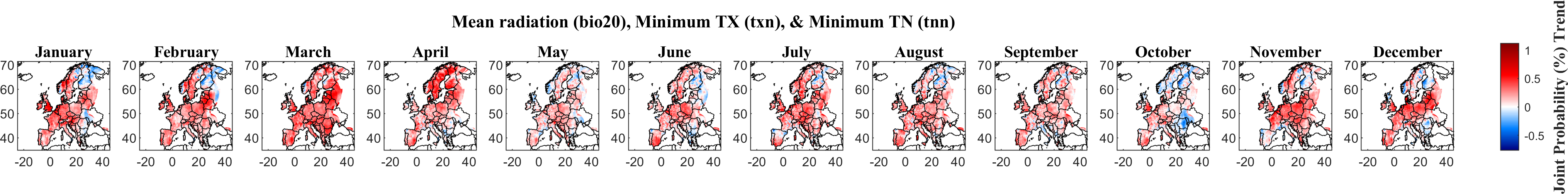

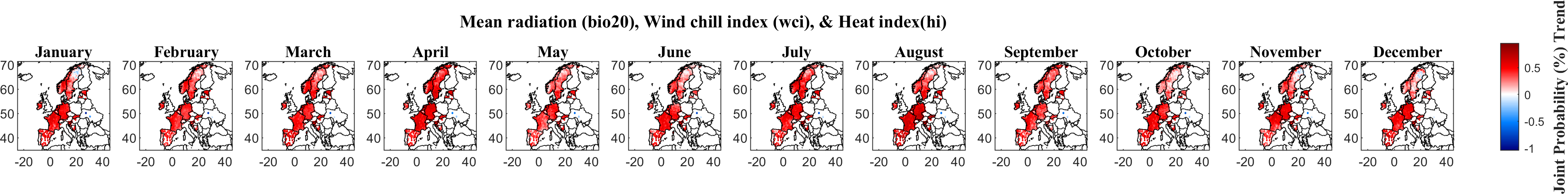

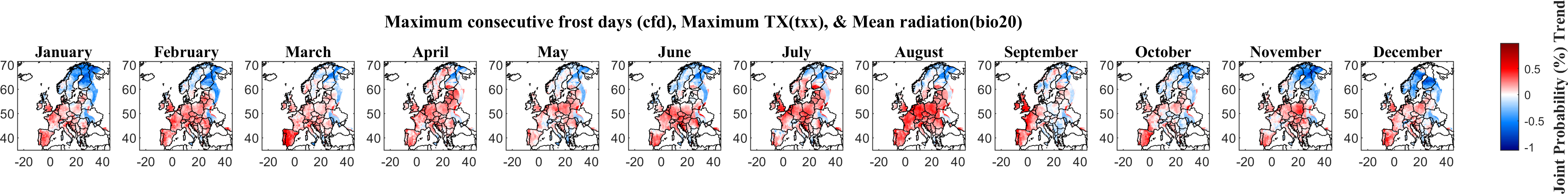

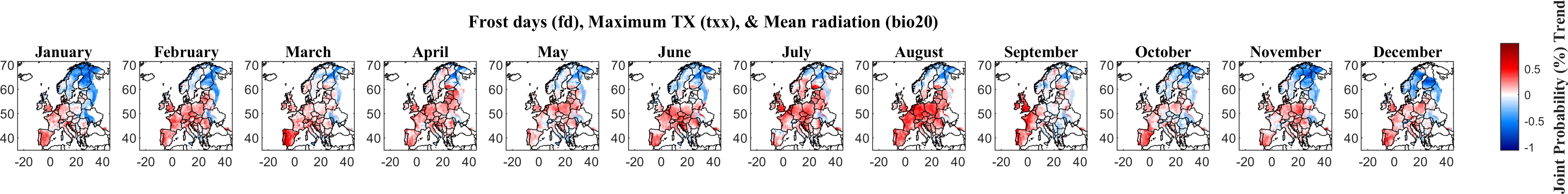

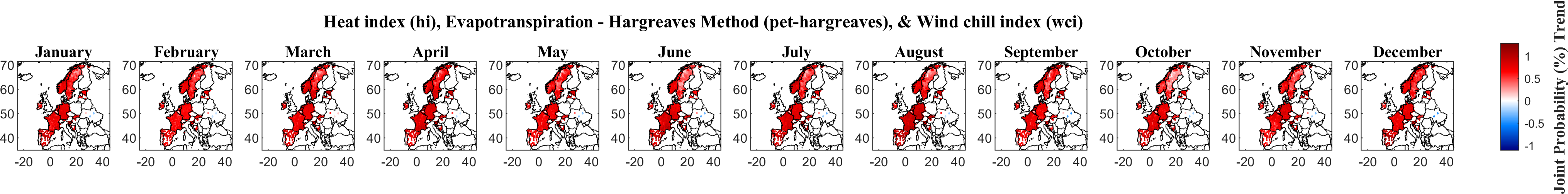
**

Figure S4: Mann-Kendall and Sen’s slope for A)/B) Bivariate and C)/D) Trivariate: spatial distribution across Europe. Direction of trend from Mann-Kendall with significance level <0.05 (Dark yellow for positive and Maroon for negative trend). Magnitude of change from Sen’s slope estimator (Red increasing percentage and blue is decreasing percentage of joint probability; white there is no change in the joint probability). Positive trend and increasing magnitude of change indicates European regions, which are more likely to be in risk for future.
