# Supplementary material for: Hotspot movement of compound events on the Europe continent
Source: Sci Rep. 2023 Oct 23;13:18100. doi: 10.1038/s41598-023-45067-6 (PMC10593787; doi:10.1038/s41598-023-45067-6)
Supplement: Supplementary file 5 — Supplementary Figure S5. [file 41598_2023_45067_MOESM5_ESM.docx]

**Figure S5: Monthly spatial distribution of climate indices – 1950-2021**

**
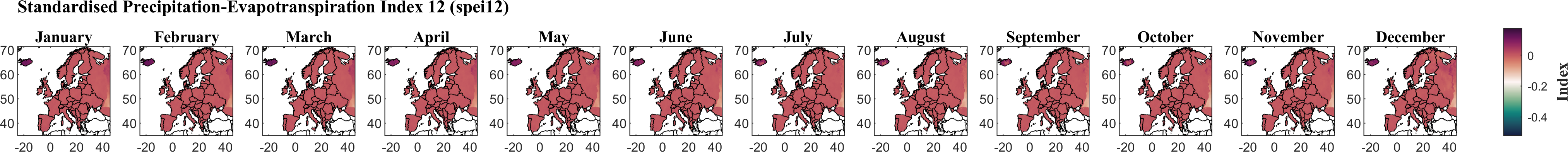

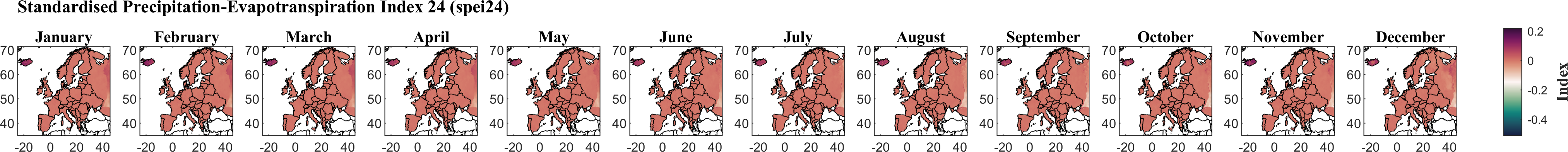

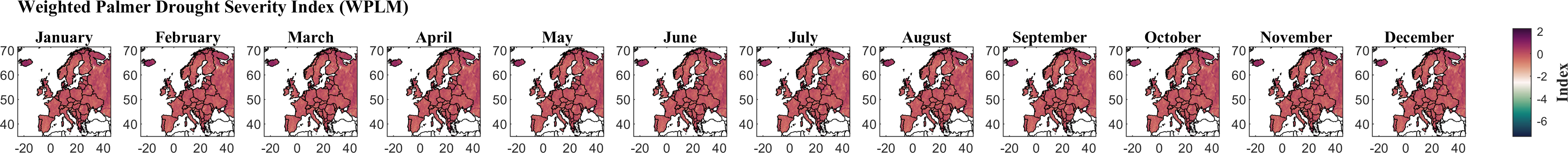
**


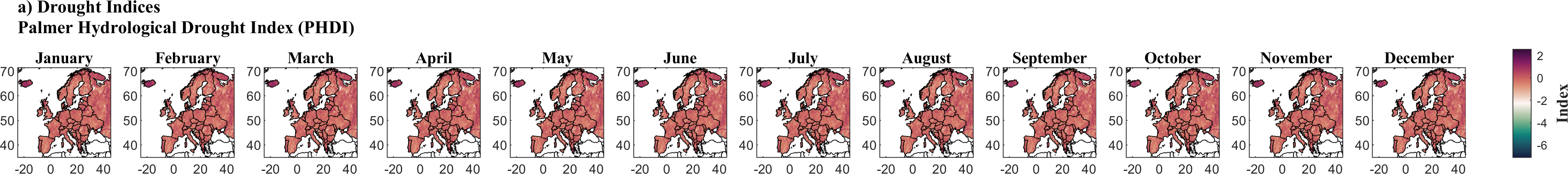

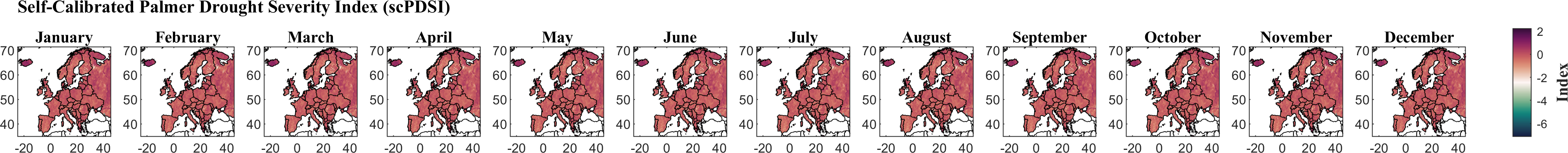

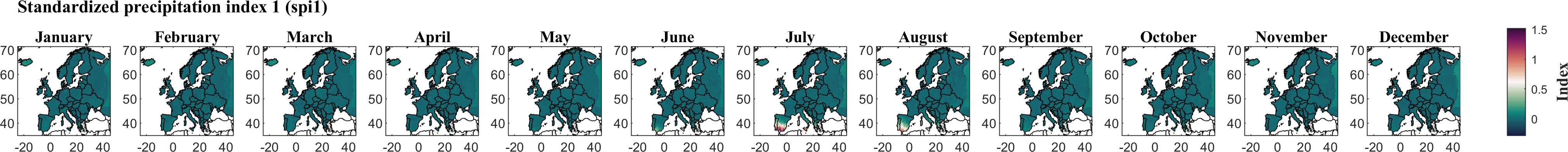

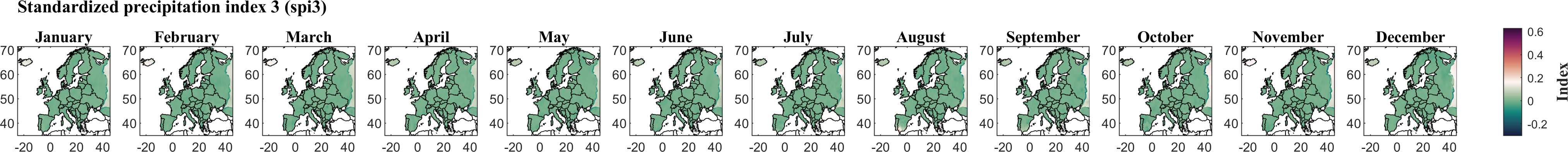

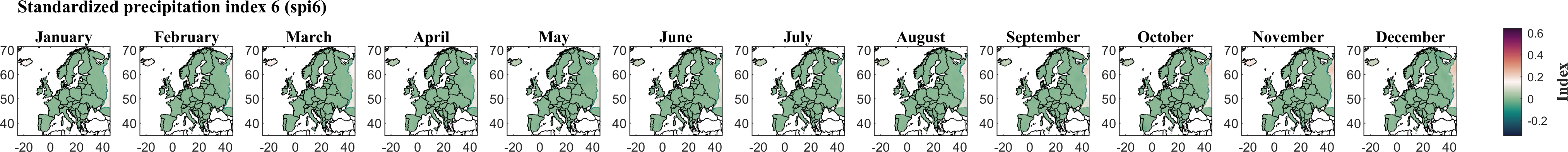

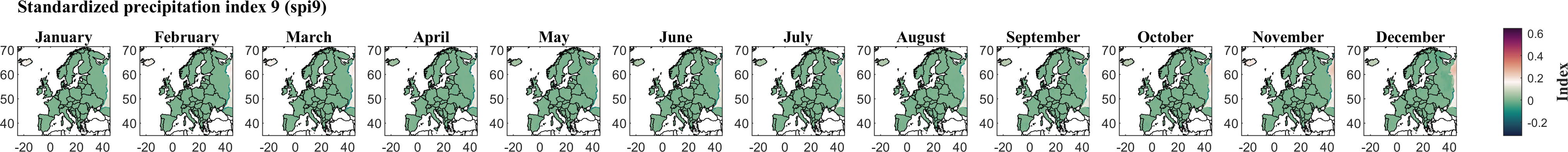

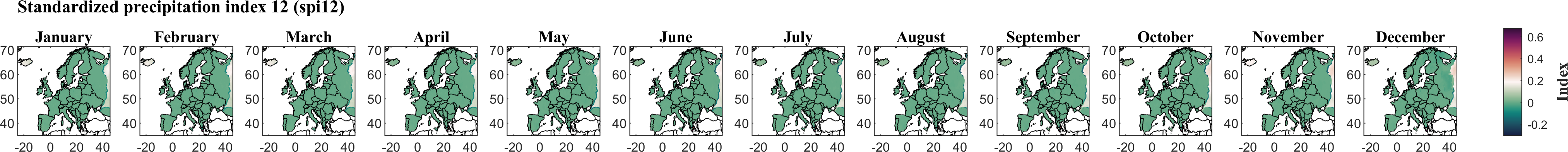

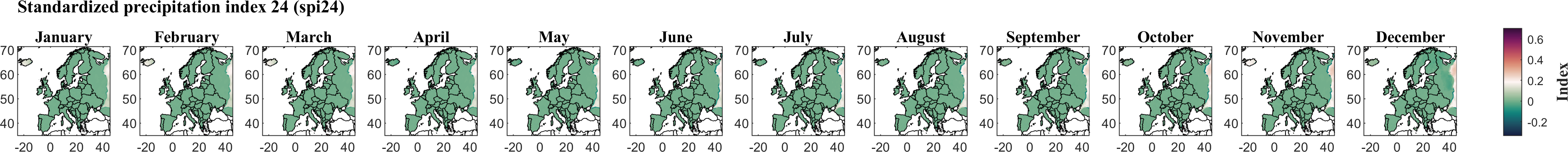

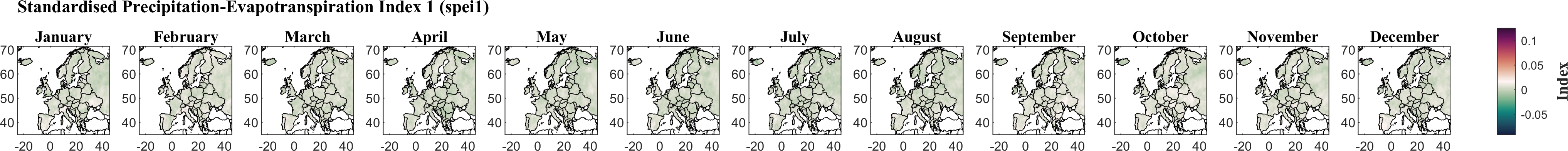

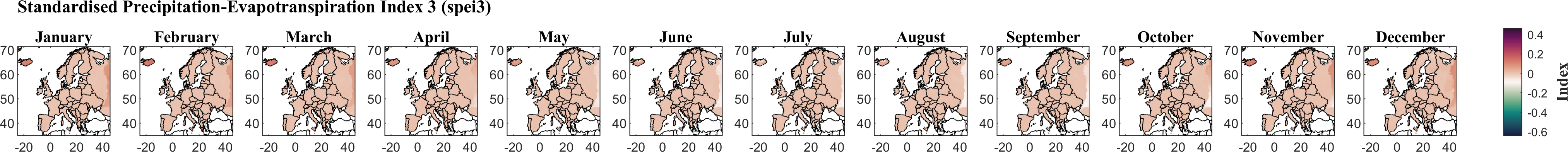

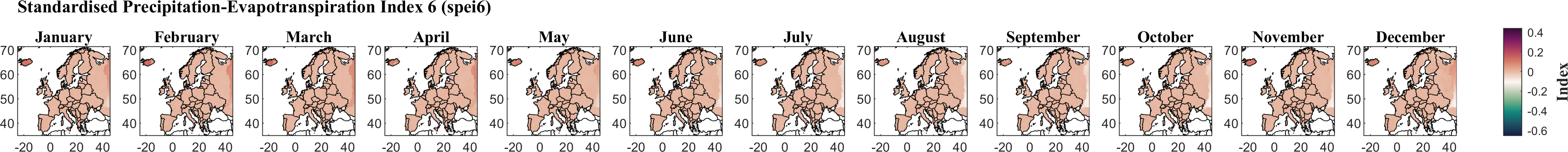

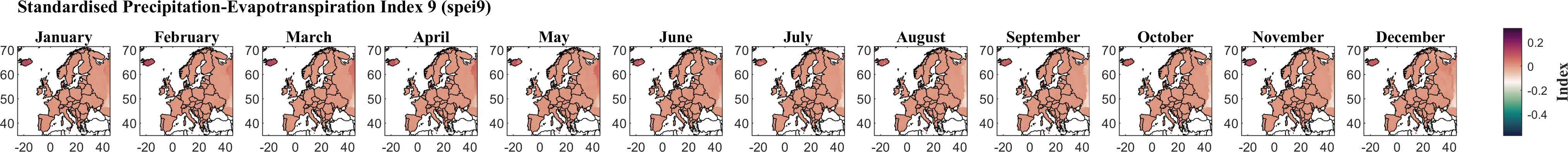

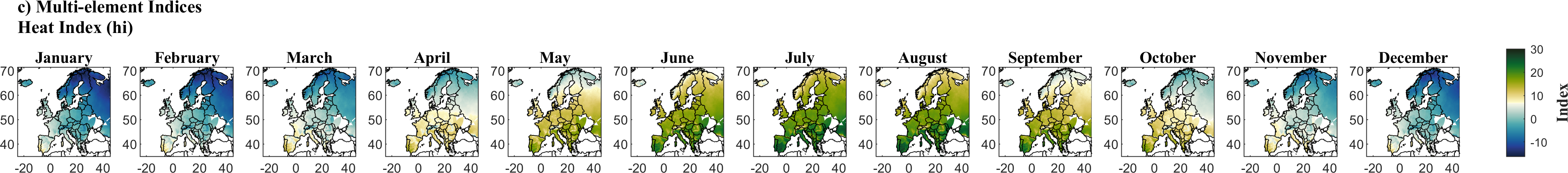

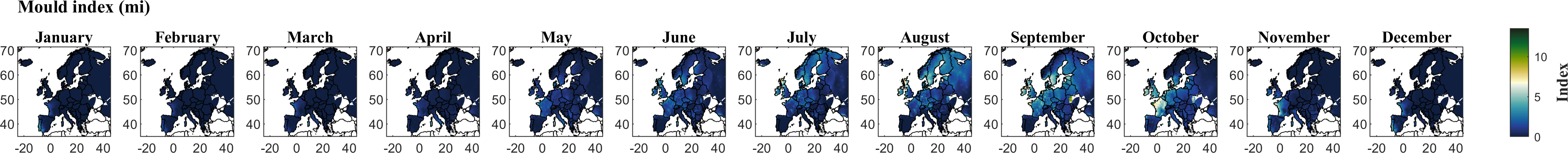

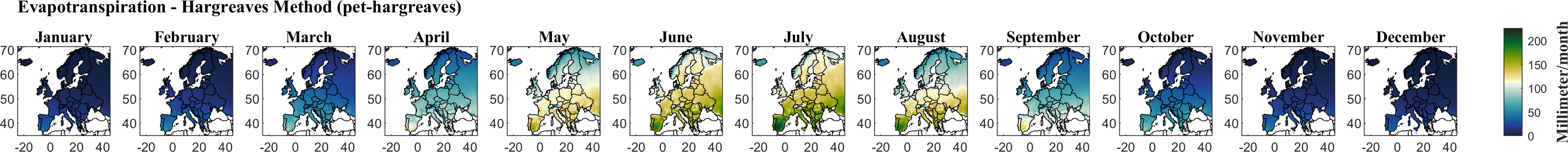

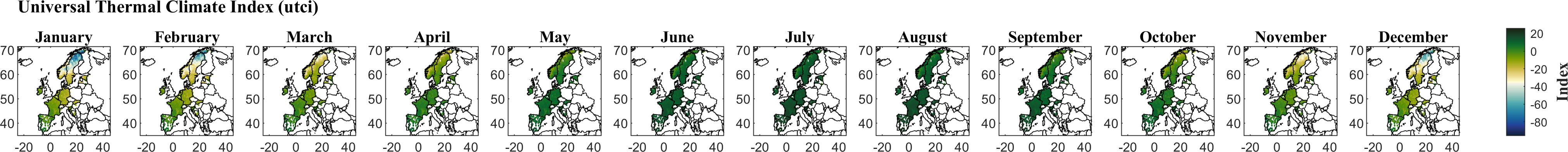

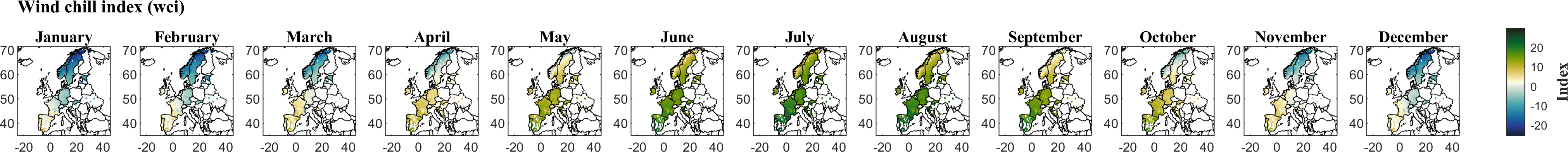

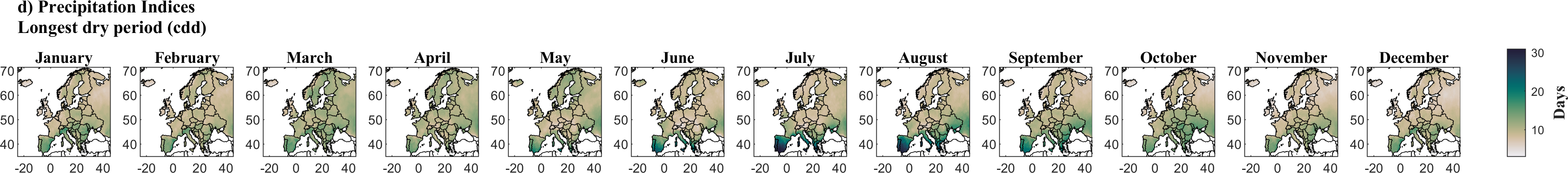

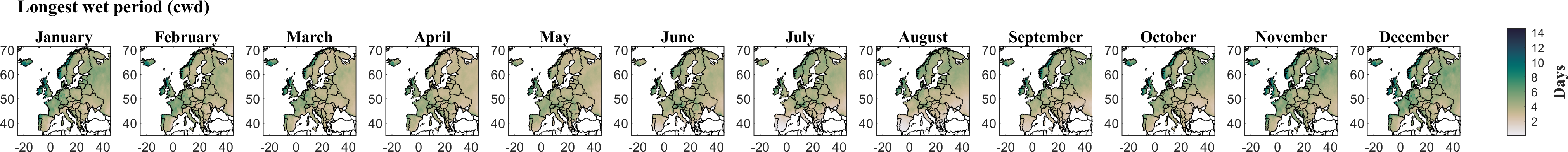

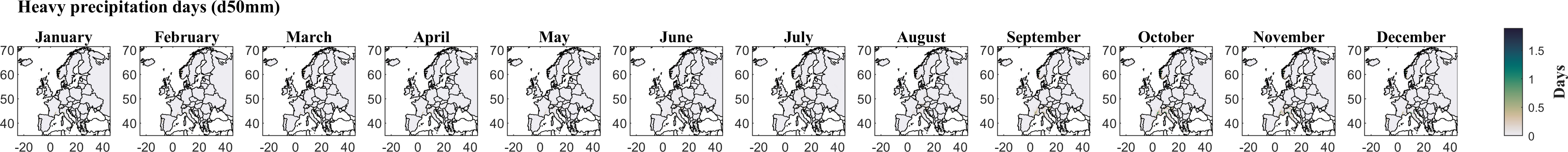

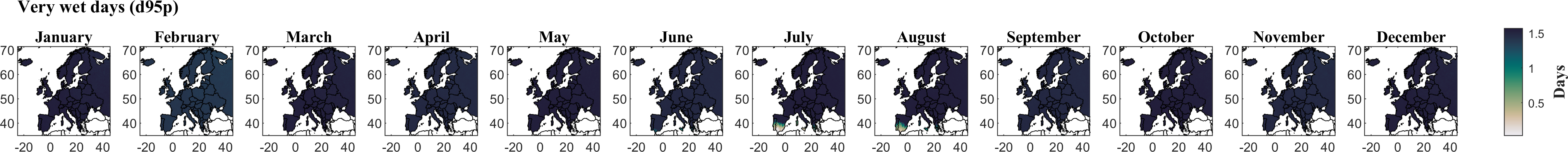

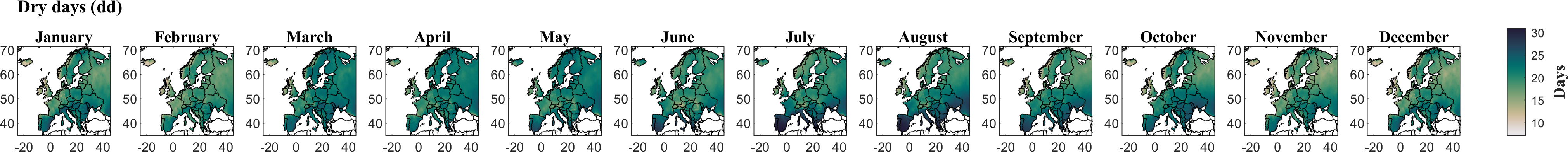

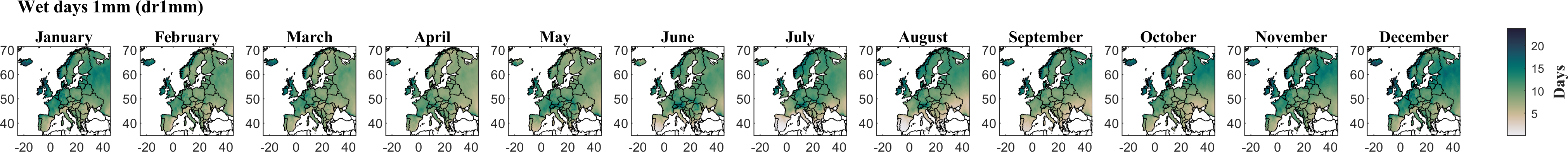

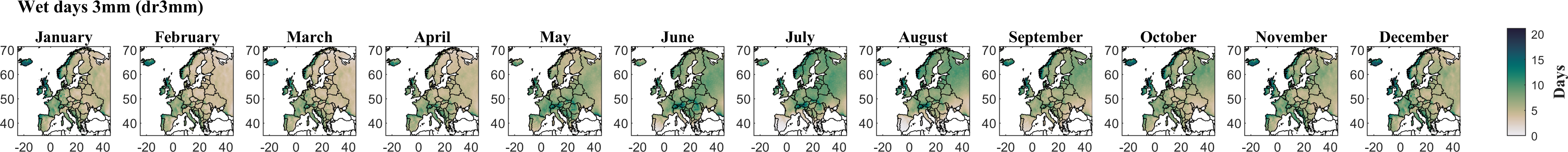

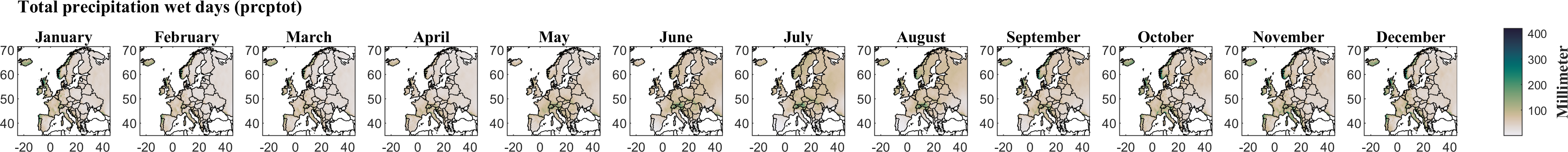

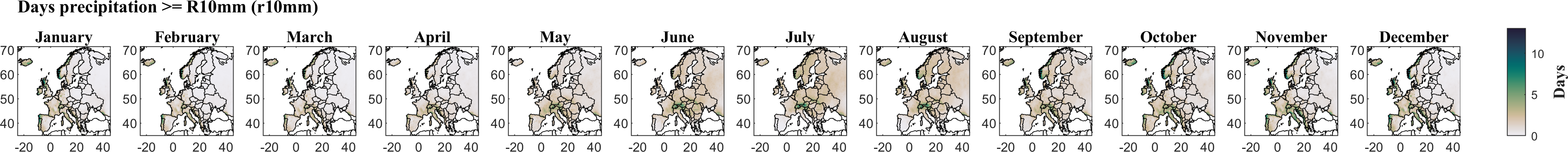

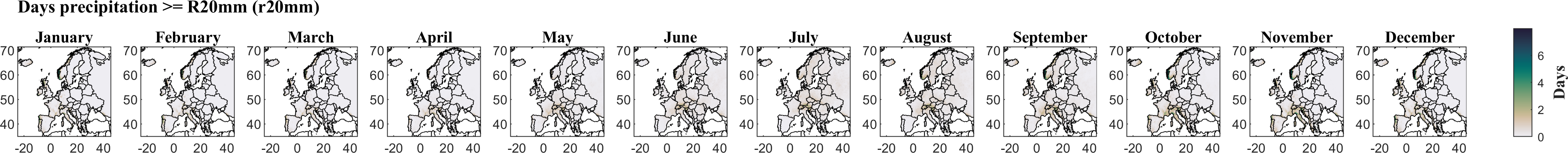

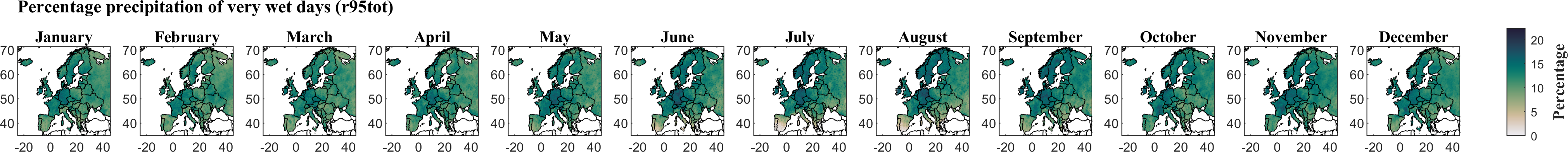

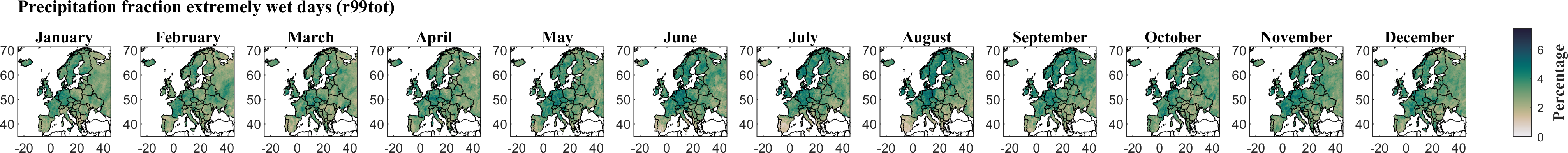

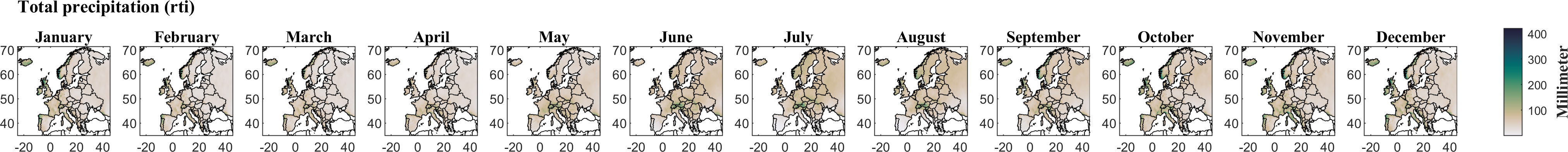

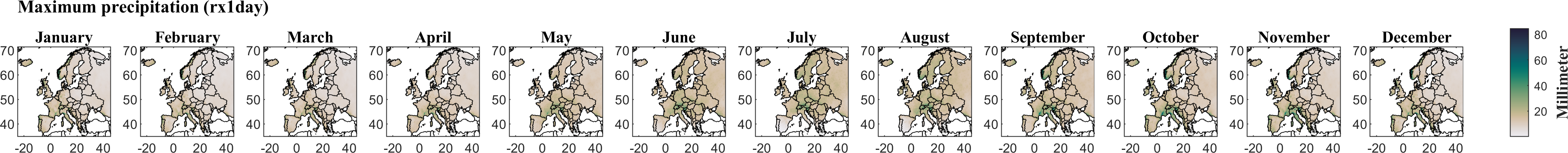

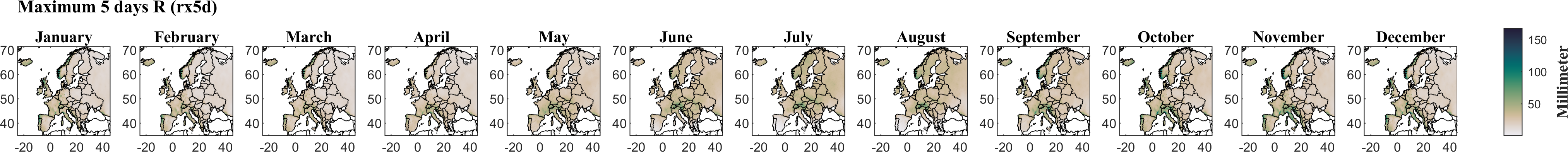

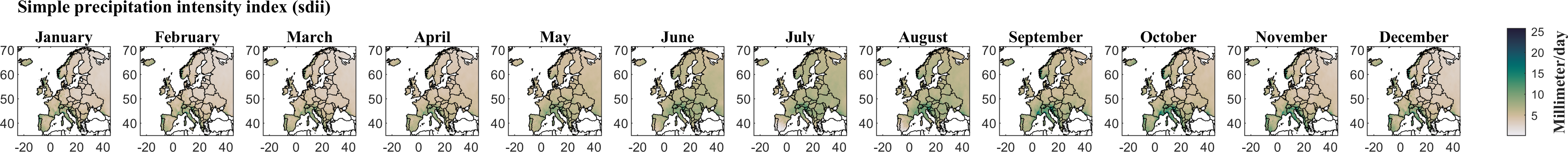

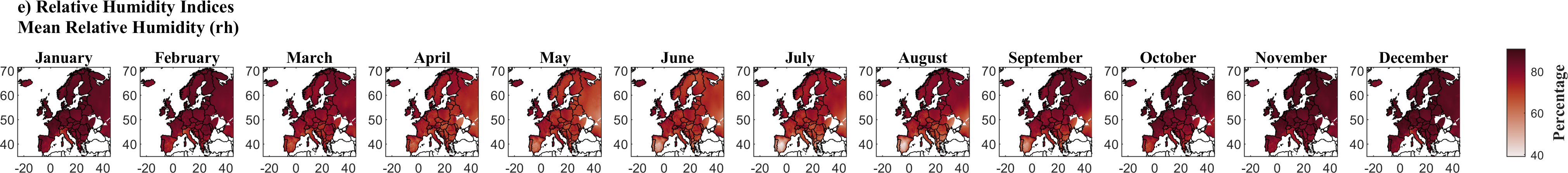

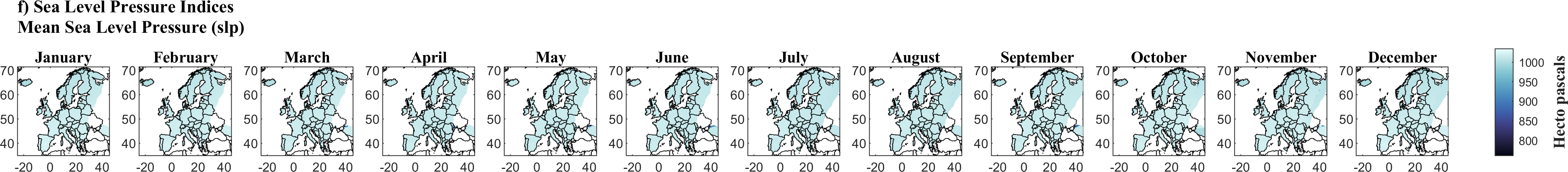

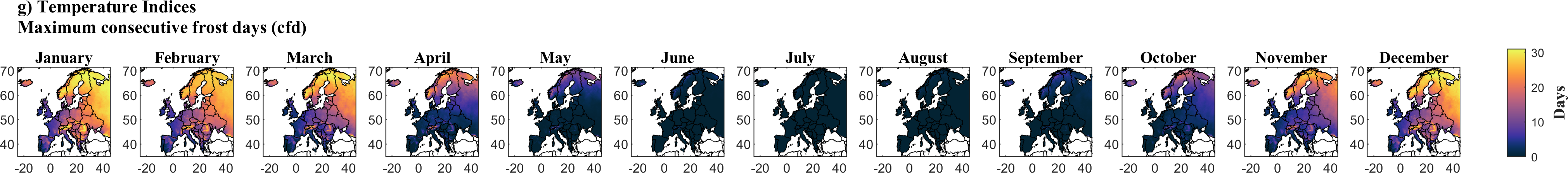

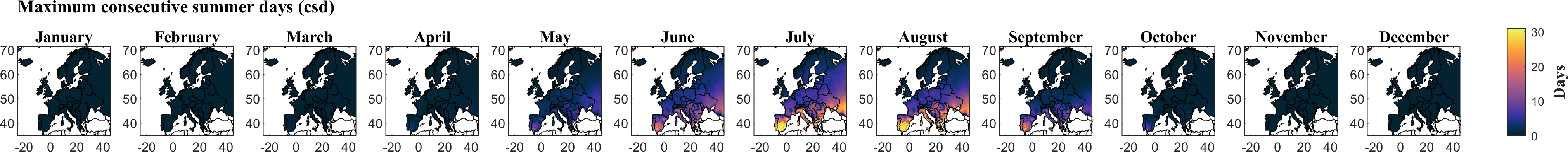

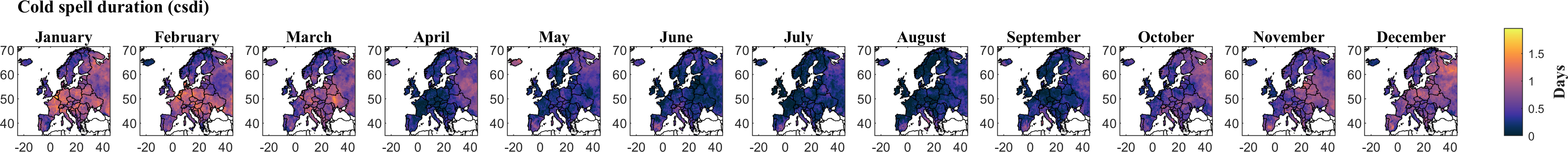

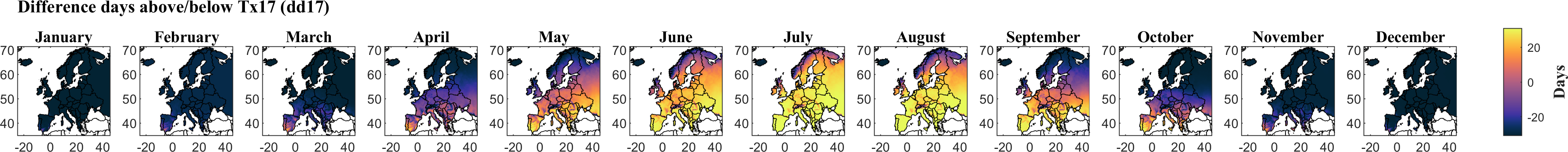

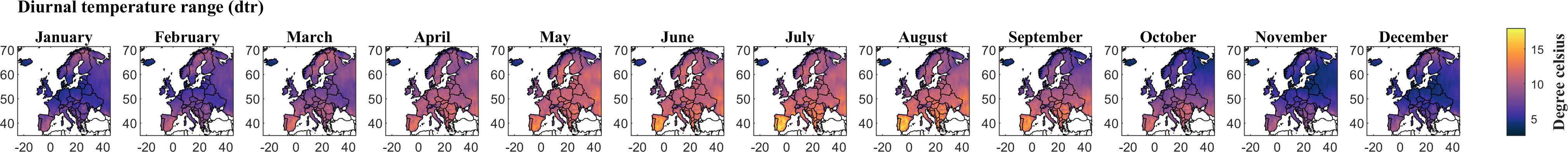

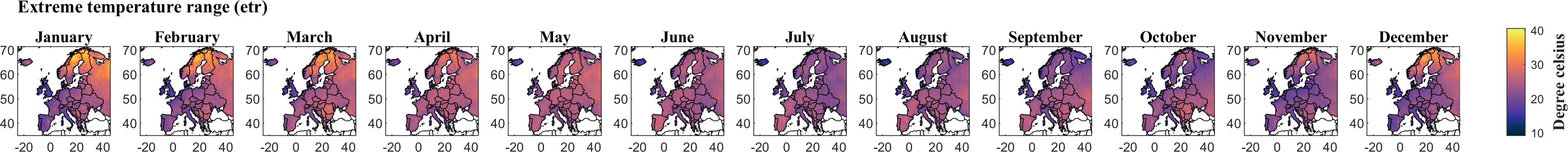

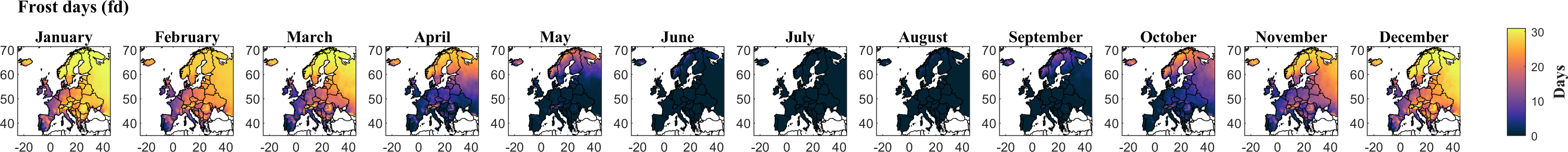

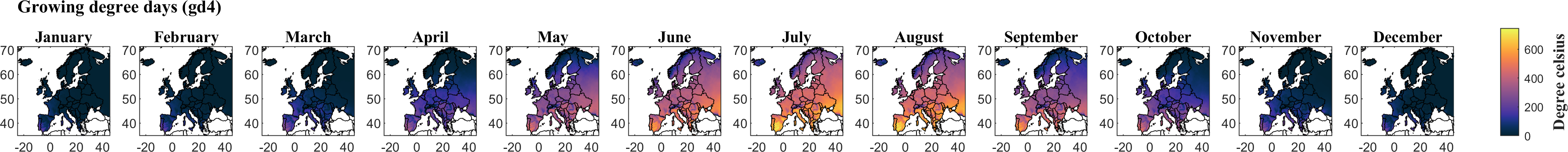

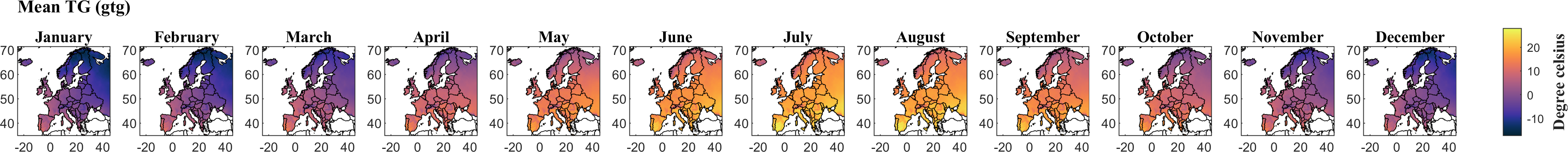

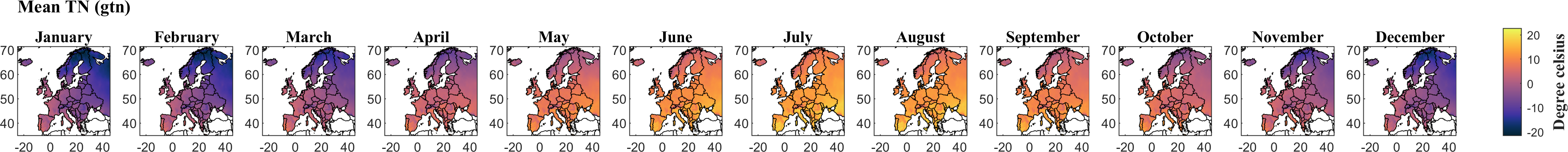

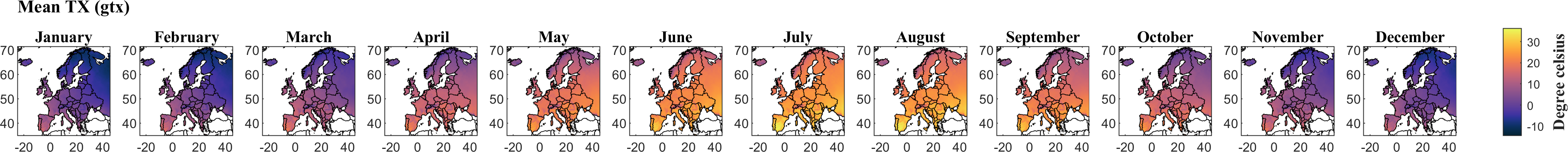

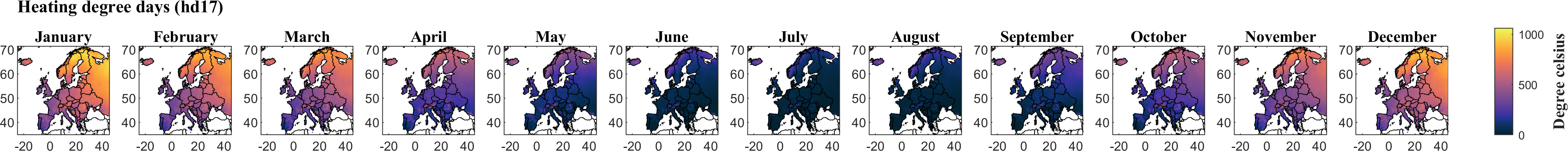

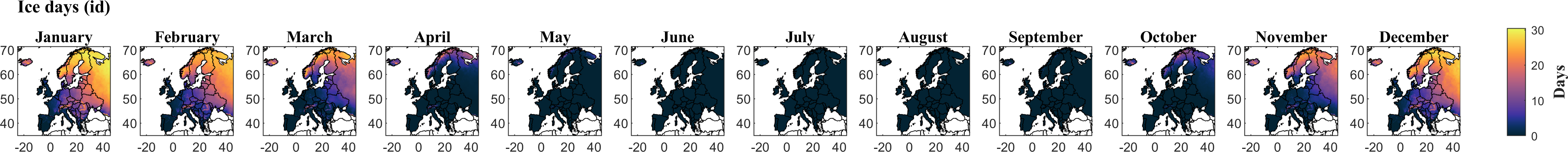

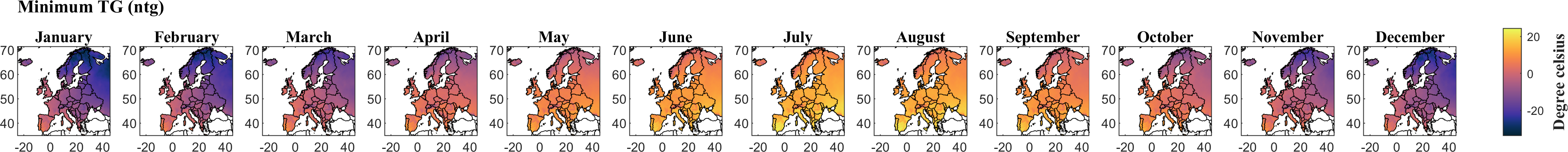

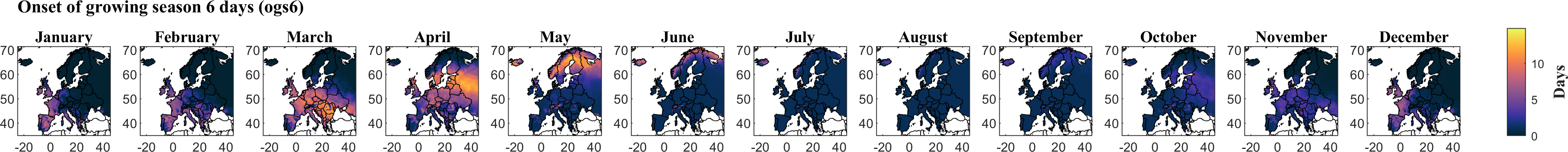

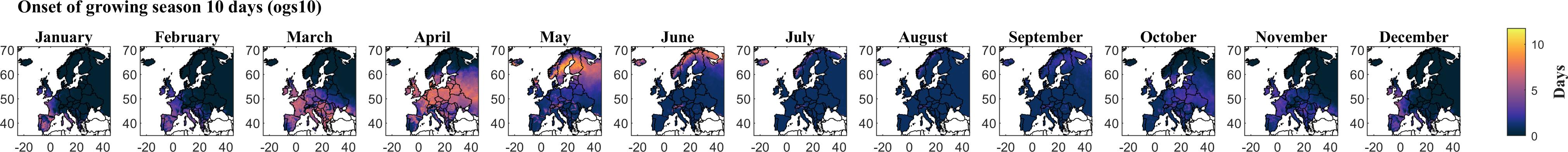

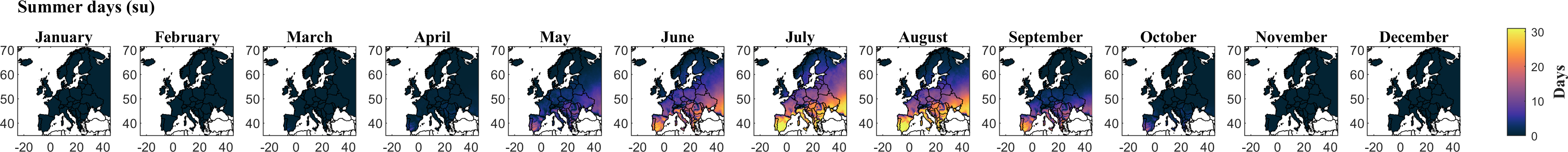

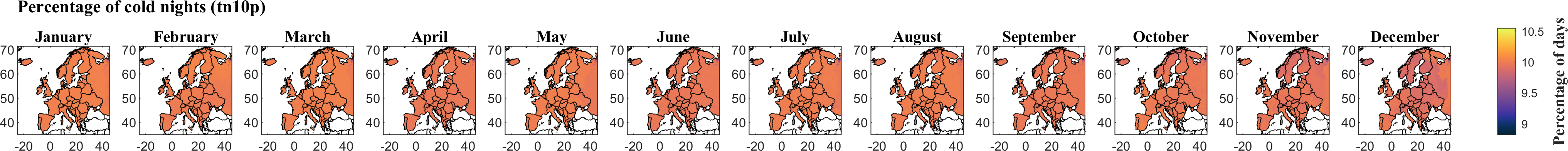

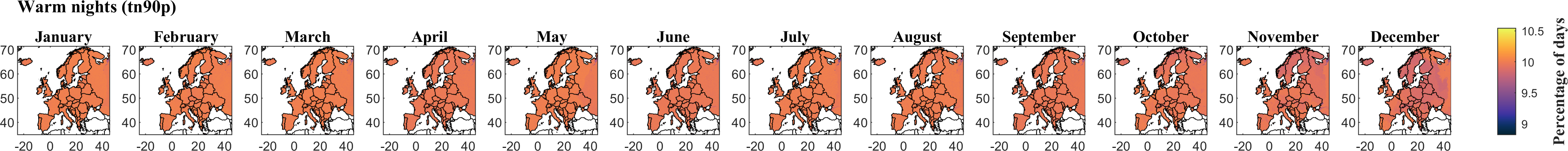

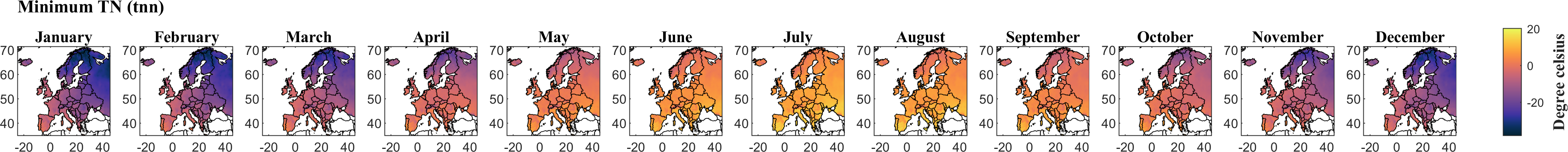

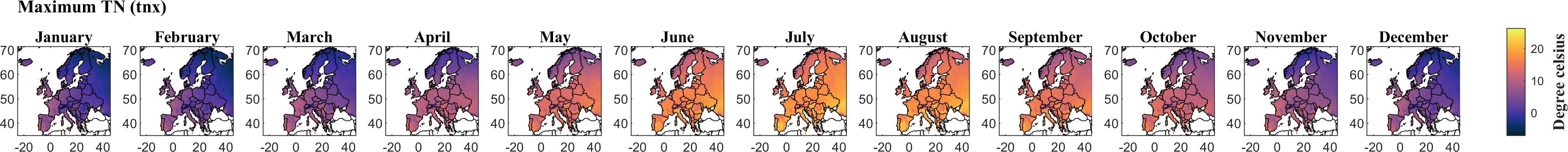

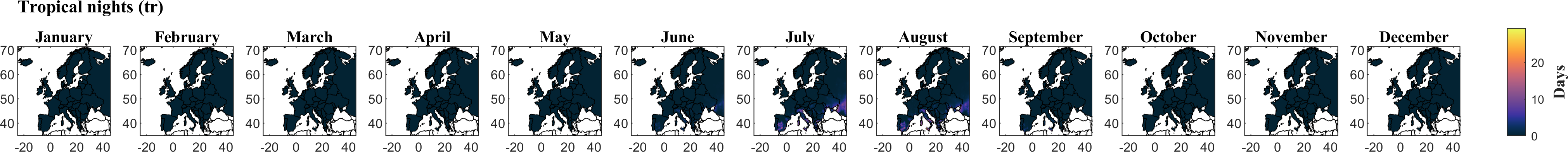

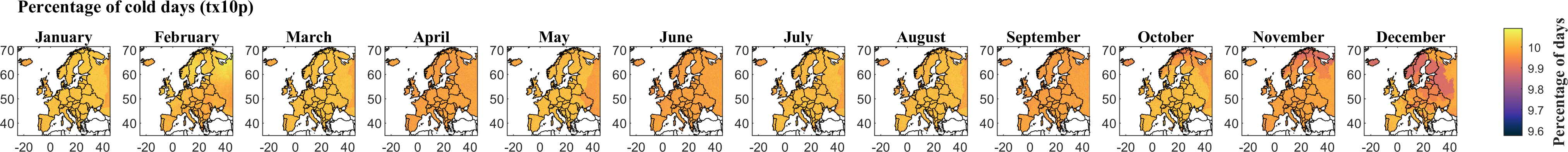

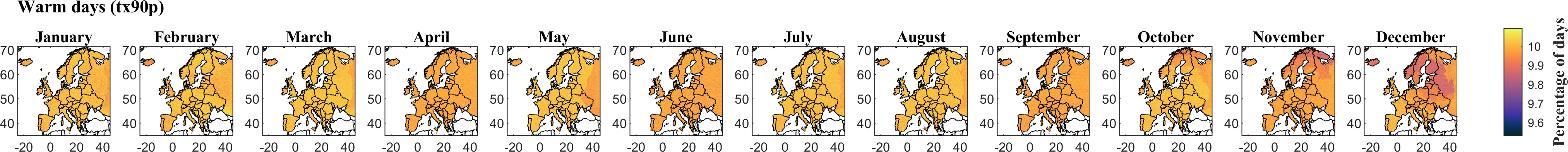

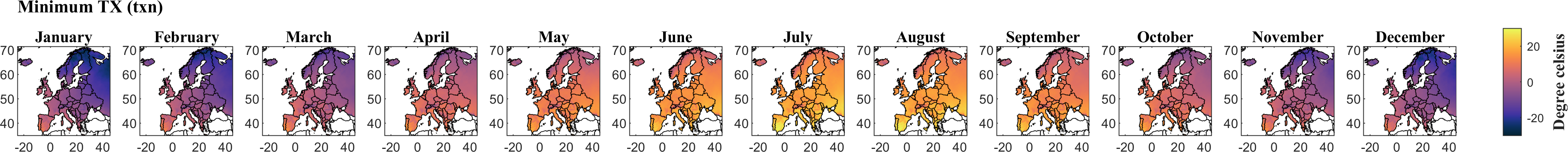

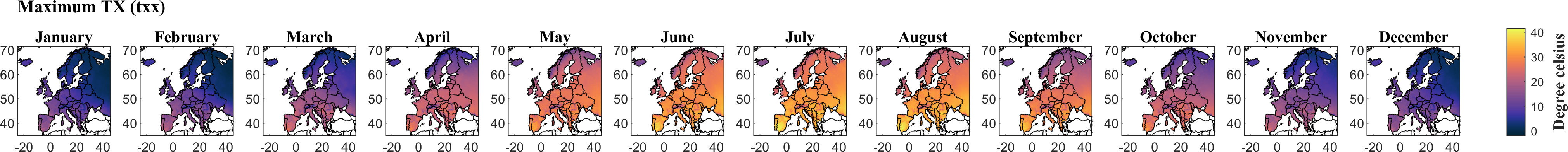

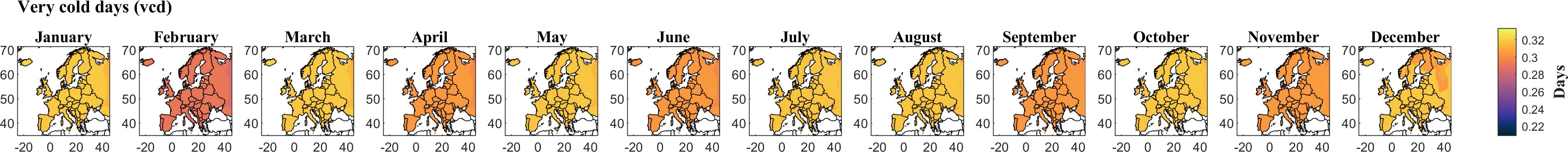

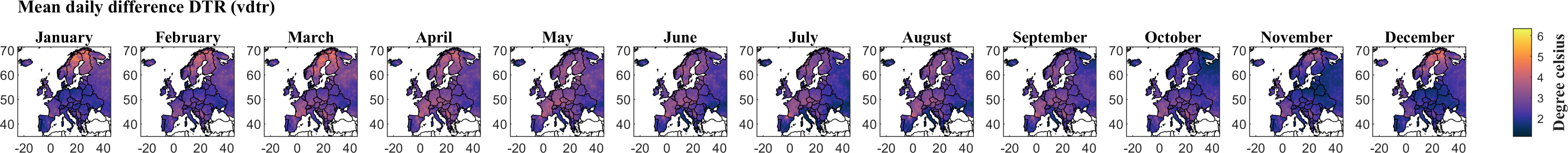

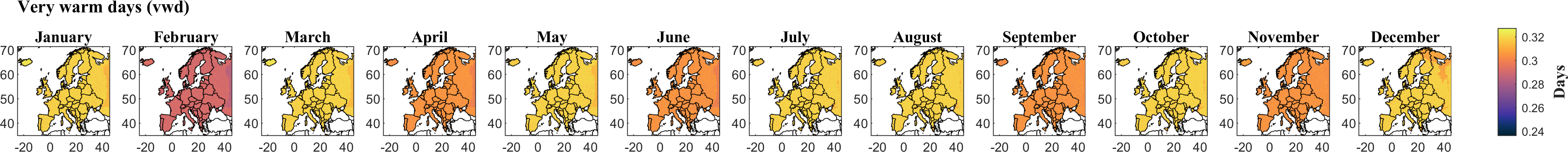

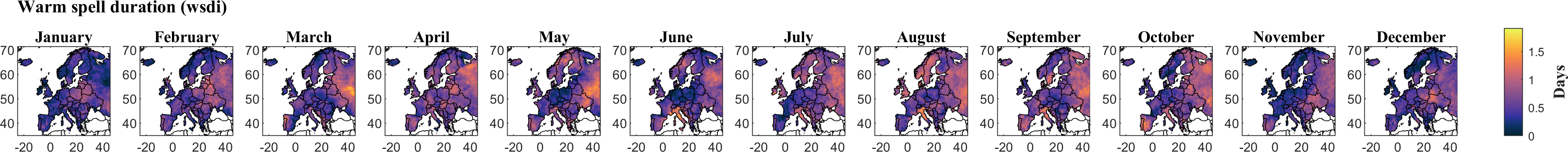

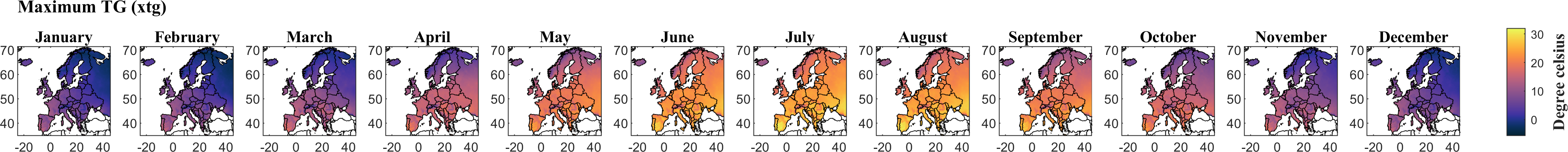

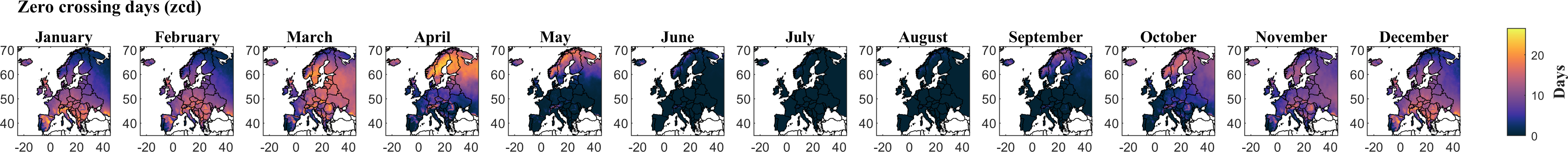

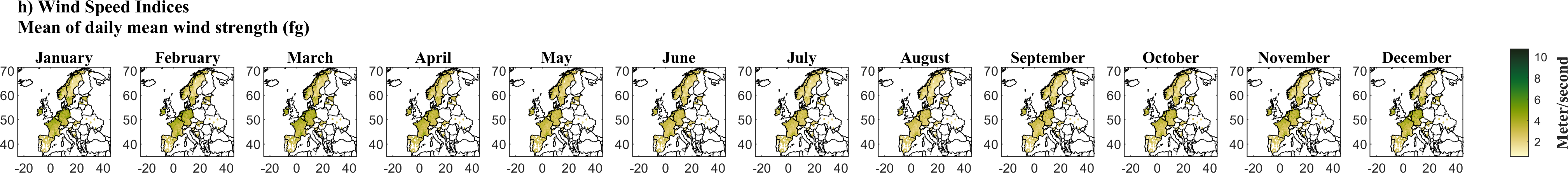

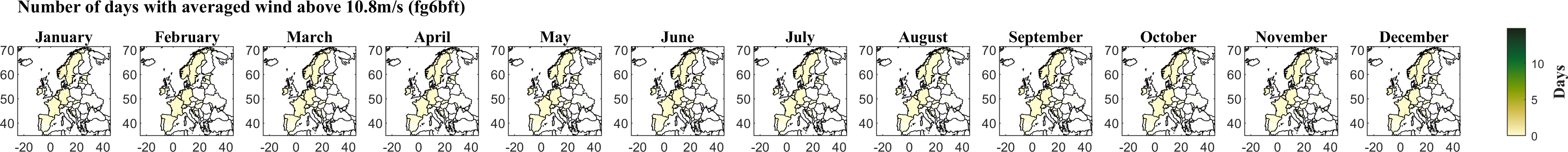

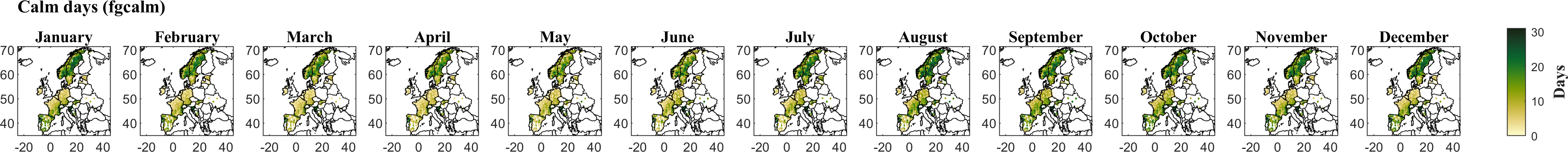


Figure S5: Monthly spatial variation of 74 climate indices categorized based on the weather variables used: a) Drought Indices b) Global Radiation c) Multi-element Indices d) Precipitation Indices e) Relative Humidity Indices f) Sea Level Pressure Indices g) Temperature Indices h) Wind Speed Indices.
