# Supplementary material for: Hotspot movement of compound events on the Europe continent
Source: Sci Rep. 2023 Oct 23;13:18100. doi: 10.1038/s41598-023-45067-6 (PMC10593787; doi:10.1038/s41598-023-45067-6)
Supplement: Supplementary file 6 — Supplementary Table S1. [file 41598_2023_45067_MOESM6_ESM.docx]

**Table S1-: A) Very high positive correlation > 0.8 extreme climate indices (I – seasonal II – monthly)** **B) Very high negative correlation < -0.8 extreme climate indices (I – seasonal II – monthly:**

**A)**

1. **Seasonal:**

| **Dec, Jan, Feb (DJF)** | **Mar, Apr, May (MAM)** | **Jun, Jul, Aug (JJA)** | **Sep, Oct, Nov (SON)** |
| --- | --- | --- | --- |
| pet_hargreaves, gtx, txn, txx, wci with slp | gtn, tnn, tnx with dd17 | csd, dd17, gd4, gtg, gtx, hi, pet_hargreaves, su, txn, wci with bio20 | gd4, gtg, gtn, gtx, hi, ntg, pet_hargreaves, slp, tnn, tnx, txn, txx, utci, wci, xtg with bio20 |
| gd4, gtx, gtg, hi, ntg, tnn, txn, txx, utci, wci, pet_hargreaves, slp, xtg with bio20 |  | gtn, tnn, tnx with dd17 | gtx, pet_hargreaves, txx, utci, wci, xtg with slp |
| cfd, fd with id |  | gtn, tnn, tnx with su |  |
| mi with wci |  | gtn, tnn, tnx with csd |  |

1. **Monthly:**

| **Jan** | **Feb** | **Mar** | **Apr** | **May** | **Jun** | **Jul** | **Aug** | **Sep** | **Oct** | **Nov** | **Dec** |
| --- | --- | --- | --- | --- | --- | --- | --- | --- | --- | --- | --- |
| gtg, hi, ntg with slp | gd4, gtg, hi, ntg, utci, xtg with slp | cfd, fd with id | cfd, fd with id | gtn, tnn, tnx with csd |  | cdd, dd, ntg, txx, utci, xtg with bio20 | csd, dd17 gtx, su with dd | cdd, gtn, tnn, tnx with dd17 | cfd, fd with id | cfd, fd with id | utci with slp |
| gtn with bio20 | dd17, gtn with bio20 | dd17, gd4, gtg, gtn, gtx, hi, ntg, pet, slp, tnn, tnx, txn, txx, utci, wci, xtg with bio20 | dd17, gd4, gtg, gtn, gtx, hi, ntg, pet, tnn, tnx, txn, txx, utci, wci, xtg with bio20 | gtn, tnn, tnx, with su |  | csd, su with cdd | csd, su with cdd | csd, dd17, dtr, su with bio20 | csd, dd17, dtr, su with bio20 | pet  , wci with mi |  |
|  | gtn, slp, tnn, tnx with dd17 | dd17, gd4, gtg, gtn, gtx, hi, ntg, pet, tnn, tnx, txn, txx, utci, wci, xtg with slp | gtn, tnn, tnx with su |  |  | csd, dd17 gd4, gtg, gtx, ntg, su, txn, wci with dd | cwd, dr1mm with rh | csd, gtx, txx, su with cdd | gtn, tnn, tnx with dd17 |  |  |
|  |  | mi with wci | mi with wci |  |  | dr1mm with hd17 | dd, dtr, gtn, ntg, slp, tnn, tnx, txx, utci, xtg with bio20 | csd, dd17, gd4, gtg1 hi, ntg, su with slp | dd17, dtr, gtg, hi with slp |  |  |
|  |  |  |  |  |  |  | dd17, gtx, txn, txx with tr | gtn, tnn, tnx with csd | gtn, tnn, tnx with csd |  |  |
|  |  |  |  |  |  |  |  | gtn, tnn, tnx with su | gtn, tnn, tnx with su |  |  |

**B)**

1. **Seasonal:**

| **Dec, Jan, Feb (DJF)** | **Mar, Apr, May (MAM)** | **Jun, Jul, Aug (JJA)** | **Sep, Oct, Nov (SON)** |
| --- | --- | --- | --- |
| hd17, id with bio20 | dd17, gtx, txn, txx with cfd |  | fd, hd17 with bio20 |
| gtx, txn, txx with cfd | dd17, gtx, txn, txx with fd |  | gtx, txn, txx with cfd |
| gtx, txn, txx with fd |  |  | gtx, txn, txx with fd |
| gtn, tnn, tnx with id |  |  |  |

1. **Monthly:**

| **Jan** | **Feb** | **Mar** | **Apr** | **May** | **Jun** | **Jul** | **Aug** | **Sep** | **Oct** | **Nov** | **Dec** |
| --- | --- | --- | --- | --- | --- | --- | --- | --- | --- | --- | --- |
| hd17, id with slp | cfd, fd with dd17 | cfd, fd, hd17, id with bio20 | cfd, fd, hd17 with bio20 | cfd, fd with csd | dd17, gtx, txn, txx with cfd | cwd, dr1mm, dr3mm, hd17, rh with bio20 | cwd, dr1mm, hd17, rh with bio20 | cfd, rh, zcd with bio20 | cfd, id, zcd with bio20 | bio20, gtn, tnn, tnx with id |  |
|  | hd17, id with slp | cfd, hd17, id with slp | cfd, fd with csd | cfd, fd with su | dd17, gtx, txn, txx with fd | cdd with rh | cdd with rh | cdd with rh | csd, dd17, su with cfd |  |  |
|  |  | gtn, tnn, tnx with id | cfd, fd with su |  |  | cwd, dr1mm, dr3mm, rh with csd | cwd, dr1mm, dr3mm, rh with csd | csd, dd17, su with cfd | csd, dd17, su with fd |  |  |
|  |  |  | gtn, tnn, tnx with id |  |  | dd17, gtx, su, txn, wci with cwd | gtx, su with cwd | csd, pet, su with rh | gtn, tnn, tnx with id |  |  |
|  |  |  |  |  |  | dd with hd17 | dd, dtr, gtx, pet, su, txx, xtg with rh | csd, dd17, su with fd | hd17 with slp |  |  |
|  |  |  |  |  |  | dd17, gd4, gtg, gtx, ntg, su, txn, wci with dr1mm | dd17, gtx, su with dr1mm | hd17 with slp |  |  |  |
|  |  |  |  |  |  | dd17, gd4, gtg, gtx, ntg, su, txn, wci with dr3mm | su with dr3mm |  |  |  |  |
|  |  |  |  |  |  | dtr, pet, su with rh |  |  |  |  |  |
