# Supplementary material for: Hotspot movement of compound events on the Europe continent
Source: Sci Rep. 2023 Oct 23;13:18100. doi: 10.1038/s41598-023-45067-6 (PMC10593787; doi:10.1038/s41598-023-45067-6)
Supplement: Supplementary file 7 — Supplementary Table S2. [file 41598_2023_45067_MOESM7_ESM.docx]

**Table S2: Detailed description of hotspots of compound events**

| Figure 2 marked zone | Compound event | Location | Month | Best fit Copula Method | Joint Probability (%) |
| --- | --- | --- | --- | --- | --- |
| 1 | bio20 & gd4 | Kiruna, Sweden | December | Gumbel | 97 |
|  | bio20 & utci |  | December | Student-t | 76 |
|  | bio20 & wci |  | December | Student-t | 76 |
|  | pet & utci | Northern Lapland, Finland | January | Gumbel | 94 |
|  | pet & wci |  | December | Gumbel | 94 |
| 2 | bio20 & tnn | Murmansk Oblast, Russia | October | Gaussian | 49 |
|  | hi & pet |  | December | Gumbel | 53 |
| 3 | bio20 & gd4 | Novgorod Oblast, Russia | January | Gumbel | 97 |
|  | bio20 & gtg | Tver Oblast, Russia | July | Gaussian | 49 |
|  | bio20 & gtn |  | July | Gaussian | 49 |
|  | cfd, txx & bio20 | Vologda Oblast, Russia | November | Gaussian | 51 |
| 4 | bio20 & hi | Zhytomyr Oblast, Ukraine | November | Student-t | 87 |
|  | hi & wci |  | August-November | Student-t | 87 |
|  | bio20, wci & hi | Cherkasy Oblast, Ukraine | November | Gumbel | 64 |
| 5 | bio20 & hd17 | Krasnodar Krai, Russia | July | Gumbel | 90 |
|  | bio20 & ntg |  | July | Gaussian | 49 |
|  | bio20 & tnx |  | October | Gaussian | 50 |
|  | bio20 & xtg |  | October | Gaussian | 50 |
|  | bio20 & txn | Adygea Republic, Russia | July | Gaussian | 50 |
|  | bio20 & xtg |  | August | Gaussian | 50 |
|  | fd, txx & bio20 | Krasnodar Krai, Russia | January | Gaussian | 52 |
| 6 | fd & txx | Mousoures, Greece | January | Gaussian | 52 |
|  | gtn & gtx | Likongremos, Greece | August | Gumbel | 51 |
|  | gtn & txn |  | August | Gumbel | 48 |
|  | gtn & txx | Messiniakos Kolpos, Greece | October | Gumbel | 48 |
|  | gtx & tnn | Likongremos, Greece | August | Gumbel | 49 |
|  | gtx & tnx | Messiniakos Kolpos, Greece | August | Gumbel | 48 |
|  | tnn & txn |  | January | Gumbel | 49 |
|  | tnn & txx | Mousoures, Greece | October | Gumbel | 47 |
|  | tnx & txn | Molai, Greece | August | Gumbel | 47 |
|  | tnx & txx | Likongremos, Greece | May | Gumbel | 49 |
|  | bio20, gtn & txx | Messiniakos Kolpos, Greece | December | Gumbel | 47 |
|  | bio20, tnn & txx |  | August | Gumbel | 46 |
|  | bio20, tnx & txn | Likongremos, Greece | August | Gumbel | 46 |
|  | bio20, tnx & txx | Messiniakos Kolpos, Greece | December | Gumbel | 48 |
|  | bio20, txn & gtn | Likongremos, Greece | August | Gumbel | 47 |
|  | bio20, txn & tnn | Kythira, Greece | July | Gumbel | 48 |
| 7 | cfd & txx | Inverness, United Kingdom | June | Gaussian | 80 |
|  | hi, pet & wci | Donegal, Ireland | September | Clayton | 50 |
