# Supplementary material for: Hotspot movement of compound events on the Europe continent
Source: Sci Rep. 2023 Oct 23;13:18100. doi: 10.1038/s41598-023-45067-6 (PMC10593787; doi:10.1038/s41598-023-45067-6)
Supplement: Supplementary file 8 — Supplementary Table S3. [file 41598_2023_45067_MOESM8_ESM.docx]

**Table S3: Statistical indicators value for the determined compound events**
