# Supplementary material for: Hotspot movement of compound events on the Europe continent
Source: Sci Rep. 2023 Oct 23;13:18100. doi: 10.1038/s41598-023-45067-6 (PMC10593787; doi:10.1038/s41598-023-45067-6)
Supplement: Supplementary file 9 — Supplementary Table S4. [file 41598_2023_45067_MOESM9_ESM.docx]

**Table S4: Detailed description of monthly shifts and trend analysis of hotspots of compound events**


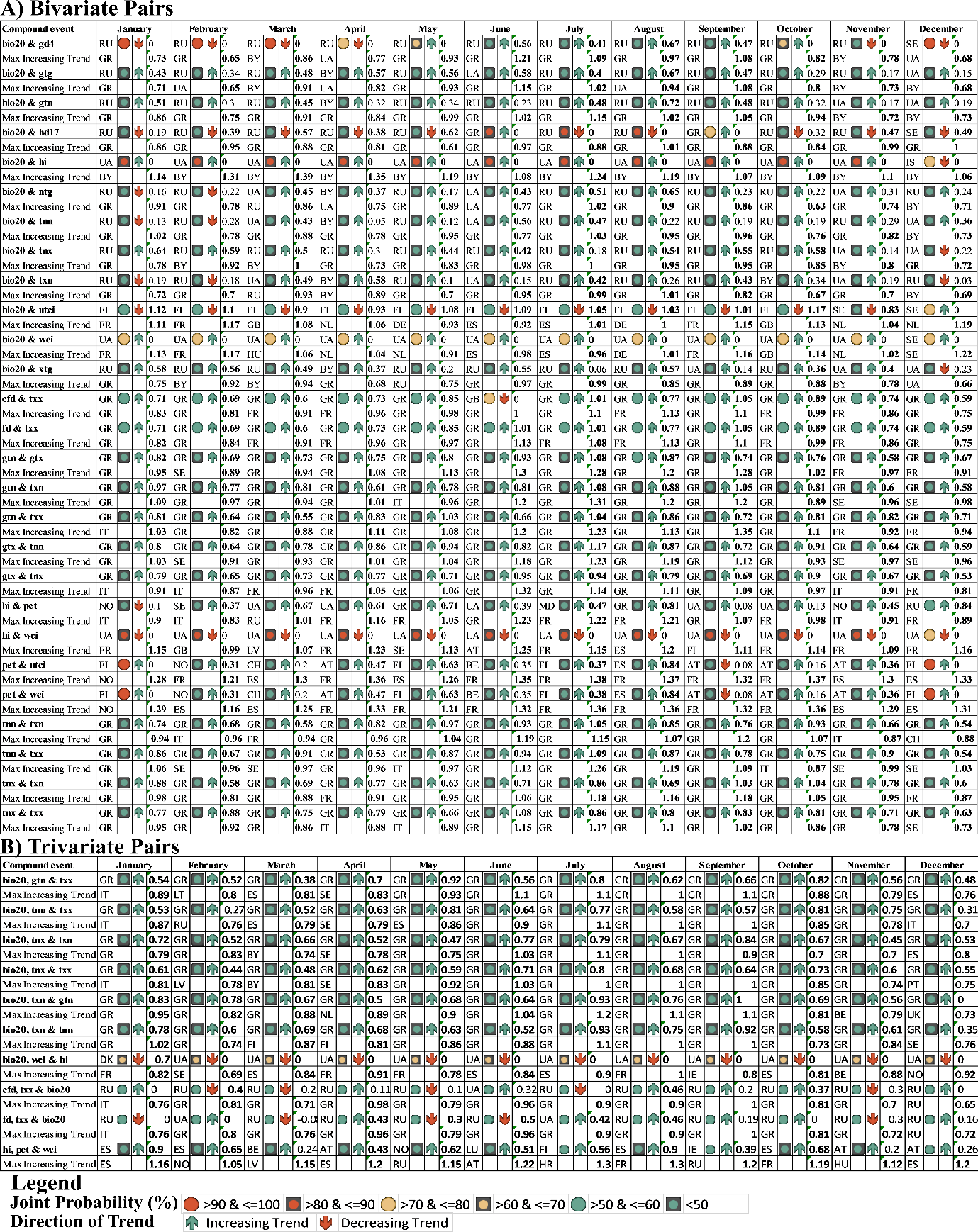


The circles and the colour represents the joint probability in %, arrows represents the direction of trend whether increasing or decreasing, and the values represent the magnitude of change in %/month. The country code is as per alpha-2 international standard ISO 3166. Bold value indicates that it is statistically significant (p-value is < 0.05).
