# Supplementary material for: Hotspot movement of compound events on the Europe continent
Source: Sci Rep. 2023 Oct 23;13:18100. doi: 10.1038/s41598-023-45067-6 (PMC10593787; doi:10.1038/s41598-023-45067-6)
Supplement: Supplementary file 1 — Supplementary Figure S1. [file 41598_2023_45067_MOESM1_ESM.docx]

**Figure S1: Correlation of extreme climate indices**

**A) Method 1: Pearson**

**B) Method 2: Kendall**

**C) Method 3: Spearman**

**D) Maximum of Pearson, Kendall and Spearman**

Figure S1: Comparison of various correlation methods to indicate the relation between different pairs of climate indices. A) Pearson B) Kendall C) Spearman and D) Maximum of Pearson, Kendall and Spearman. Dark brown and dark pink indicates strong positive correlation and shades of green indicate strong negative correlation between indices.
